# Ring Size Effects on the Structures of Sandwich Compounds with a Stoichiometry of C_12_H_12_M (M = Ti–Ni)

**DOI:** 10.1021/acs.organomet.4c00210

**Published:** 2024-11-18

**Authors:** Huidong Li, Ruilin Lu, Haoyu Chen, Jinfeng Luo, Qunchao Fan, R. Bruce King, Henry F. Schaefer

**Affiliations:** † School of Science, Key Laboratory of High Performance Scientific Computation, Xihua University, Chengdu 610039, China; ‡ Center for Computational Quantum Chemistry, University of Georgia, Athens, Georgia 30602, United States

## Abstract

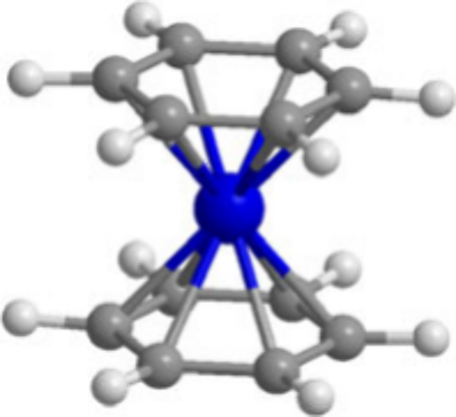

Ring size effects on geometries and electronic structures were investigated for the (C_*n*_H_*n*_)M(C_*m*_H_*m*_) (*n* = 4, 5, or 6; *m* = 8, 7, or 6; *m* + *n* = 12; M = Ti–Ni) systems using density functional theory. The lowest-energy C_12_H_12_M structures for the early transition metals titanium, vanadium, and chromium are the experimentally known singlet (η^5^-C_5_H_5_)Ti(η^7^-C_7_H_7_), doublet (η^5^-C_5_H_5_)V(η^7^-C_7_H_7_), and singlet (η^6^-C_6_H_6_)_2_Cr, respectively. The likewise experimentally known singlet (η^6^-C_6_H_6_)_2_Ti, doublet (η^6^-C_6_H_6_)_2_V, and singlet (η^5^-C_5_H_5_)Cr(η^7^-C_7_H_7_) are the second-lowest-energy structures with only a small energy difference between the two vanadium structures. For the later transition metals, dibenzenemetal complexes are the lowest-energy C_12_H_12_M species with two fully bonded hexahapto benzene rings in the lowest-energy manganese and iron derivatives and one hexahapto and one dihapto benzene ring in the lowest-energy cobalt and nickel derivatives. The lowest-energy (C_5_H_5_)M(C_7_H_7_) structures for the later transition metals iron, cobalt, and nickel have partially bonded nonplanar C_7_H_7_ rings with one or two uncomplexed C=C bonds. The (C_4_H_4_)M(C_8_H_8_) (M = Ti–Ni) structures with the metal sandwiched between four- and eight-membered rings were found to be much higher in energy than their (C_5_H_5_)M(C_7_H_7_) and (C_6_H_6_)_2_M isomers.

## 1 Introduction

The study of sandwich compounds originated from the serendipitous discovery of ferrocene (η^5^-C_5_H_5_)_2_Fe by two independent research groups in 1951.^1,2^ Soon thereafter, similar (η^5^-C_5_H_5_)_2_M sandwich compounds of the other first-row transition metals were synthesized. These bis(cyclopentadienyl)metal compounds, also called metallocenes, have distinctive structures in which the metal atom is sandwiched between two parallel planar pentagonal cyclopentadienyl rings.

A seminal development only a few years after the original synthesis of ferrocene was the synthesis of dibenzenechromium (η^6^-C_6_H_6_)_2_Cr by Fischer and Hafner.^3−5^ Not long thereafter, the closely related dibenzenevanadium (η^6^-C_6_H_6_)_2_V was synthesized by similar methods.^6^ The original syntheses of these two dibenzene sandwich compounds used the so-called “reducing Friedel-Crafts reaction” in which the anhydrous metal halide is allowed to react with benzene in the presence of aluminum chloride and aluminum metal as a reducing agent. The subsequent development of experimental methods for metal vapor synthesis provided a route to the corresponding titanium sandwich compound (η^6^-C_6_H_6_)_2_Ti from titanium vapor and benzene by Green and co-workers.^7,8^ Since the discovery of these original bies(benzene) metal complexes there have been diverse studies on their structures as well as substitution chemistry on the benzene rings.

Heteroleptic sandwich compounds of the type (η^5^-C_5_H_5_)M(η^7^-C_7_H_7_) with planar pentagonal and heptagonal rings are isomeric with the corresponding bis(benzene)metal compounds. The first such compound, which was first reported in 1959 by one of the authors of this paper (RBK) in collaboration with his doctoral thesis advisor, Gordon Stone, at Harvard University, was the vanadium derivative (η^5^-C_5_H_5_)V(η^7^-C_7_H_7_), obtained simply by heating (η^5^-C_5_H_5_)V(CO)_4_ with cycloheptatriene leading to complete loss of all four carbonyl groups.^9^ The sandwich geometry of (η^5^-C_5_H_5_)V(η^7^-C_7_H_7_) was confirmed shortly thereafter by Engebretson and Rundle using X-ray crystallography.^10^ The titanium and chromium analogues (η^5^-C_5_H_5_)M(η^7^-C_7_H_7_) (M = Ti^11^ and Cr)^12,13^ were subsequently synthesized, and the sandwich structure of (η^5^-C_5_H_5_)Ti(η^7^-C_7_H_7_) was confirmed by X-ray diffraction.^14^ In addition mixed sandwich compounds C_5_H_5_MC_7_H_7_ (M = Ti–Ni) of the first row transition metals were systematically investigated using density functional theory to give results consistent with the experimentally known geometries and spin states.^15^

The most stable sandwich compounds are those in which the central metal atom has the favored 18-electron configuration. For the C_12_H_12_M species of the first row (3d) transition metals this occurs with the chromium sandwiches (η^6^-C_6_H_6_)_2_Cr and (η^5^-C_5_H_5_)Cr(η^7^-C_7_H_7_). Similar neutral C_12_H_12_M species in which both carbocyclic ligands are planar and fully bonded to a central metal atom to the right of chromium in the Periodic Table, i. e. Mn, Fe, Co, and Ni, have metal configurations in excess of the favored 18-electron configuration. For these sandwich-type C_12_H_12_M compounds of the later transition metals, partial bonding of at least one ring is expected to be preferred thereby leaving one or more uncomplexed C=C bonds in the ring.

A continuing observation, even from the original 1959 study of (η^5^-C_5_H_5_)V(C_7_H_7_), was the significantly lower sensitivity of the (η^5^-C_5_H_5_)M(C_7_H_7_) derivatives toward air oxidation relative to their (η^6^-C_6_H_6_)_2_M isomers. This can be related to the formal oxidation states of the metal atoms in these molecules when the C_*n*_H_*n*_ rings have anionic structures with the preferred (4*k* + 2) π-electrons for stable aromatic planar rings to balance the expected positive or neutral oxidation states of the central metal atoms. Thus, the cyclopentadienyl rings are the 6 π-electron monoanions C_5_H_5_^–^, the benzene rings are the neutral species C_6_H_6_, but the cycloheptatrienyl rings are the 10 π-electron trianions C_7_H_7_^3–^. This makes the metals formally zerovalent in their dibenzene derivatives (η^6^-C_6_H_6_)_2_M but gives them the much higher formal oxidation state of +4 in their isomeric (η^5^-C_5_H_5_)M(η^7^-C_7_H_7_) derivatives. The lower sensitivity of the (η^5^-C_5_H_5_)M(η^7^-C_7_H_7_) derivatives toward oxidation arising from the higher formal metal oxidation state allows ring substitution chemistry to be done without destruction of the sandwich structure.

The central metal atoms in neutral C_12_H_12_M sandwich compounds of the later transition metals from manganese to nickel would have electron configurations exceeding the favorable 18-electron configuration if all 12 carbon atoms of both rings are bonded to the central metal atom. No such species are known that are isolable crystalline solids under normal laboratory conditions. However, sandwich compounds of marginal stability can be stabilized by full substitution of the external hydrogens in the planar polygonal carbon rings with methyl groups. This approach has allowed the synthesis of bis(hexamethylbenzene) sandwich compounds (Me_6_C_6_)_2_M (M = Fe or Co) of iron^16,17^ and cobalt^18^ that would have 20- and 21-electron configurations, respectively, of the metal atoms if all 12 carbon atoms of the two rings are involved in the metal bonding. The limited stability and air-sensitivity of these species so far have prevented definitive structural determinations by X-ray crystallography. The iron complex has a magnetic moment corresponding to a triplet spin state. It thus appears to have all 12 ring carbon atoms of the two hexamethylbenzene ligands involved in bonding to the iron atom as two hexahapto ligands. This gives the central iron atom a 20-electron configuration similar to that of the nickel atom in nickelocene (η^5^-C_5_H_5_)_2_Ni. However, the magnetic moment of the cobalt complex (Me_6_C_6_)_2_Co corresponds to only the single unpaired electron for a doublet spin state suggesting a 19-electron metal configuration rather than the three unpaired electrons for a quartet spin state 21-electron complex. This, as well as the significant dipole moment of (Me_6_C_6_)_2_Co, suggests that one of its hexamethyl ligands is only partially bonded to the central cobalt atom leaving one or two uncomplexed C=C bonds.

Another type of isomer of the (η^6^-C_6_H_6_)_2_M and (η^5^-C_5_H_5_)M(η^7^-C_7_H_7_) sandwich compounds are the (η^4^-C_4_H_4_)M(η^8^-C_8_H_8_) derivatives with square planar cyclobutadiene C_4_H_4_ rings and octagonal planar C_8_H_8_ rings. However, sandwiches of this type are unknown experimentally. Obvious reasons for the lack of such compounds as stable species in the reported literature include the following: (1) Lack of suitable reagents to introduce the η^4^-C_4_H_4_ ring in view of the instability of unsubstituted cyclobutadiene as well as strain in the C_4_H_4_ ring; (2) Difficulty of the metal bonding atomic orbitals to overlap efficiently with the orbitals of all eight carbon atoms of the larger cyclooctatetraene ring. In this connection, the computational studies reported in this paper show consistently the (C_4_H_4_)M(C_8_H_8_) derivatives to have much higher energies than their isomeric (C_5_H_5_)M(C_7_H_7_) and (C_6_H_6_)_2_M derivatives.

## 2 Theoretical Methods

The hybrid *meta*-GGA DFT method M06-L^19,20^ and the B3PW91 method^21,22^ as implemented in the Gaussian09 program^23^ were used for the computations. These methods have been reported to give better overall performance for organometallic compounds than the first-generation functionals.^24^ The results indicate that single-reference methods are appropriate for the present systems, although multireference character should be considered for systems having transition metal atom(s). Double-ζ plus polarization (DZP) basis sets were used in conjunction with the M06-L and B3PW91 method for the DFT optimizations. For carbon one set of pure spherical harmonic d functions with orbital exponent α_d_(C) = 0.75 was added to the standard Huzinaga–Dunning contracted DZ sets. This basis set is designated as (9s5p1*d*/4s2p1d).^25,26^ For hydrogen, a set of p polarization functions α_p_(H) = 0.75 was added to the Huzinaga–Dunning DZ sets. For the first row transition metals the Wachters’ primitive sets were used in our loosely contracted DZP basis sets, but augmented by two sets of p functions and one set of d functions, and contracted following Hood et al., and designated as (14s11p6*d*/10s8p3d).^27,28^

The geometries of all structures were fully optimized by using the above DFT method with the (120, 974) integration grid. Small imaginary frequencies for the optimized structures were assumed to originate from numerical integration errors. The optimized (C_*n*_H_*n*_)M(C_*m*_H_*m*_) (*n* = 6, 5, or 4; *m* = 6, 7, or 8; *n* + *m* = 12) structures in this paper are designated as **Mz-nmX**, where **M** is the chemical symbol of the transition metal atom, **z** is the relative energy structure sequence number predicted according to the M06-L method, **n** and **m** are the sizes of each ring, and **X** refers to the spin state with **S, D, T** and **Q**, representing the singlet, doublet, triplet and quartet spin states, respectively.

## 3 Results and Discussion

### 3.1 C_12_H_12_Ti Sandwiches

**Figure 1:**
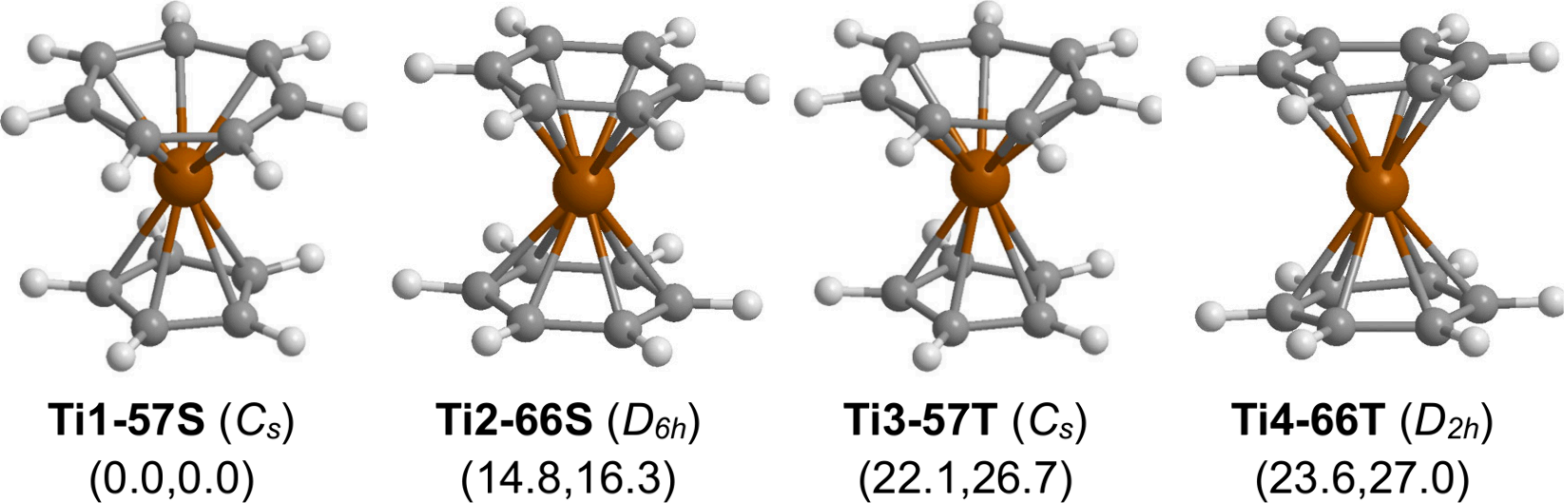
Optimized (C_*n*_H_*n*_)Ti(C_*m*_H_*m*_) (*n* = 6 or 5; *m* = 6 or 7; *n* + *m* = 12) structures. In Figures 1–12, the numbers in the parentheses are the relative energies (in kilocalories per mole) predicted by the B3PW91 method and the M06-L method.

Among the isomeric (C_*n*_H_*n*_)Ti(C_*m*_H_*m*_) (*n* = 6, 5, or 4; *m* = 6, 7, or 8; *n* + *m* = 12) compounds with different ring sizes, the experimentally known^11^ mixed sandwich isomer (η^5^-C_5_H_5_)Ti(η^7^-C_7_H_7_) **(Ti1–57S)** with two parallel rings has the lowest energy (Figure 1 and Table 1). This is consistent with the favorable oxidation state of +4 for the titanium atom sandwiched between the C_5_H_5_^–^ and C_7_H_7_^3–^ anions having the favorable 6 and 10 π-electrons, respectively. The second lowest-energy C_12_H_12_Ti sandwich isomer, lying 14.8 kcal/mol (B3PW91) or 16.3 kcal/mol(M06-L) above **Ti1–57S**, is the dibenzene complex (η^6^-C_6_H_6_)_2_Ti (**Ti2–66S**) in which the titanium atom is formally zerovalent. The mixed sandwich triplet structure (η^5^-C_5_H_5_)Ti(η^7^-C_7_H_7_) **(Ti3–57T)** lies 22.1 kcal/mol (B3PW91) or 26.7 kcal/mol (M06-L) in energy above the lowest-energy structure **Ti1–57S**. In addition the triplet dibenzenetitanium (η^6^-C_6_H_6_)_2_Ti structure **Ti4–66T** lies 23.6 kcal/mol (B3PW91) or 27.0 kcal/mol (M06-L) in energy above the lowest-energy **Ti1–57S** structure (Figure 1 and Table 1). The optimized (C_4_H_4_)Ti(C_8_H_8_) structures are predicted to lie at least 61.7 kcal/mol (B3PW91) or 56.7 kcal/mol (M06-L) higher in energy than the lowest-energy C_12_H_12_Ti sandwich structure **Ti1–57S** (Figure S1 and Table S1).

**Table 1:** Bond Distances (in angstroms), Relative Energies (Δ*E* in kilocalories per mole), Spin Expectation Values ⟨*S*^2^⟩, and HOMO–LUMO Gaps (H–L gaps, in electronvolts) for the (C_*n*_H_*n*_)Ti(C_*m*_H_*m*_) (*n* = 6 or 5; *m* = 6 or 7; *n* + *m* = 12) Structures

	**Ti1–57S** (*C*_*s*_)		**Ti2–66S** (*D*_6*h*_)		**Ti3–57T** (*C*_*s*_)		**Ti4–66T** (*D*_2*h*_)	
	B3PW91	M06-L	B3PW91	M06-L	B3PW91	M06-L	B3PW91	M06-L
M–C_*n*_H_*n*_	2.33	2.32	2.25	2.25	2.37	2.35	2.32	2.32
M–C_*m*_H_*m*_	2.21	2.20			2.31	2.31		
H–L gap	3.39	1.82	2.79	1.13	3.64	1.65	2.99	1.38
Δ*E*	0.0	0.0	14.8	16.3	22.1	26.7	23.6	27.0
⟨*S*^2^⟩	0.00	0.00	0.00	0.00	2.02	2.03	2.02	2.02

### 3.2 C_12_H_12_V Sandwiches

**Figure 2:**
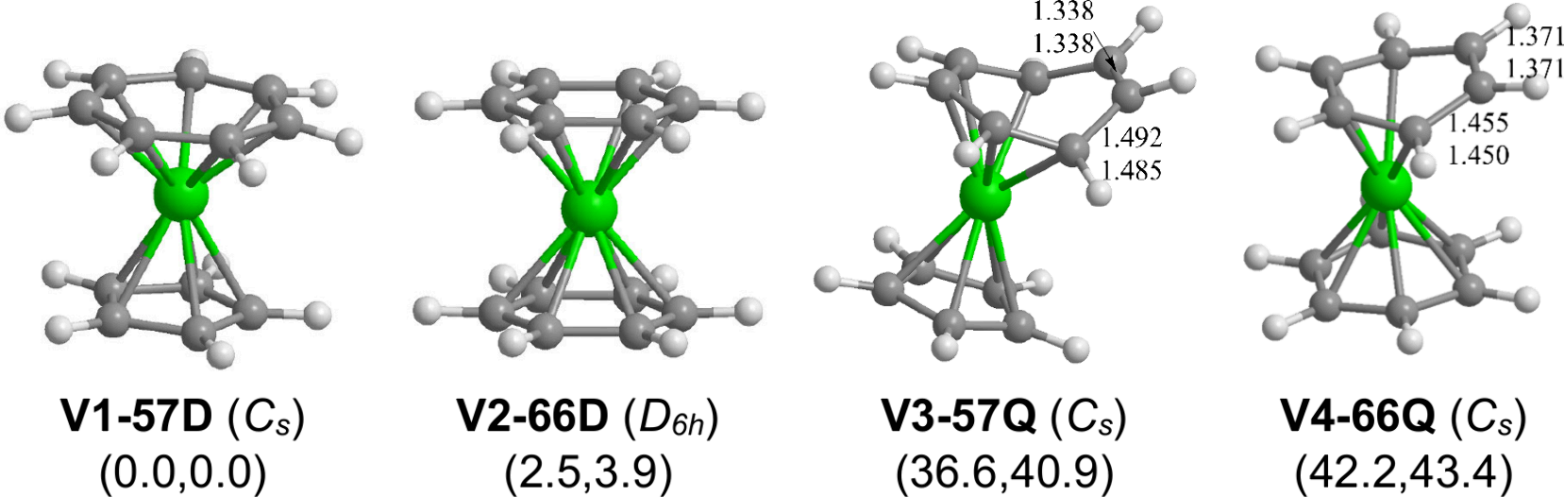
Optimized (C_*n*_H_*n*_)V(C_*m*_H_*m*_) (*n* = 6 or 5; *m* = 6 or 7; *n* + *m* = 12) structures.

The lowest-energy C_12_H_12_V (*n* = 6 or 5; *m* = 6 or 7; *n* + *m* = 12) sandwich structure is predicted to be the experimentally known^9,10,29^ doublet (η^5^-C_5_H_5_)V(η^7^-C_7_H_7_) **(V1–57D)** mixed sandwich structure having two parallel rings (Figure 2 and Table 2). Lying only slightly above **V1–57D** by 2.5 kcal/mol (B3PW91) or 3.9 kcal/mol (M06-L) is the experimentally known^6^ doublet *D*_6*h*_ dibenzenevanadium structure (η^6^-C_6_H_6_)_2_V (**V2–66D).** In both structures, the C_5_H_5_, C_6_H_6_, and C_7_H_7_ rings are bonded to the central vanadium atom as η^5^-pentahapto, η^6^-hexahapto, and η^7^-heptahapto ligands, respectively, to give the central vanadium atom a 17-electron configuration, consistent with their doublet spin states.

**Table 2:** Bond Distances (in angstroms), Relative Energies (Δ*E* in kilocalories per mole), Spin Expectation Values ⟨*S*^2^⟩, and HOMO–LUMO Gaps (H–L gaps, in electronvolts) for the (C_*n*_H_*n*_)V(C_*m*_H_*m*_) (*n* = 6 or 5; *m* = 6 or 7; *n* + *m* = 12) Structures

	**V1–57D** (*C*_*s*_)		**V2–66D** (*D*_6*h*_)		**V3–57Q** (*C*_*s*_)		**V4–66Q** (*C*_*s*_)	
	B3PW91	M06-L	B3PW91	M06-L	B3PW91	M06-L	B3PW91	M06-L
M–C_*n*_H_*n*_	2.26	2.25	2.20	2.19	2.30	2.28	2.35	2.33
M–C_*m*_H_*m*_	2.19	2.18			2.52	2.50		
H–L gap	5.01	2.93	4.36	2.84	3.83	2.27	2.69	1.30
Δ*E*	0.0	0.0	2.5	3.9	36.6	40.9	42.2	43.4
⟨*S*^2^⟩	0.81	0.81	0.78	0.79	3.78	3.79	3.82	3.82

The quartet spin state isomers of these C_12_H_12_V structures are predicted to lie at considerably higher energies than their doublet isomers (Figure 2 and Table 2). Thus, the quartet mixed sandwich compound (η^5^-C_5_H_5_)V(η^5^-C_7_H_7_) **(V3–57Q)** was predicted to lie 36.6 kcal/mol (B3PW91) or 40.9 kcal/mol (M06-L) in energy above its doublet analogue **V1–57D**. In **V3–57Q**, the cycloheptatrienyl ring functions as a pentahapto η^5^-C_7_H_7_ ligand, leaving an uncomplexed C=C bond of length 1.338 Å. A similar situation occurs in the quartet dibenzenevanadium (η^6^-C_6_H_6_)V(η^4^-C_6_H_6_) **(V4–66Q)**, lying 42.2 kcal/mol (B3PW91) or 43.4 kcal/mol (M06-L) above **V1–57D**. Thus, in **V4–66Q**, one benzene ring is predicted to be a η^4^-C_6_H_6_ tetrahapto ligand, leaving an uncomplexed C=C bond of length 1.371 Å. In these two quartet structures, the central vanadium atom acquires the 15-electron configuration, consistent with their quartet spin states. The lowest-energy (C_4_H_4_)V(C_8_H_8_) structure **V7–48D** was predicted to lie at the extremely high relative energy of 74.3 kcal/mol (B3PW91) or 68.8 kcal/mol(M06-L) above **V1–57D**.

### 3.3 C_12_H_12_Cr Sandwiches

**Figure 3:**
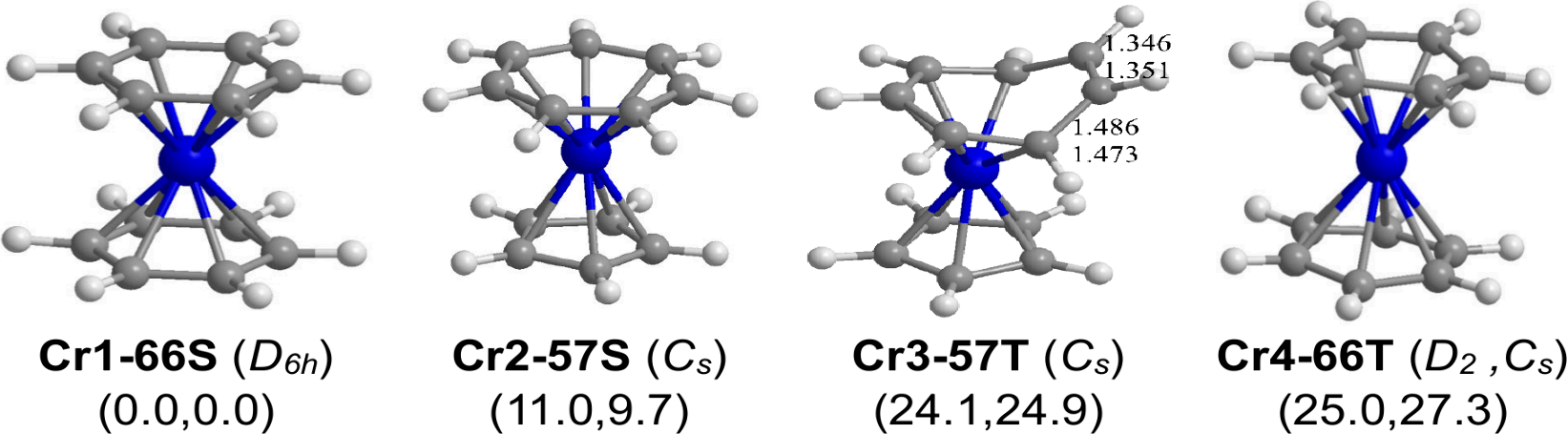
Optimized (C_*n*_H_*n*_)Cr(C_*m*_H_*m*_) (*n* = 6 or 5; *m* = 6 or 7; *n* + *m* = 12) structures.

The lowest-energy (C_*n*_H_*n*_)Cr(C_*m*_H_*m*_) (*n* = 6, 5, or 4; *m* = 6, 7, or 8; *n* + *m* = 12) structure is the singlet well-known dibenzenechromium^3−5^ (η^6^-C_6_H_6_)_2_Cr **(Cr1–66S)** structure having two parallel benzene rings fully coordinated to the central chromium atom (Figure 3 and Table 3). The next lowest-energy C_12_H_12_Cr isomer, lying 11.0 kcal/mol (B3PW91) or 9.7 kcal/mol (M06-L) above **Cr1–66S**, is the likewise experimentally known^12,13^ singlet mixed sandwich structure (η^5^-C_5_H_5_)Cr(η^7^-C_7_H_7_) **Cr2–57S** having parallel planar polygonal carbon rings. In both **Cr1–66S** and **Cr2–57S** both rings are fully bonded to the central chromium atom thereby giving it the favored 18-electron configuration.

**Table 3:** Bond Distances (in angstroms), Relative Energies (Δ*E* in kilocalories per mole), Spin Expectation Values ⟨*S*^2^⟩, and HOMO–LUMO Gaps (H–L gaps, in electronvolts) for the (C_*n*_H_*n*_)Cr(C_*m*_H_*m*_) (*n* = 6 or 5; *m* = 6 or 7; *n* + *m* = 12) Structures

	**Cr1–66S** (*D*_6*h*_)		**Cr2–57S** (*C*_*s*_)		**Cr3–57T** (*C*_*s*_)		**Cr4–66T** (*D*_2_, *C*_*s*_)	
	B3PW91	M06-L	B3PW91	M06-L	B3PW91	M06-L	B3PW91	M06-L
M–C_*n*_H_*n*_	2.15	2.13	2.19	2.17	2.23	2.22	2.24	2.23
M–C_*m*_H_*m*_			2.16	2.15	2.35	2.33		
H–L gap	4.06	2.25	4.00	1.98	4.31	2.44	3.04	1.08
Δ*E*	0.0	0.0	11.0	9.7	24.1	24.9	25.0	27.3
⟨*S*^2^⟩	0.00	0.00	0.00	0.00	2.13	2.17	2.22	2.21

The triplet spin state isomers of the singlet chromium sandwich compounds lie at considerably higher energies (Figure 3 and Table 3). The triplet mixed sandwich (η^5^-C_5_H_5_)Cr(η^5^-C_7_H_7_) **(Cr3–57T)**, lies 24.1 kcal/mol (B3PW91) or 24.9 kcal/mol (M06-L) in energy above **Cr1–66S**. The cycloheptatrienyl ring in **Cr3–57T** is a nonplanar pentahapto η^5^-C_7_H_7_ ring having an uncomplexed C=C bond of length ∼1.35 Å. This gives the central chromium atom a 16-electron configuration corresponding to the triplet spin state. However, the triplet dibenenechromium structure (η^6^-C_6_H_6_)Cr(η^6^-C_6_H_6_) **(Cr4–66T)**, lying 25.0 kcal/mol (B3PW91) or 27.3 kcal/mol (M06-L) above **Cr1–66S**, has both benzene rings functioning as η^6^-C_6_H_6_ hexahapto ligands to give the central chromium atom a 18-electron configuration similar to its singlet isomer. The lowest-energy (C_4_H_4_)Cr(C_8_H_8_) structure **Cr7–48T** is predicted to have the extremely high energy of 85.2 kcal/mol (B3PW91) or 79.5 kcal/mol (M06-L) relative to the lowest-energy structure **Cr1–66S**.

### 3.4 C_12_H_12_Mn Sandwiches

The energy surface for the (C_*n*_H_*n*_)Mn(C_*m*_H_*m*_) (*n* = 6, 5, or 4; *m* = 6, 7, or 8; *n* + *m* = 12) system is more complicated than that of the earlier first row transition metals since six sandwich structures with three different spin states (doublet, quartet, and sextet) were found within 25 kcal/mol of the lowest-energy structure. This lowest-energy C_12_H_12_Mn structure is the *D*_6*h*_ quartet (η^6^-C_6_H_6_)_2_Mn sandwich structure **(Mn1–66Q)** in which the manganese atom is sandwiched between two parallel planar benzene rings. Each benzene ring is coordinated to the central manganese atom as a η^6^-C_6_H_6_ hexahapto ligand. The second lowest-energy C_12_H_12_Mn structure, lying 5.6 kcal/mol (B3PW91) or 5.3 kcal/mol (M06-L) in energy above **Mn1–66Q**, is the doublet (η^6^-C_6_H_6_)_2_Mn **(Mn2–66D)**, likewise with two parallel planar hexahapto η^6^-C_6_H_6_ ligands. The quartet mixed sandwich (η^5^-C_5_H_5_)Mn(η^7^-C_7_H_7_) structure **(Mn3–57Q)**, lying 5.7 kcal/mol (B3PW91) or 5.5 kcal/mol (M06-L) in energy above **Mn1–66Q**, also has planar η^5^-C_5_H_5_ and η^7^-C_7_H_7_ rings fully coordinated to the central manganese atom through all of their carbon atoms. This leads to 19-electron configurations for the manganese atom in the three structures **Mn1–66Q**, **Mn2–66D**, and **Mn3–57Q**.

**Figure 4:**
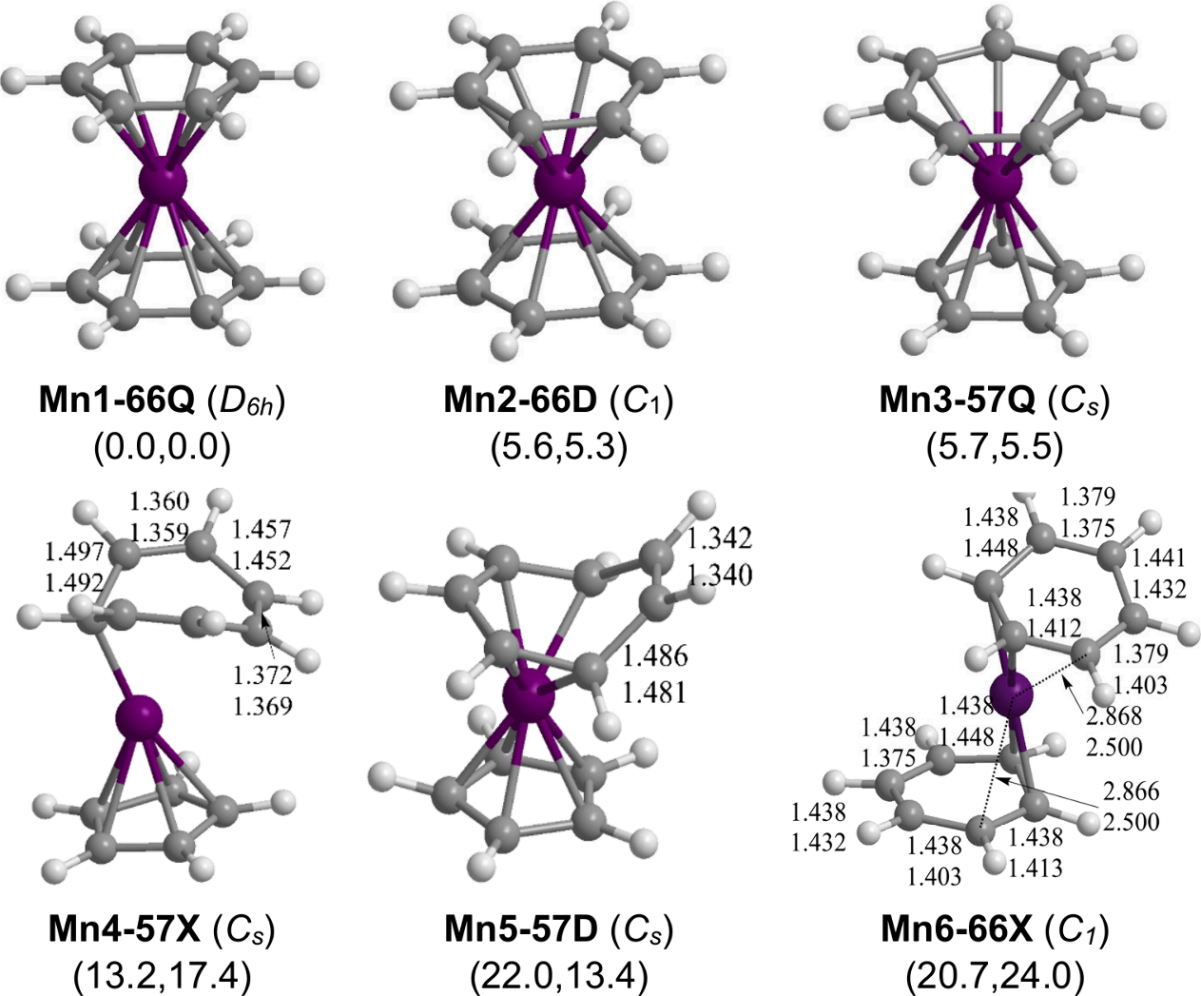
Optimized (C_*n*_H_*n*_)Mn(C_*m*_H_*m*_) (*n* = 6 or 5; *m* = 6 or 7; *n* + *m* = 12) structures.

The next three C_12_H_12_Mn structures in terms of energy have at least one of the rings only partially bonded to the central manganese atoms leading to nonplanar ring geometries having uncomplexed C=C bonds (Figure 4 and Table 4). This leads to manganese configurations less than the favorable 18-electron configuration. The sextet mixed sandwich (η^5^-C_5_H_5_)Mn(η^1^-C_7_H_7_) structure **Mn4–57X**, lying 13.2 kcal/mol (B3PW91) or 17.4 kcal/mol (M06-L) in energy above **Mn1–66Q**, has a planar cyclopentadienyl ring but a nonplanar cycloheptatrienyl η^1^-C_7_H_7_ ring bonded to the central manganese atom through only a single carbon atom. This leaves two uncomplexed C=C bonds of lengths ∼1.36 Å approximately 0.1 Å shorter than the remaining C–C bonds in the C_7_H_7_ ring. Both rings are pentahapto ligands in the doublet (η^5^-C_5_H_5_)Mn(η^5^-C_7_H_7_) structure **Mn5–57D**, lying 22.0 kcal/mol (B3PW91) or 13.4 kcal/mol (M06-L) above **Mn1–66Q**, thereby leading to a 17-electron configuration of the manganese atom. The η^5^-C_5_H_5_ ring in **Mn5–57D** is planar but the η^5^-C_7_H_7_ ring is nonplanar with an uncomplexed C=C bond of length 1.34 Å. The sextet dibenzenemanganese structure (η^2^-C_6_H_6_)_2_Mn (**Mn6–66X**) has two essentially dihapto benzene ligands but with a weaker interaction of a third carbon atom in each ring with the central manganese as suggested by Mn–C distances of ∼2.87 Å (B3PW91) or ∼2.50 Å (M06-L) that are long for even a single bond. The lowest-energy (C_4_H_4_)Mn(C_8_H_8_) structure is the quartet (η^4^-C_4_H_4_)Mn(η^4^-C_8_H_8_) structure **Mn7–48Q**, lying at the very high energy of 69.3 kcal/mol (B3PW91) or 60.1 kcal/mol M06-L) above **Mn1–66Q**.

**Table 4:** Bond Distances (in angstroms), Relative Energies (Δ*E* in kilocalories per mole), Spin Expectation Values ⟨*S*^2^⟩, and HOMO–LUMO Gaps (H–L gaps, in electronvolts) for the (C_*n*_H_*n*_)Mn(C_*m*_H_*m*_) (*n* = 6 or 5; *m* = 6 or 7; *n* + *m* = 12) Structures

	**Mn1–66Q** (*D*_6*h*_)		**Mn2–66D** (*C*_1_)		**Mn3–57Q** (*C*_*s*_)	
	B3PW91	M06-L	B3PW91	M06-L	B3PW91	M06-L
M–C_*n*_H_*n*_	2.31	2.31	2.18	2.17	2.41	2.40
M–C_*m*_H_*m*_					2.31	2.26
H–L gap	4.18	2.51	3.01	0.90	3.83	2.27
Δ*E*	0.0	0.0	5.6	5.3	5.7	5.5
⟨*S*^2^⟩	4.32	4.30	0.82	0.82	4.86	4.61

	**Mn4–57X** (*C*_*s*_)		**Mn5–57D** (*C*_*s*_)		**Mn6–66X** (*C*_*1*_)	
	B3PW91	M06-L	B3PW91	M06-L	B3PW91	M06-L
M–C_*n*_H_*n*_	2.38	2.35	2.13	2.14	2.79	2.60
M–C_*m*_H_*m*_	2.91	2.89	2.31	2.32		
H–L gap	3.6	2.09	5.01	2.93	2.11	1.08
Δ*E*	13.2	17.4	22.0	13.4	20.7	24.0
⟨*S*^2^⟩	8.78	8.78	0.80	0.79	8.81	8.89

### 3.5 C_12_H_12_Fe Sandwiches

**Figure 5:**
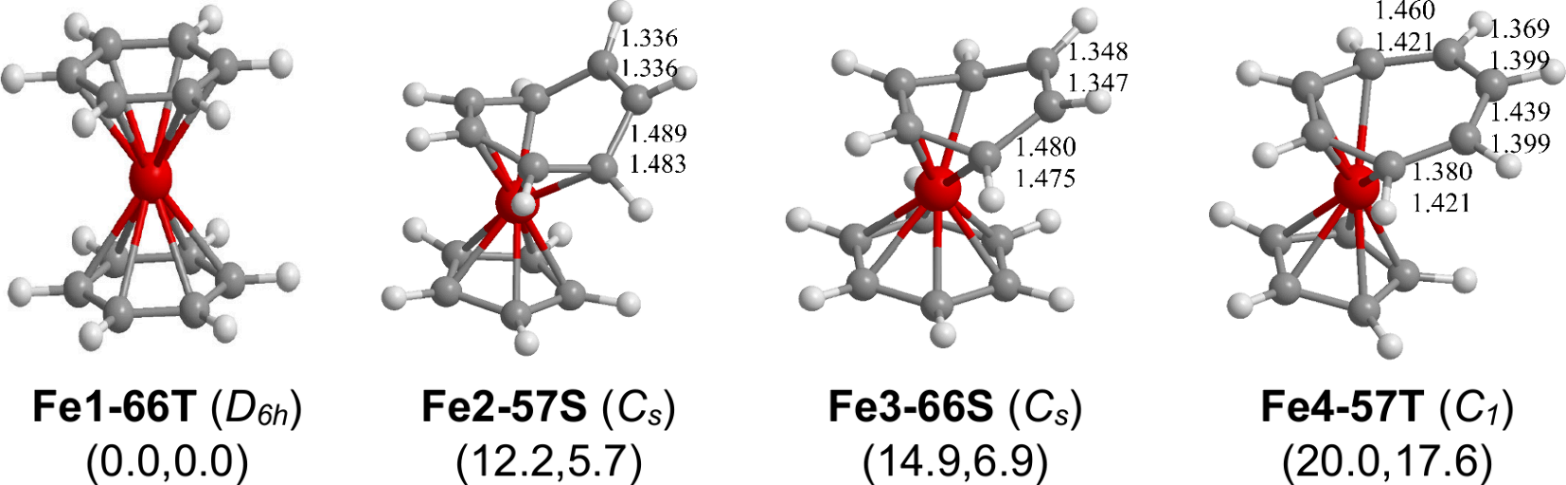
Optimized (C_*n*_H_*n*_)Fe(C_*m*_H_*m*_) (*n* = 6 or 5; *m* = 6 or 7; *n* + *m* = 12) structures.

The lowest-energy (C_*n*_H_*n*_)Fe(C_*m*_H_*m*_) (*n* = 6, 5, or 4; *m* = 6, 7, or 8; *n* + *m* = 12) structure is the triplet dibenzeneiron (η^6^-C_6_H_6_)_2_Fe structure **Fe1–66T** having two parallel hexahapto benzene rings (Figure 5 and Table 5). This leads to a 20-electron configuration for the central iron atom in **Fe1–66T** similar to the 20-electron configuration of the nickel atom in the well-known stable nickelocene^30−32^ (η^5^-C_5_H_5_)_2_Ni. This theoretical prediction is consistent with the experimental observation that bis(hexamethylbenzene)iron is a triplet spin state species.^16,17^

**Table 5:** Bond Distances (in angstroms), Relative Energies (Δ*E* in kilocalories per mole), Spin Expectation Values ⟨*S*^2^⟩, and HOMO–LUMO Gaps (H–L gaps, in electronvolts) for the (C_*n*_H_*n*_)Fe(C_*m*_H_*m*_) (*n* = 6 or 5; *m* = 6 or 7; *n* + *m* = 12) Structures

	**Fe1–66T** (*D*_6*h*_)		**Fe2–57S** (*C*_*s*_)		**Fe3–66S** (*C*_*s*_)		**Fe4–57T** (*C*_1_)	
	B3PW91	M06-L	B3PW91	M06-L	B3PW91	M06-L	B3PW91	M06-L
M–C_*n*_H_*n*_	2.26	2.25	2.08	2.06	2.21	2.20	2.20	2.16
M–C_*m*_H_*m*_			2.31	2.30			2.56	2.44
H–L gap	4.07	2.35	4.8	3.09	3.86	2.36	3.28	1.97
Δ*E*	0.0	0.0	12.2	5.7	14.9	6.9	20.0	17.6
⟨*S*^2^⟩	2.31	2.30	0.00	0.00	0.00	0.00	2.19	2.13

The next two C_12_H_12_Fe structures in terms of energy are singlet structures having favorable 18-electron iron configurations similar to ferrocene. Structure **Fe2–57S**, lying 12.2 kcal/mol (B3PW91) or 5.7 kcal/mol (M06-L) in energy above **Fe1–66T**, is the singlet mixed sandwich (η^5^-C_5_H_5_)Fe(η^5^-C_7_H_7_) having a planar pentahapto cyclopentadienyl ring but a nonplanar pentahapto cycloheptatrienyl ring with one uncomplexed C=C bond of length 1.336 Å(Figure 5 and Table 5). In this structure, the η^5^-C_7_H_7_ ring functioning as the pentahapto ligand is similar to the open chain pentadienyl ligand in the M(η^5^-C_5_H_7_)(CO)_3_(M = Mn or Re) structure.^33^ Of similar energy at 14.9 kcal/mol (B3PW91) or 6.9 kcal/mol (M06-L) above **Fe1–66T** is the singlet dibenzeneiron structure (η^6^-C_6_H_6_)Fe(η^4^-C_6_H_6_) **(Fe3–66S)** having one planar hexahapto η^6^-C_6_H_6_ benzene ring and one nonplanar tetrahapto η^4^-C_6_H_6_ benzene ring with an uncomplexed C=C bond of length ∼1.35 Å. The coordination environment of structure **Fe3–66S** is similar to that in the experimentally known structure (η^4^-C_4_H_6_)Fe(CO)_3_^34,35^ in which both C=C bonds of butadiene are coordinated to the central iron because of the isoelectronic character of the Fe(CO)_3_ and Fe(η^6^-C_6_H_6_) moieties. In both **Fe2–57S** and **Fe3–66S** ten carbon atoms in the two rings are bonded to the central iron atom thereby giving it the favored 18-electron configuration.

The triplet mixed sandwich structure (η^5^-C_5_H_5_)Fe(η^4^-C_7_H_7_) **(Fe4–57T)**, lying 20.0 kcal/mol (B3PW91) or 17.6 kcal/mol (M06-L) above the lowest-energy structure **Fe1–66T**, has a planar cyclopentadienyl ligand but a nonplanar cycloheptatrienyl ring (Figure 5 and Table 5). The lowest-energy (C_4_H_4_)Fe(C_8_H_8_) structure was predicted to be the singlet (η^4^-C_4_H_4_)Fe(η^6^-C_8_H_8_) structure **Fe7–48S** at the very high energy of 72.9 kcal/mol(B3PW91) or 58.6 kcal/mol(M06-L) above **Fe1–66T**.

### 3.6 C_12_H_12_Co Sandwiches

**Figure 6:**
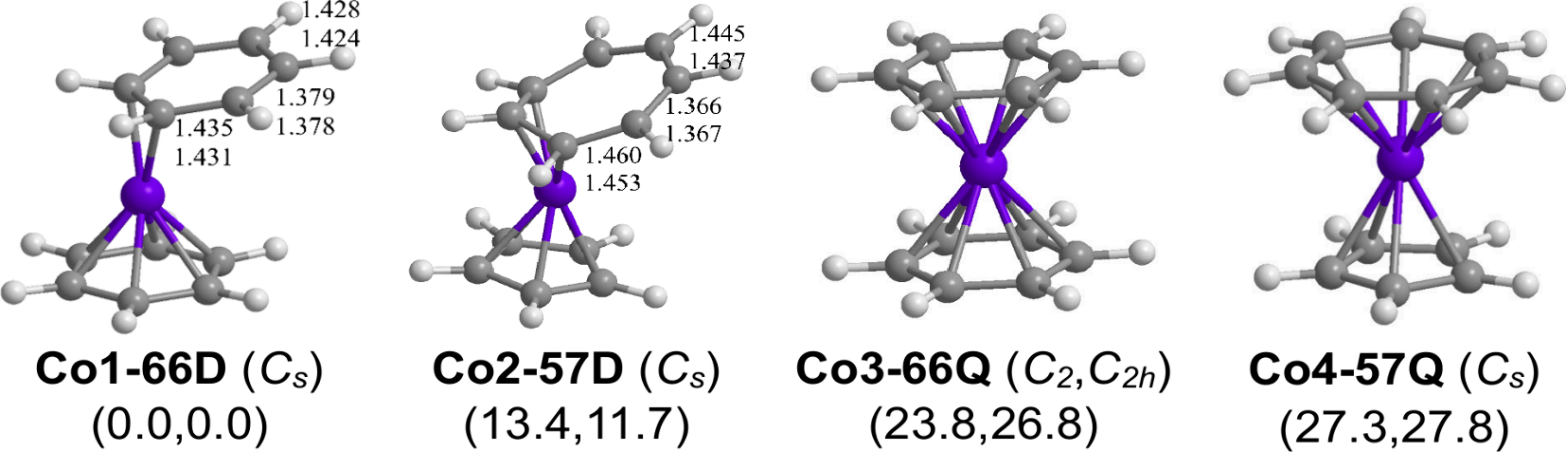
Optimized (C_*n*_H_*n*_)Co(C_*m*_H_*m*_) (*n* = 6 or 5; *m* = 6 or 7; *n* + *m* = 12) structures.

The lowest-energy (C_*n*_H_*n*_)Co(C_*m*_H_*m*_) (*n* = 6, 5, or 4; *m* = 6, 7, or 8; *n* + *m* = 12) structure is the doublet dibenzenecobalt structure having one hexahapto η^6^-C_6_H_6_ benzene ring and one dihapto η^2^-C_6_H_6_ benzene ring with two uncomplexed C=C bonds of length ∼1.38 Å (Figure 6 and Table 6). This bonding mode of the two benzene rings in **Co1–66D** leads to the dihapto η^2^-C_6_H_6_ ring being tilted with respect to the hexahapto η^6^-C_6_H_6_ ring. The magnetic moment indicating one unpaired electron coupled with the observation of a dipole moment for the experimentally known very unstable bis(hexamethylbenzene)-cobalt^18^ is consistent with a hexahapto-dihapto (η^6^-Me_6_C_6_)Co(η^2^-Me_6_C_6_) structure completely analogous to the unsubstituted (η^6^-C_6_H_6_)Co(η^2^-C_6_H_6_) structure **Co1–66D.**

**Table 6:** Bond Distances (in angstroms), Relative Energies (Δ*E* in kilocalories per mole), Spin Expectation Values ⟨*S*^2^⟩, and HOMO–LUMO Gaps (H–L gaps, in electronvolts) for the (C_*n*_H_*n*_)Co(C_*m*_H_*m*_) (*n* = 6 or 5; *m* = 6 or 7; *n* + *m* = 12) Structures

	**Co1–66D** (*C*_*s*_)		**Co2–57D** (*C*_*s*_)		**Co3–66Q** (*C*_2_, *C*_2*h*_)		**Co4–57Q** (*C*_*s*_)	
	B3PW91	M06-L	B3PW91	M06-L	B3PW91	M06-L	B3PW91	M06-L
M–C_*n*_H_*n*_	2.49	2.47	2.15	2.11	2.28	2.25	2.26	2.24
M–C_*m*_H_*m*_			2.66	2.65			2.30	2.28
H–L gap	4.53	3.21	3.57	2.43	1.05	0.26	1.94	0.71
Δ*E*	0.0	0.0	13.4	11.7	23.8	26.8	27.3	27.8
⟨*S*^2^⟩	0.85	0.85	0.85	0.81	3.80	3.82	3.90	3.89

The doublet mixed sandwich structure (η^5^-C_5_H_5_)Co(η^3^-C_7_H_7_) **(Co2–57D)**, lying 13.4 kcal/mol (B3PW91) or 11.7 kcal/mol (M06-L) above **Co1–66D**, has a planar pentahapto η^5^-C_5_H_5_ cyclopentadienyl ring and a nonplanar trihapto η^3^-C_7_H_7_ ring with two uncomplexed C=C bonds of length ∼1.37 Å. Both **Co1–66D** and **Co2–57D** have 17-electron configurations for their central cobalt atoms.

### 3.7 C_12_H_12_Ni Sandwiches

**Figure 7:**
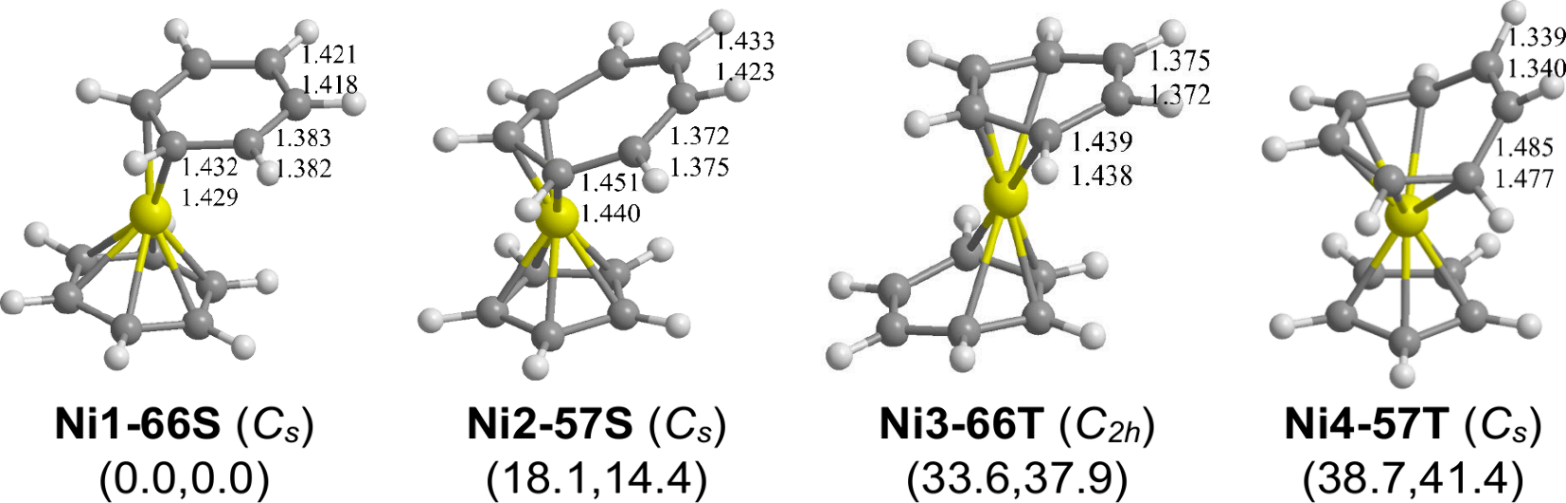
Optimized (C_*n*_H_*n*_)Ni(C_*m*_H_*m*_) (*n* = 6 or 5; *m* = 6 or 7; *n* + *m* = 12) structures.

The lowest-energy (C_*n*_H_*n*_)Ni(C_*m*_H_*m*_) (*n* = 6, 5, or 4; *m* = 6, 7, or 8; *n* + *m* = 12) structure by a margin of 14 kcal/mol is the dibenzenenickel complex (η^6^-C_6_H_6_)Ni(η^2^-C_6_H_6_) **(Ni1–66S)** having one hexahapto η^6^-C_6_H_6_ benzene ligand and one dihapto η^2^-C_6_H_6_ ligand with two uncomplexed C=C bonds of length ∼1.38 Å in the latter ring (Figure 7 and Table 7). This arrangement of benzene ligands in **Ni1–66S** gives the central nickel atom the favored 18-electron configuration. Structure **Ni1–66S** is very similar to the lowest-energy C_12_H_12_Co structure **Co1–66D** (Figure 6 and Table 6) as well as the likely structure of the experimentally known bis(hexamethylbenzene)-cobalt.^18^

**Table 7:** Bond Distances (in angstroms), Relative Energies (Δ*E* in kilocalories per mole), Spin Expectation Values ⟨*S*^2^⟩, and HOMO–LUMO Gaps (H–L gaps, in electronvolts) for the (C_*n*_H_*n*_)Ni(C_*m*_H_*m*_) (*n* = 6 or 5; *m* = 6 or 7; *n* + *m* = 12) Structures

	**Ni1–66S** (*C*_*s*_)		**Ni2–57S** (*C*_*s*_)		**Ni3–66T** (*C*_2*h*_)		**Ni4–57T** (*C*_*s*_)	
	B3PW91	M06-L	B3PW91	M06-L	B3PW91	M06-L	B3PW91	M06-L
M–C_*n*_H_*n*_	2.48	2.45	2.14	2.11	2.46	2.41	2.23	2.25
M–C_*m*_H_*m*_			2.69	2.68			2.45	2.43
H–L gap	4.13	2.49	3.45	2.17	1.77	1.01	3.00	1.57
Δ*E*	0.0	0.0	18.1	14.4	33.6	37.9	38.7	41.4
⟨*S*^2^⟩	0.00	0.00	0.00	0.00	2.01	2.01	2.02	2.02

The next higher energy C_12_H_12_Ni structure, namely the singlet mixed sandwich (η^5^-C_5_H_5_)Ni(η^3^-C_7_H_7_) **(Ni2–57S)** lying 18.1 kcal/mol (B3PW91) or 14.4 kcal/mol (M06-L) above **Ni1–66S**, has a planar pentahapto η^5^-C_5_H_5_ cyclopentadienyl ring and a nonplanar trihapto η^3^-C_7_H_7_ cycloheptatrienyl ring with two uncomplexed C=C bonds of lengths ∼1.37 Å. This bonding arrangement of the two rings in **Ni2–57S** gives the central nickel atom the favored 18-electron configuration.

The next two C_12_H_12_Ni structures in terms of energy are the triplet dibenzenenickel structure (η^4^-C_6_H_6_)Ni(η^4^-C_6_H_6_) **(Ni3–66T)**, lying 33.6 kcal/mol (B3PW91) or 37.9 kcal/mol (M06-L) and the triplet mixed sandwich structure (η^5^-C_5_H_5_)Ni(η^5^-C_7_H_7_) **(Ni4–57T)**, lying 38.7 kcal/mol (B3PW91) or 41.4 kcal/mol (M06-L) above the lowest-energy structure **Ni1–66S** (Figure 7 and Table 7). For **Ni3–66T**, both benzene rings are predicted to be tetrahapto η^4^-C_6_H_6_ ligands apparently giving the central nickel atom an 18-electron configuration despite the triplet spin state. Both rings in the mixed sandwich compound **Ni4–57T** are pentahapto ligands similar to the arrangement of the ligands in the iron sandwich (η^5^-C_5_H_5_)Fe(η^5^-C_7_H_7_) **Fe2–57S**. The central nickel atom in **Ni4–57T** thus has a high-spin 20-electron πconfiguration similar to the nickel atom in nickelocene (η^5^-C_5_H_5_)_2_Ni.^30−32^

### 3.8 (C_4_H_4_)M(C_8_H_8_) Sandwiches

The cyclobutadiene-cyclooctetraene sandwich structures (C_4_H_4_)M(C_8_H_8_) of the first row transition metals have consistently very high energies ranging from 61.7 kcal/mol for (C_4_H_4_)Ti(C_8_H_8_) to 85.2 kcal/mol for (C_4_H_4_)Cr(C_8_H_8_) relative to their lowest-energy (C_5_H_5_)M(C_7_H_7_) or (C_6_H_6_)_2_M isomers. However, the (C_4_H_4_)M(C_8_H_8_) sandwiches and their substitution products could be kinetically stabilized species because of very large activation energies for breaking ring C–C bonds in the C_*n*_H_*n*_ ligands in order to change the sizes of the carbocyclic rings. Thus, the (C_4_H_4_)M(C_8_H_8_) sandwiches could be species stable under ambient conditions if synthesized from suitable reagents containing preformed C_4_H_4_ and C_8_H_8_ rings. For example, a methyl-substituted nickel derivative (Me_4_C_4_)Ni(C_8_H_8_) might be accessible from a reaction starting with the experimentally known tetramethylcyclobutadiene derivative^36,37^ [(η^4^-Me_4_C_4_)NiCl_2_]_2_ with a source of the 10 π-electron aromatic cyclooctatetraene dianion. For this reason we have included a more detailed investigation of the (C_4_H_4_)M(C_8_H_8_) structures in our study despite their high energies relative to isomeric (C_5_H_5_)M(C_7_H_7_) and (C_6_H_6_)_2_M derivatives.

**Figure 8:**
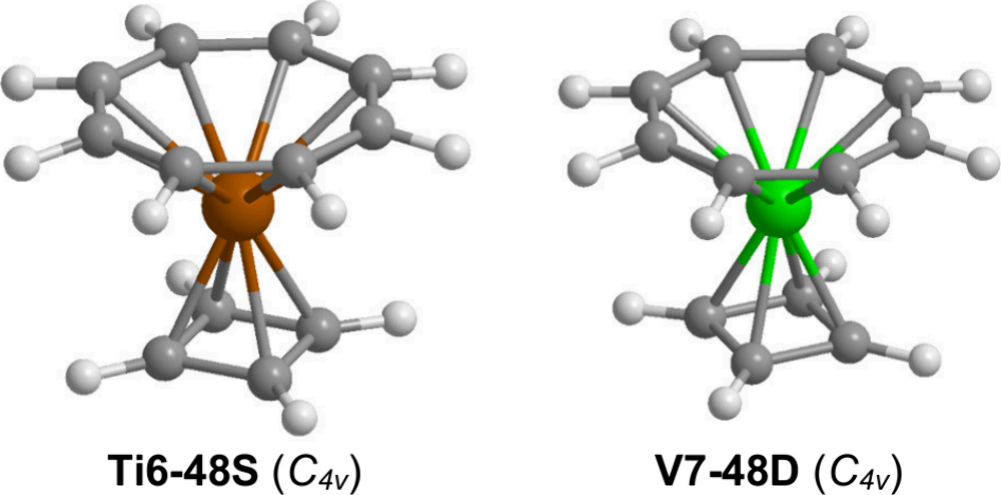
Optimized (C_4_H_4_)M(C_8_H_8_) (M = Ti and V) structures.

The lowest-energy structures of the early transition metal (C_4_H_4_)M(C_8_H_8_) (M = Ti and V) systems are both low spin structures, namely **Ti6–48S** and **V7–48D** with *C*_4*v*_ symmetry (Figure 8 and Table 8). The singlet (η^4^-C_4_H_4_)Ti(η^8^-C_8_H_8_) (**Ti6–48S**) and the doublet (η^4^-C_4_H_4_)V(η^8^-C_8_H_8_) (**V7–48D**) structures incorporate a planar η^8^-C_8_H_8_ ring and a planar η^4^-C_4_H_4_ ring having all 12 carbon atoms of the two rings within bonding distance of the metals (Figure 8 and Table 8). This leads to 16- and 17-electron configurations for the metal atoms in the sandwiches **Ti6–48S** and **V7–48D** corresponding to their singlet and doublet spin states, respectively. Both structures are essentially true minima with only very small imaginary vibrational frequencies below 20 cm^–1^. The average Ti–C bond distances for **Ti6–48S** are shorter for the C_4_H_4_ ligand (2.20 Å by B3PW91 or 2.18 Å by M06-L) than for the C_8_H_8_ ligand (2.32 Å by B3PW91 or 2.31 Å by M06-L). Furthermore, the average V–C bond distances for **V7–48D** are shorter for the C_4_H_4_ ligand (2.15 Å by B3PW91 or 2.14 Å by M06-L) than for the C_8_H_8_ ligand (2.29 Å by B3PW91 or 2.29 Å by M06-L).

**Table 8:** Bond Distances (in angstroms), Spin Expectation Values ⟨*S*^2^⟩, and HOMO–LUMO Gaps (H–L gaps, in electronvolts) for the Doublet (C_4_H_4_)M(C_8_H_8_) (M = Ti and V) Structures

	**Ti6–48S** (*C*_4*v*_)		**V7–48D** (*C*_4*v*_)	
	B3PW91	M06-L	B3PW91	M06-L
M–C_4_H_4_	2.20	2.18	2.15	2.14
M–C_8_H_8_	2.32	2.31	2.29	2.29
H–L gap	3.97	2.48	3.67	1.40
–*E*	1313.76443	1313.90916	1408.27851	1408.41401
⟨*S*^2^⟩	0.00	0.00	0.80	0.81

**Figure 9:**
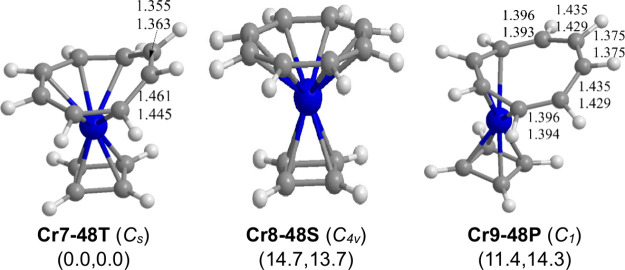
Optimized low-energy (C_4_H_4_)Cr(C_8_H_8_) structures.

For the analogous chromium system, the singlet sandwich structure (η^4^-C_4_H_4_)Cr(η^8^-C_8_H_8_) **(Cr8–48S)** with all of the carbon atoms from the C_4_H_4_ and C_8_H_8_ rings complexed to the central chromium atom is not the lowest-energy (C_4_H_4_)Cr(C_8_H_8_) structure despite the favorable 18-electron configuration for its chromium atom (Figure 9 and Table 9). Thus, **Cr8–48S** is predicted to lie 14.7 kcal/mol (B3PW91) or 13.7 kcal/mol (M06-L) higher in energy than the triplet (η^4^-C_4_H_4_)Cr(η^6^-C_8_H_8_) structure **Cr7–48T** with a 16-electron configuration containing a hexahapto η^6^-C_8_H_8_ ring. A quintet (η^4^-C_4_H_4_)Cr(η^4^-C_8_H_8_) structure **Cr9–48P**, containing a tetrahapto η^4^-C_8_H_8_ ring, is predicted to lie 11.4 kcal/mol (B3PW91) or 14.3 kcal/mol (M06-L) above its triplet isomer **Cr7–48T** similar to the energy of the singlet **Cr8–48S**. This suggests some difficulty of having all eight carbon atoms of a planar C_8_H_8_ ring within bonding distance of a chromium atom.

**Table 9:** Bond Distances (in angstroms), Relative Energies (Δ*E* in kilocalories per mole), Spin Expectation Values ⟨*S*^2^⟩, and HOMO–LUMO Gaps (H–L gaps, in electronvolts) for the (C_4_H_4_)Cr(C_8_H_8_) Structures

	**Cr7–48T** (*C*_*s*_)		**Cr8–48S** (*C*_4*v*_)		**Cr9–48P** (*C*_1_)	
	B3PW91	M06-L	B3PW91	M06-L	B3PW91	M06-L
M–C_4_H_4_	2.11	2.10	2.10	2.08	2.12	2.10
M–C_8_H_8_	2.43	2.41	2.27	2.26	2.82	2.80
H–L gap	2.88	1.37	2.60	0.43	3.50	2.30
Δ*E*	0.0	0.0	14.7	13.7	11.4	14.3
⟨*S*^2^⟩	2.34	2.32	0.00	0.00	6.18	6.16

**Figure 10:**
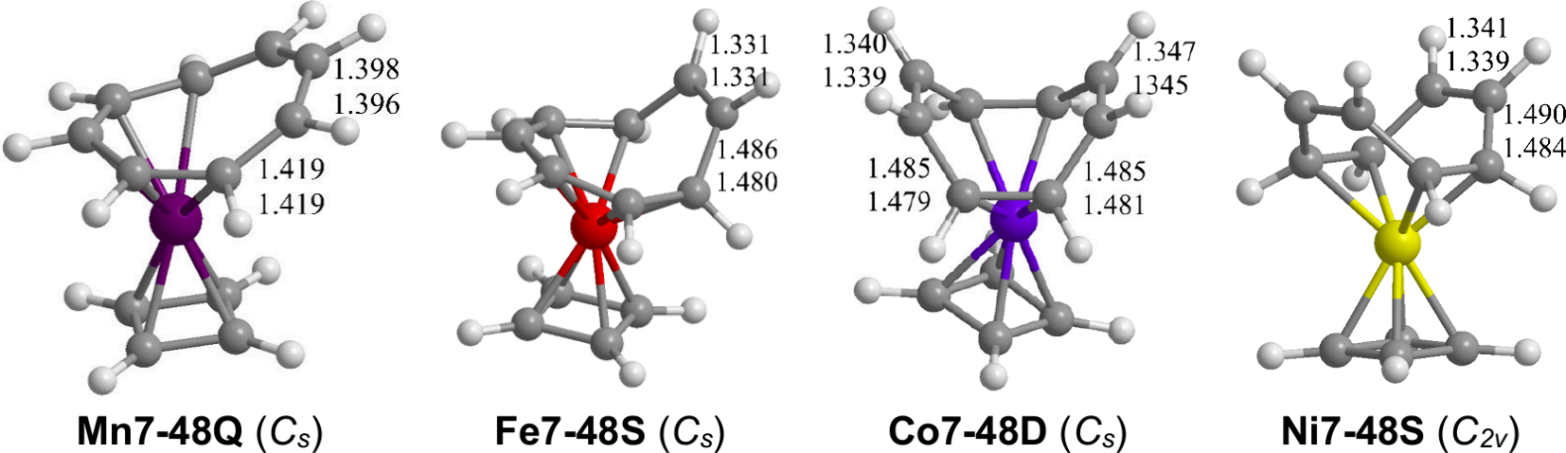
Optimized lowest-energy (C_4_H_4_)M(C_8_H_8_) (M = Mn–Ni) structures.

The lowest-energy structure for the C_4_H_4_MnC_8_H_8_ system is the quartet (η^4^-C_4_H_4_)Mn(η^5^-C_8_H_8_) (**Mn7–48Q**) structure containing a tetrahapto η^4^-C_4_H_4_ ring and a pentahapto η^5^-C_8_H_8_ ring, leaving an allylic unit not bonding to the manganese atom (Figure 10 and Table 10). The nonbonding Mn–C distances in **Mn7–48Q** are 3.412 Å (B3PW91) or 3.357 Å (M06-L) and 3.101 Å (B3PW91) or 3.050 Å (M06-L).

**Table 10:** Bond Distances (in angstroms), Total Energies (*E* in hartrees), Spin Expectation Values ⟨*S*^2^⟩, and HOMO–LUMO Gaps (H–L gaps, in electronvolts) for the (C_4_H_4_)M(C_8_H_8_) (M = Mn–Ni) Structures

	Mn7-48Q (*C*_*s*_)		Fe7-48S (*C_s_*)	
	B3PW91	M06-L	B3PW91	M06-L
M–C_4_H_4_	2.10	2.08	2.01	2.00
M–C_8_H_8_	2.60	2.57	2.32	2.31
H–L gap	2.85	1.86	4.66	2.72
–*E*	1615.23444	1615.35616	1727.96034	1728.07879
⟨*S*^2^⟩	4.08	4.03	0.00	0.00

	**Co7–48D** (*C*_*s*_)		**Ni7–48S** (*C*_*2v*_)	
	B3PW91	M06-L	B3PW91	M06-L
M–C_4_H_4_	2.01	2.00	2.04	2.02
M–C_8_H_8_	2.46	2.46	2.20	2.45
H–L gap	3.59	2.48	3.59	2.32
–*E*	1847.00156	1847.10572	1972.56751	1972.65578
⟨*S*^2^⟩	0.77	0.77	0.00	0.00

Low-spin structures are found to be the lowest-energy structures for the (C_4_H_4_)M(C_8_H_8_) (M = Fe, Co, or Ni) complexes of the late first-row transition metals, namely the singlet **Fe7–48S**, the doublet **Co7–48D**, and the singlet **Ni7–48S** (Figure 10 and Table 10). The singlet (η^4^-C_4_H_4_)Fe(η^6^-C_8_H_8_) (**Fe7–48S**) has a tetrahapto η^4^-C_4_H_4_ ring and a hexahapto η^6^-C_8_H_8_ ring, corresponding to the favored 18-electron configuration for the iron atom. The cobalt atom in the doublet (η^4^-C_4_H_4_)Co(η^2,2^-C_8_H_8_) **Co7–48D**) structure having an η^4^-C_4_H_4_ ring and an η^2,2^-C_8_H_8_ ring has a 17-electron configuration. The coordination modes of the C_8_H_8_ and C_4_H_4_ rings in the singlet (η^4^-C_4_H_4_)Ni(η^2,2^-C_8_H_8_) (**Ni7–48S**) structure are the same as those in the cobalt structure **Co7–48D** thereby leading to the favored 18-electron configuration for the nickel atom. Nonadjacent C=C bonds in the η^2,2^-C_8_H_8_ ring are used for complexation with the central metal atom in **Co7–48D** and **Ni7–48S.** The two remaining uncomplexed C=C bonds within the η^2,2^-C_8_H_8_ ring for **Co7–48D** and **Ni7–48S** have lengths of ∼1.34 Å.

### 3.9 Frontier Molecular Orbital Analysis of Sandwich Compounds with Two Planar Rings

**Figure 11:**
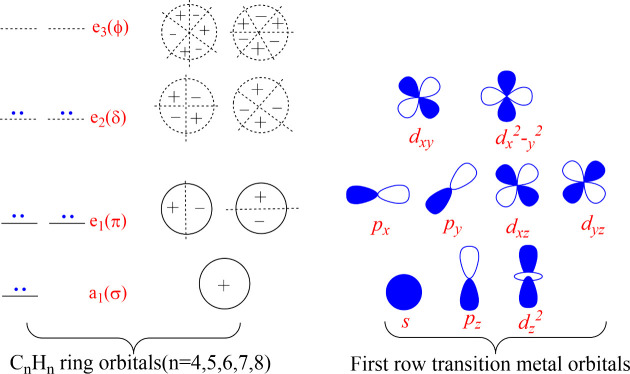
Symmetries of the frontier orbitals for the η^*n*^-C_*n*_H_*n*_ rings and the transition metal atoms.

**Figure 12:**
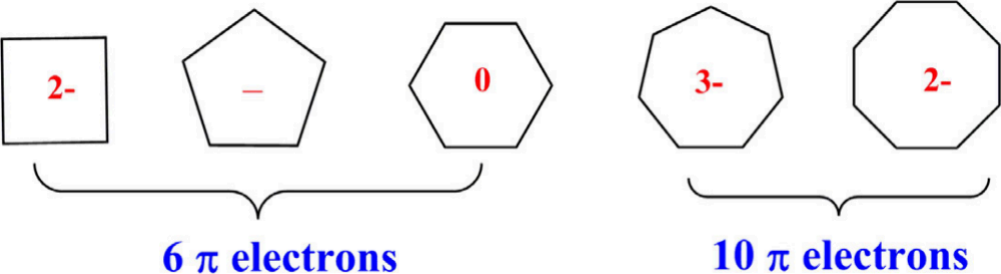
Number of π electrons for the C_*n*_H_*n*_ (*n* = 4–8) anions.

According to the (4*n* + 2) π-electron rules for planar carbocyclic rings and related species, bonding of planar η^4^-C_4_H_4_^2–^ and η^5^-C_5_H_5_^–^ anions and neutral η^6^-C_6_H_6_ with 6 π-electrons to a metal atom implies a metal–ring bond with three components using the a_1_ and e_1_ ring orbitals to form one σ and two π bonds with the metal p_*z*_ and d_*xz*_/d_*yz*_ orbitals, respectively. Similarly bonding of planar η^7^-C_7_H_7_^3–^ and η^8^-C_8_H_8_^2–^ rings with 10 π-electrons implies a metal–ring bond with five components using the ring a_1_, e_1_, and e_2_ orbitals to form one σ, two π, and two δ bonds with the metal p_*z*_, d_*xz*_/d_*yz*_, and d_*x*2–*y*2_/d_*xy*_ orbitals, respectively. The metal *dz*^2^ orbitals are not involved in the metal-ring bonding (Figures 11 and 12).

**Figure 13:**
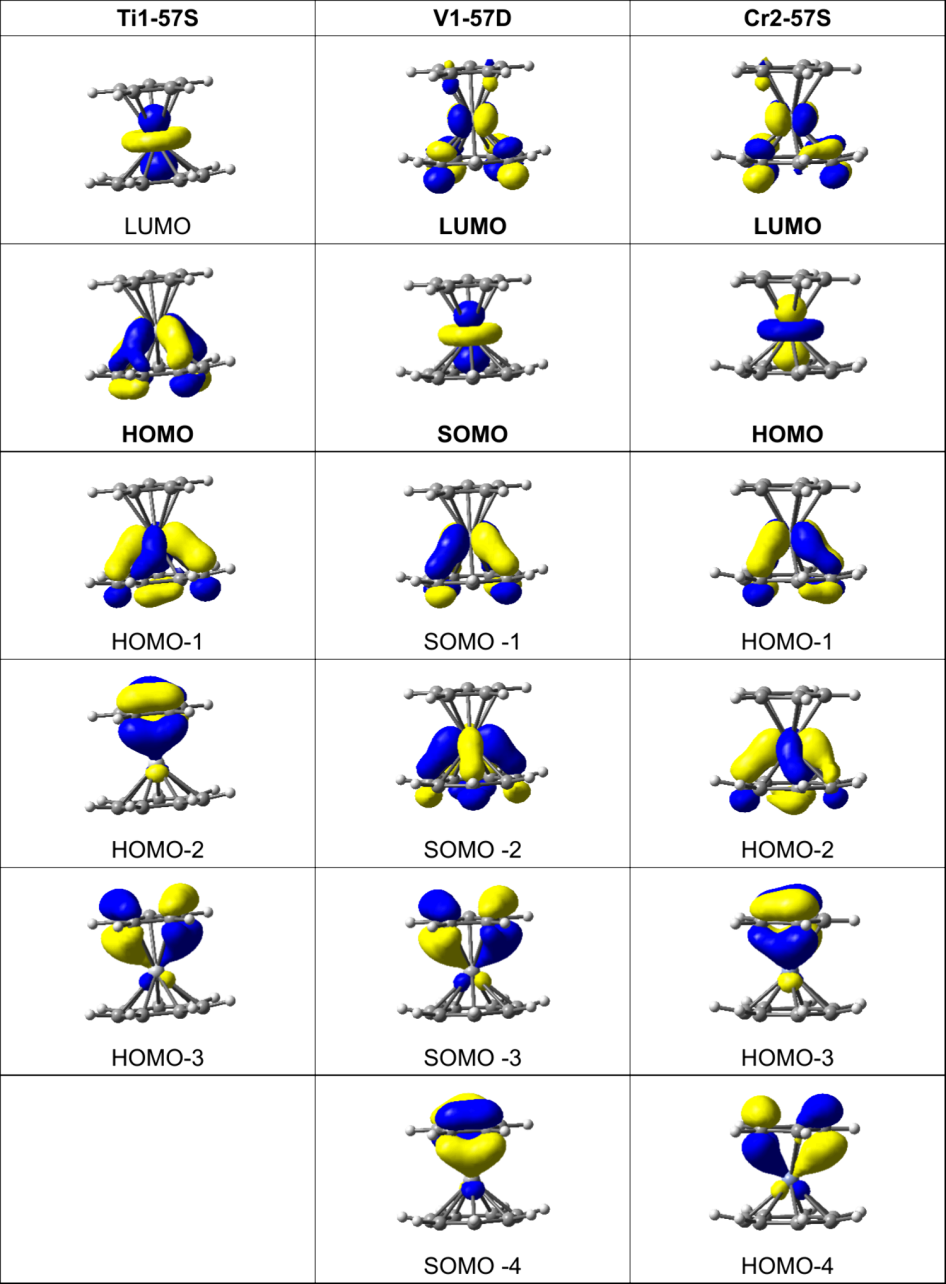
Frontier molecular orbitals of the lowest-energy (η^5^-C_5_H_5_)M(η^7^-C_7_H_7_) complexes (M = Ti, V, or Cr).

Consider first the cyclopentadienyl-cycloheptatrienyl metal sandwich compounds (η^5^-C_5_H_5_)M(η^7^-C_7_H_7_) (M = Ti, V, or Cr) with planar rings (Figure 13)^38^. The nonbonding *dz*^2^ orbitals are empty in the titanium derivative (LUMO in Figure 13), singly occupied in the vanadium derivative (SOMO in Figure 13), and doubly occupied in the chromium derivative (HOMO in Figure 13). The two molecular orbitals immediately below that of the *dz*^2^ orbitals correspond to the δ bonding of the e_2_ orbitals of the C_7_H_7_ ring to the metal d_*x*2–*y*2_/d_*xy*_ orbitals. The next two molecular orbitals immediately below the δ-bonding orbitals of the metal to the seven-membered C_7_H_7_ ring are the π-bonding orbitals of the five-membered C_5_H_5_ ring e_1_ orbitals to the metal d_*xz*_/d_*yz*_ orbitals.

**Figure 14:**
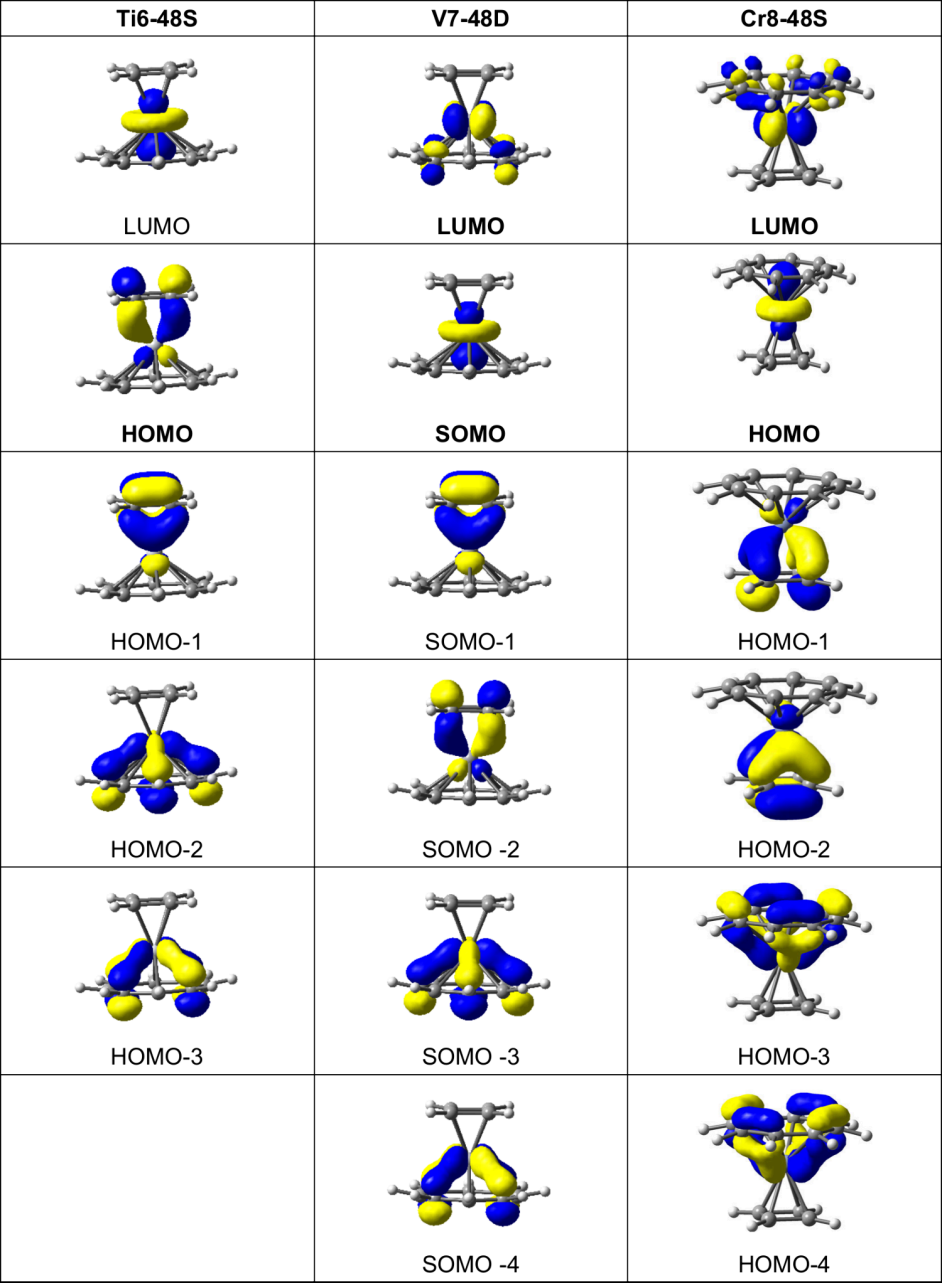
Frontier molecular orbitals of the lowest-energy (η^4^-C_4_H_4_)M(η^8^-C_8_H_8_) complexes (M = Ti, V, or Cr).

The pattern of the frontier molecular orbitals of the (η^4^-C_4_H_4_)M(η^8^-C_8_H_8_) sandwiches (M = Ti, V, or Cr) with planar four- and eight-membered carbocyclic rings (Figure 14) is related to that of those of the (η^5^-C_5_H_5_)M(C_7_H_7_) with planar five- and seven-membered carbocyclic rings (Figure 13) except for a reversal of the sequence of molecular orbitals corresponding to π-bonding of the e_1_ orbitals of the smaller ring to those corresponding to δ-bonding of the e_2_ orbitals of the larger ring to the central metal atom. For example, for **Ti6–48S** the LUMO is the empty titanium *dz*^2^ orbital in accord with the 16-electron configuration of the central titanium atom. The HOMO and HOMO–1 corresponding to the π-bonding of the e_1_ orbitals of the four-membered η^4^-C_4_H_4_ ring to the d_*xz*_/d_*yz*_ titanium orbitals (Figure 14). Next come HOMO–2 and HOMO–3 corresponding to the δ-bonding of the e_2_ orbitals of the eight-membered η^8^-C_8_H_8_ ring to the titanium d_*x*2–*y*2_/d_*xy*_ orbitals. This differs from the relative energies of the frontier molecular orbitals in the isomeric singlet **Ti1–57S** in which those corresponding to the δ-bonding of the e_2_ orbitals of the eight-membered η^8^-C_8_H_8_ ring to the titanium d_*x*2–*y*2_/d_*xy*_ orbitals are HOMO and HOMO–1 lying in energy immediately below that of the empty LUMO corresponding to the titanium *dz*^2^ orbitals. The frontier molecular orbital pattern of the corresponding vanadium and chromium (η^4^-C_4_H_4_)M(η^8^-C_8_H_8_) derivatives are similar to those of the titanium derivative.

**Figure 15:**
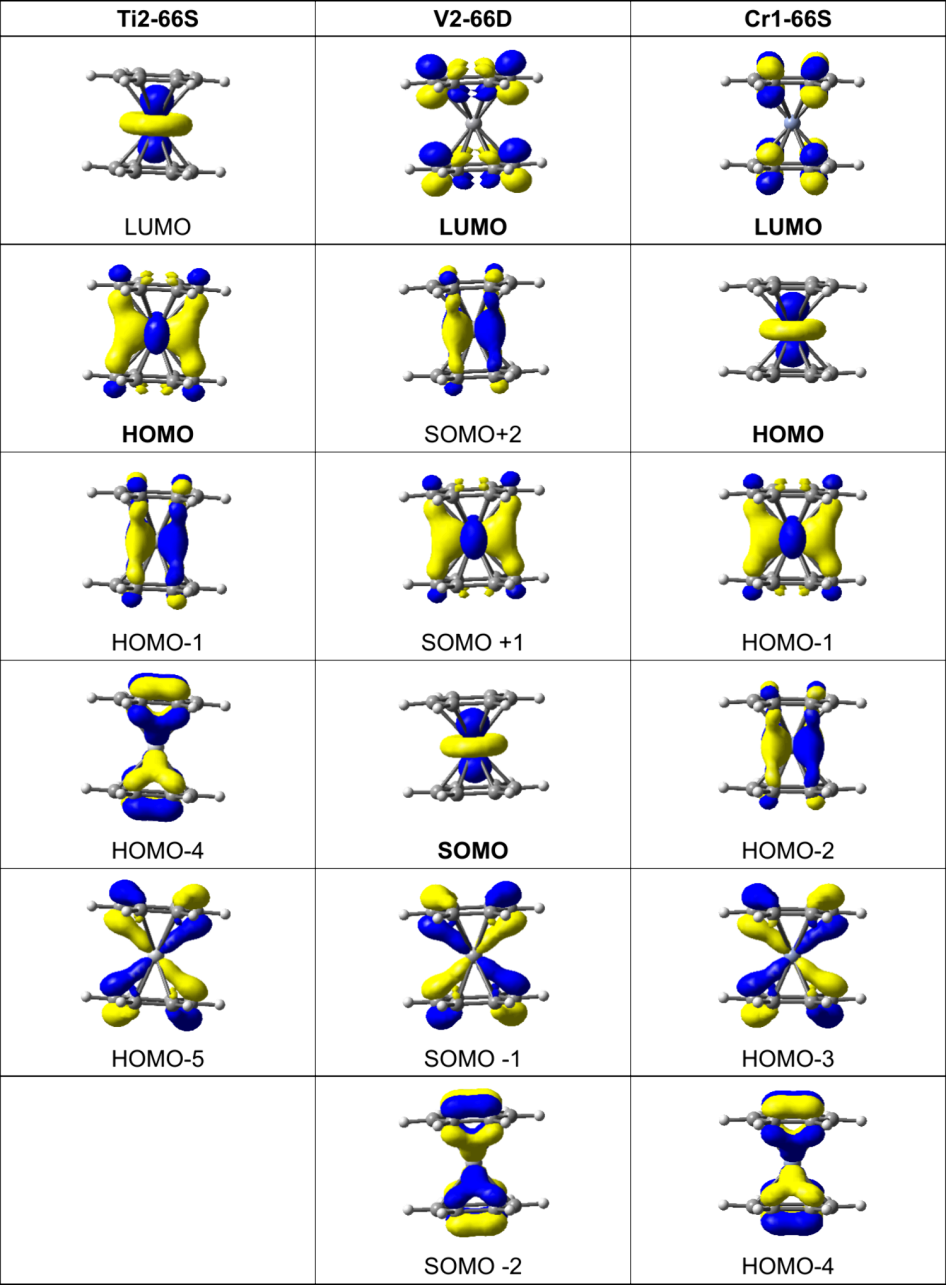
Frontier molecular orbitals of the lowest-energy dibenzenemetal complexes (η^6^-C_6_H_6_)_2_M (M = Ti, V, or Cr).

The frontier molecular orbital pattern for the dibenzene sandwich complexes (η^6^-C_6_H_6_)_2_M (M = Ti, V, or Cr) is different from that of the isomeric (η^5^-C_5_H_5_)M(η^7^-C_7_H_7_) and (η^4^-C_4_H_4_)M(η^8^-C_8_H_8_) derivatives since a neutral benzene ring has only filled a_1_ and e_1_ molecular orbitals for bonding to the metal atom (Figure 15). For this reason, the metal d_*x*2–*y*2_/d_*xy*_ orbitals as well as the metal *dz*^2^ orbitals do not participate in forward ring → metal bonding. However, metal → ring δ back-bonding of the filled metal d_*x*2–*y*2_/d_*xy*_ orbitals into empty ring e_2_ molecular orbitals is possible. The two highest energy filled molecular orbitals correspond to back-bonding of this type. For example, in dibenzenechromium **Cr1–66S** the HOMO is the filled nonbonding chromium *dz*^2^ orbitals followed by HOMO–1 and HOMO–2 corresponding to metal d_*x*2–*y*2_/d_*xy*_ →ring e_2_ δ back-bonding. The next two frontier molecular orbitals of **Cr1–66S**, namely HOMO–3 and HOMO–4, correspond to ring e_1_ → metal d_*xz*_/d_*yz*_ forward π-bonding. Related frontier molecular orbital patterns are observed for the titanium and vanadium derivatives.

## 4 Summary

Ring size effects on the geometries and electronic structures were investigated for the (C_*n*_H_*n*_)M(C_*m*_H_*m*_) (*n* = 4, 5, or 6; *m* = 8, 7, or 6; *m* + *n* = 12; M = Ti–Ni) systems using density functional theory. For all of the first row transition metals the structures of the type (C_4_H_4_)M(C_8_H_8_) with the metal sandwiched between four and eight-membered rings were found to be much higher energy structures by margins of at least ∼60 kcal/mol over their lowest-energy (C_5_H_5_)M(C_7_H_7_) and (C_6_H_6_)_2_M isomers.

The lowest-energy C_12_H_12_M structures for the early transition metals titanium, vanadium, and chromium are the experimentally known singlet (η^5^-C_5_H_5_)Ti(η^7^-C_7_H_7_), doublet (η^5^-C_5_H_5_)V(η^7^-C_7_H_7_), and singlet (η^6^-C_6_H_6_)_2_Cr. The likewise experimentally known singlet (η^6^-C_6_H_6_)_2_Ti, doublet (η^6^-C_6_H_6_)_2_V, and singlet (η^5^-C_5_H_5_)Cr(η^7^-C_7_H_7_) as the second lowest-energy structures with only a small difference of ∼3 kcal/mol between the two vanadium structures. For the later transition metals dibenzenemetal complexes are the lowest-energy species. Thus for manganese the lowest-energy C_12_H_12_M structures are quartet (η^6^-C_6_H_6_)_2_Mn structure and the only slightly higher energy very similar doublet structure in which each benzene ring is a fully bonded planar hexahapto ligand leading to a 19-electron manganese configuration. The lowest-energy C_12_H_12_Fe structure is the triplet sandwich (η^6^-C_6_H_6_)_2_Fe related to the experimentally known (η^6^-Me_6_C_6_)_2_Fe with a 20-electron iron configuration. The lowest-energy C_12_H_12_M (M = Co or Ni) structures have one fully bonded hexahapto η^6^-C_6_H_6_ ring and one essentially planar dihapto η^2^-C_6_H_6_ ring with two uncomplexed C=C bonds to give the central metal atom a 17-electron configuration for cobalt and an 18-electron configuration for nickel. The lowest-energy (C_5_H_5_)M(C_7_H_7_) structures for the later transition metals iron, cobalt, and nickel have partially bonded nonplanar C_7_H_7_ rings having one (for M = Fe) or two (for M = Co or Ni) uncomplexed C=C bonds to give the central metal atom a 17- or 18-electron configuration.

The patterns of the frontier molecular orbitals for the early transition metal derivatives (η^5^-C_5_H_5_)M(η^7^-C_7_H_7_) (M = Ti, V, or Cr) with planar carbocyclic rings are distinctly different from those for the isomeric dibenzene complexes (η^6^-C_6_H_6_)_2_M. This difference relates to the fact that the larger C_7_H_7_^3–^ and C_8_H_8_^2–^ rings as anionic ligands have 10 π-electrons that can be donated to the central metal atom through the five bond components σ + 2π + 2δ. However, the smaller rings C_4_H_4_^2–^, C_5_H_5_^–^, and C_6_H_6_ as anionic or neutral ligands have only 6 π-electrons and thus can bond to the central metal atom through only the three bond components σ + 2π.

## Supporting Information Available

All optimized (C_*n*_H_*n*_)M(C_*m*_H_*m*_) (*n* = 6, 5, or 4; *m* = 6, 7, or 8; *m* + *n* = 12; M = Ti–Ni) structures with the B3PW91 and M06-L method, in which the numbers in the first parentheses are the relative energies of all the (C_*n*_H_*n*_)M(C_*m*_H_*m*_) structures with different ring sizes and the numbers in the second parentheses are the relative energies for each (C_4_H_4_)M(C_8_H_8_) system (Figures S1–S7); harmonic vibrational frequencies (in inverse centimeters) and infrared intensities (in parentheses, in kilometers per mole) for the (C_*n*_H_*n*_)M(C_*m*_H_*m*_) (*n* = 6, 5, or 4; *m* = 6, 7, or 8; *m* + *n* = 12; M = Ti–Ni) structures calculated with the B3PW91 method and the M06-L method (Tables S8–S70); and the complete Gaussian09 reference^23^ (PDF)
Coordinates of structures (XYZ)

## Supplementary Material

om4c00210_si_001.pdf

om4c00210_si_002.xyz
