# Supplementary material for: Ring Size Effects on the Structures of Sandwich Compounds with a Stoichiometry of C12H12M (M = Ti–Ni)
Source: Organometallics. 2024 Nov 18;43(23):2981–94. doi: 10.1021/acs.organomet.4c00210 (PMC11632761; doi:10.1021/acs.organomet.4c00210)
Supplement: Supplementary file 1 — om4c00210_si_001.pdf [file om4c00210_si_001.pdf]

Supporting Information

Ring Size Effects on the Structures of Sandwich Compounds of Stoichiometry $C_{12}H_{12}M$ (M = Ti to Ni)

Huidong Li, [a,b]*Ruilin Lu,[a] Haoyu Chen,[a] Jinfeng Luo,[a] Qunchao Fan,[a]
R. Bruce King, [b]* and Henry F. Schaefer, III [b]

[a] *School of Science, Key Laboratory of High Performance Scientific Computation, Xihua University, Chengdu, China 610039*
[b]*Center for Computational Quantum Chemistry, University of Georgia, Athens, Georgia, USA 30602*

rbking@chem.uga.edu; huidongli@mail.xhu.edu.cn

Tables S1 to S7: All optimized $(C_nH_n)M(C_mH_m)$ (n=6,5,4;m=6,7,8;m+n=12; M=Ti-Ni) structures with the B3PW91 and M06-L method, the numbers in the first parenthesis are the relative energies of all the $(C_nH_n)M(C_mH_m)$ structures with different ring sizes, the numbers in the second parenthesis are the relative energies for each $(C_4H_4)M(C_8H_8)$ system.

Tables S8 to S70: Harmonic vibrational frequencies (in cm^{-1}) and infrared intensities (in parentheses, in km/mol) for the$(C_nH_n)M(C_mH_m)$ (n=6,5,4;m=6,7,8;m+n=12; M=Ti-Ni) structures calucated with the B3PW91 method and M06-L method.

Complete Gaussian09 reference (Reference 23).

Table S1. Optimized $(C_nH_n)Ti(C_mH_m)$ (n=6,5,4;m=6,7,8) structures

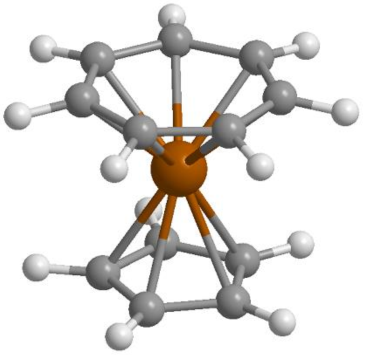<br>Ti1-57S(C_s)<br>(0.0,0.0)<br>(0.0,0.0)	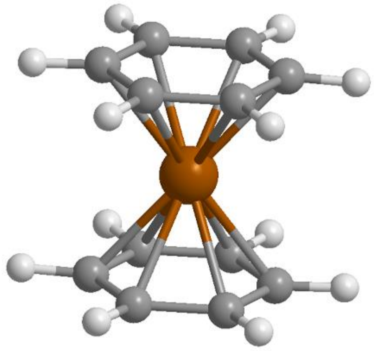<br>Ti2-66S(D_{6h})<br>(14.8,16.3)<br>(0.0,0.0)	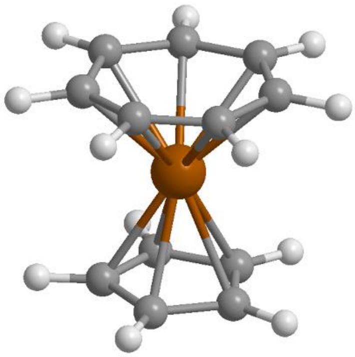<br>Ti3-57T(C_s)<br>(22.1,26.7)<br>(22.1,26.7)
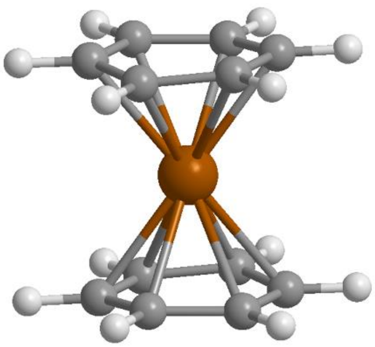<br>Ti4-66T(D_{2h})<br>(23.6,27.0)<br>(8.8,10.8)	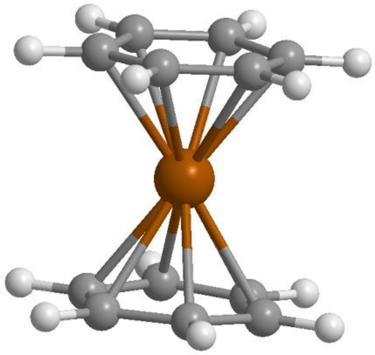<br>Ti5-66P(D_{2d})<br>(54.5,58.5)<br>(39.8,42.2)	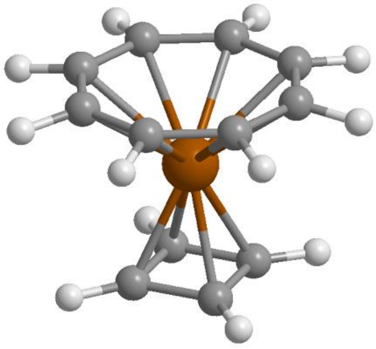<br>Ti6-48S(C_{4v})<br>(61.7,56.7)<br>(0.0,0.0)
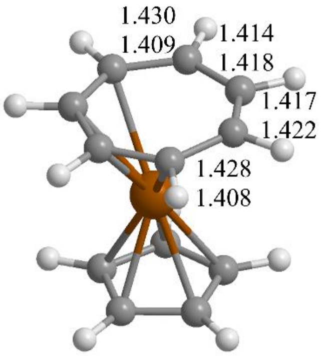<br>Ti7-57P(C_1)<br>(64.3,70.8)<br>(64.3,70.8)	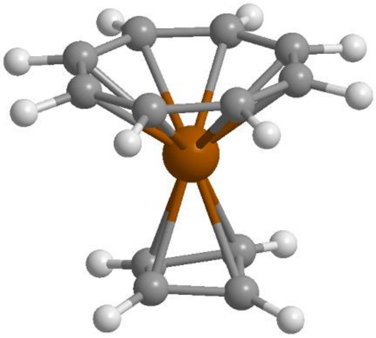<br>Ti8-48T(C_2)<br>(93.4,94.9)<br>(31.7,38.2)	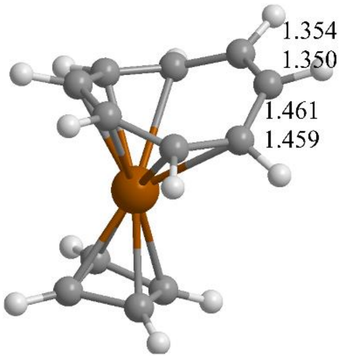<br>Ti9-48P(C_S)<br>(142.8,145.8)<br>(81.1,89.1)

Table S2. Optimized $(C_nH_n)V(C_mH_m)$ (n=6,5,4;m=6,7,8) structures

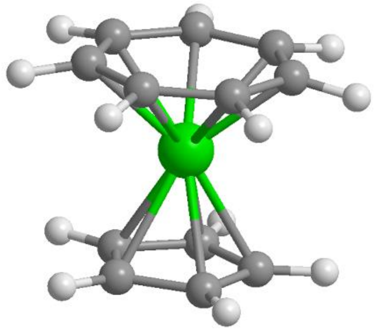<br>V1-57D(C_s)<br>(0.0,0.0)<br>(0.0,0.0)	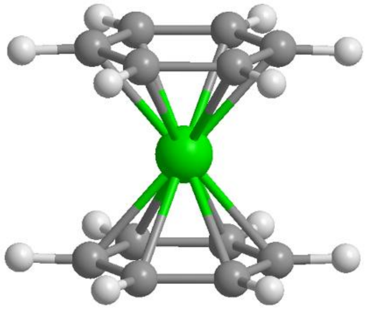<br>V2-66D(D_{6h})<br>(2.5,3.9)<br>(0.0,0.0)	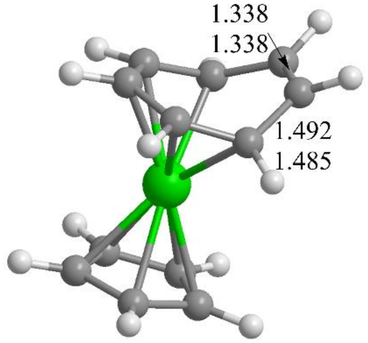<br>V3-57Q(C_s)<br>(36.6,40.9)<br>(36.6,40.9)
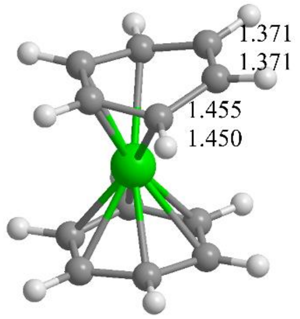<br>V4-66Q(C_s)<br>(42.2,43.4)<br>(39.7,39.5)	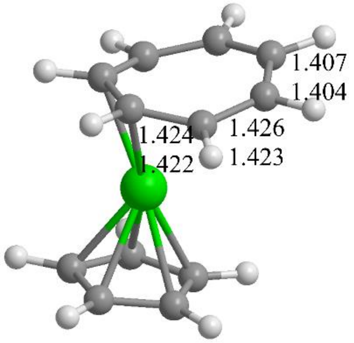<br>V5-57X(C_s)<br>(57.6,61.9)<br>(57.5,61.9)	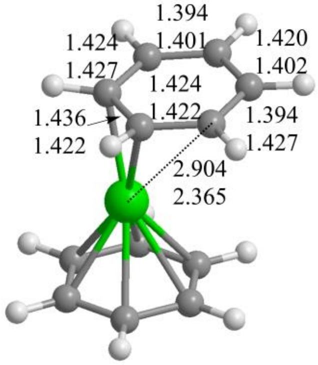<br>V6-66X(C_1)<br>(60.2,61.9)<br>(57.6,58.0)
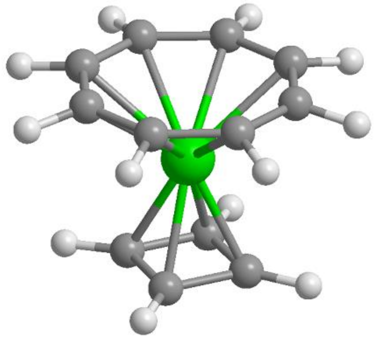<br>V7-48D(C_{4v})<br>(74.3,68.8)<br>(0.0,0.0)	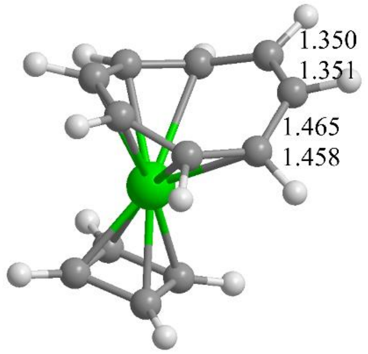<br>V8-48Q(C_s)<br>(114.9,111.3)<br>(40.6,42.6)	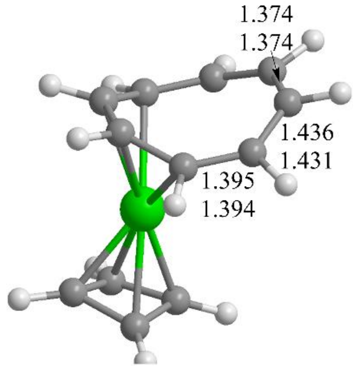<br>V9-48X(C_s)<br>(137.0,139.0)<br>(62.8,70.2)

Table S3. Optimized (C_nH_n)Cr(C_mH_m) (n=6,5,4;m=6,7,8) structures

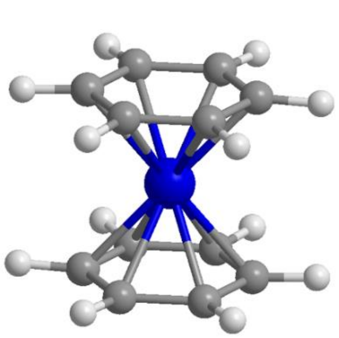 Cr1-66S(D_{6h})<br>(0.0,0.0)<br>(0.0,0.0)	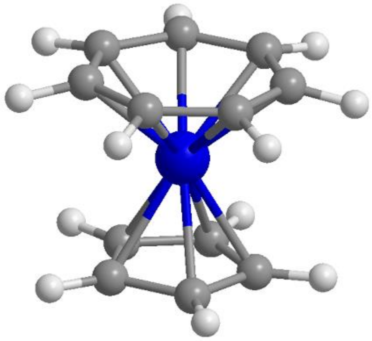 Cr2-57S(C_s)<br>(11.0,9.7)<br>(0.0,0.0)	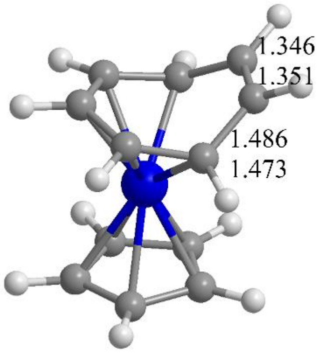 Cr3-57T(C_s)<br>(24.1,24.9)<br>(13.1,15.2)
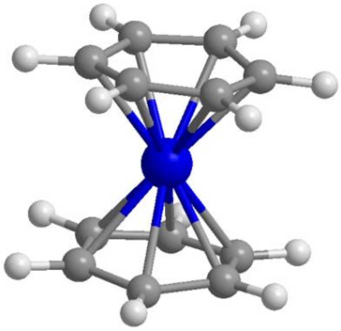 Cr4-66T(D_2 ,C_s)<br>(25.0,27.3)<br>(25.0,27.3)	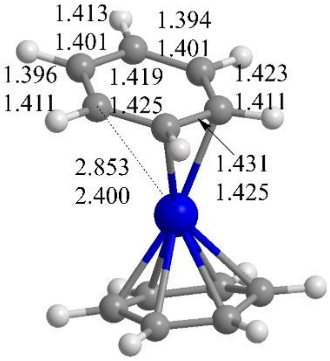 Cr5-66P(C_1 ,C_s)<br>(32.6, 35.0)<br>(32.6, 35.0)	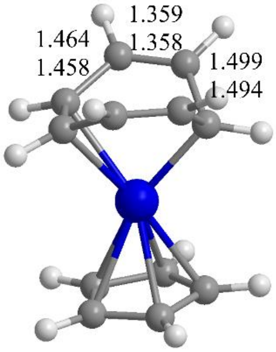 Cr6-57P(C_s)<br>(41.2,47.0)<br>(30.2,37.3)
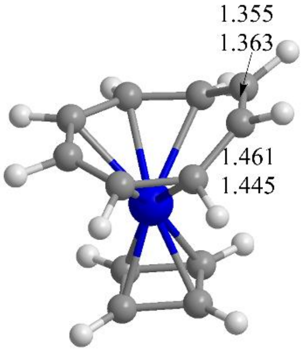 Cr7-48T(C_s)<br>(85.2,79.5)<br>(0.0,0.0)	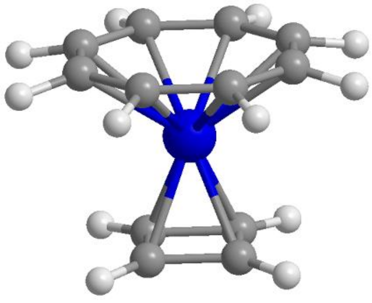 Cr8-48S(C_{4v})<br>(99.9,93.2)<br>(14.7,13.7)	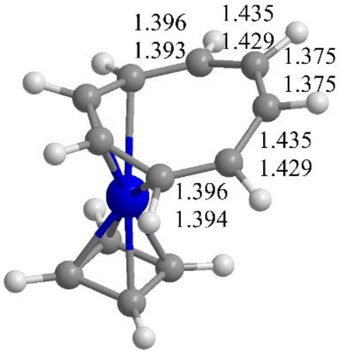 Cr9-48P(C_1)<br>(96.6,93.8)<br>(11.4,14.3)

Table S4. Optimized $(C_nH_n)Mn(C_mH_m)$ (n=6,5,4;m=6,7,8) structures

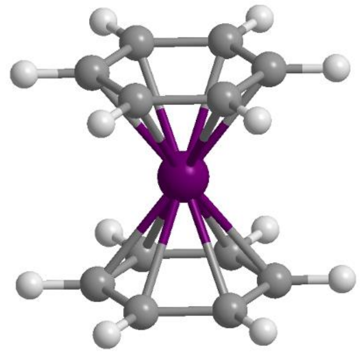<br>Mn1-66Q (D_{6h})<br>(0.0,0.0)<br>(0.0,0.0)	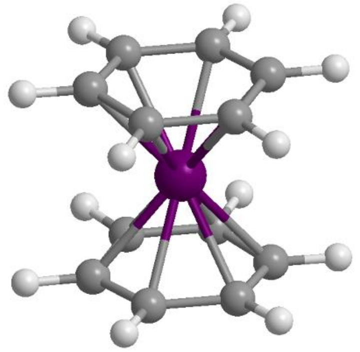<br>Mn2-66D(C_1)<br>(5.6,5.3)<br>(5.6,5.3)	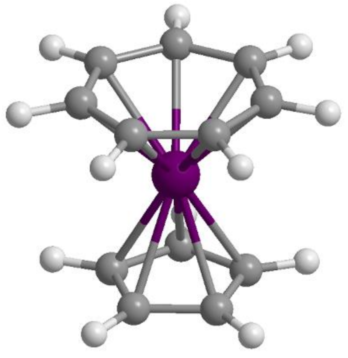<br>Mn3-57Q(C_s)<br>(5.7,5.5)<br>(0.0,0.0)
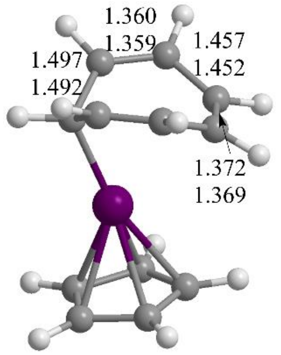<br>Mn4-57X(C_s)<br>(13.2,17.4)<br>(7.5,11.9)	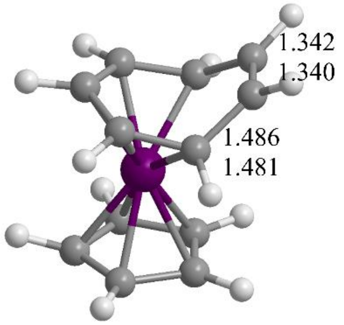<br>Mn5-57D(C_s)<br>(22.0,13.4)<br>(16.3,7.9)	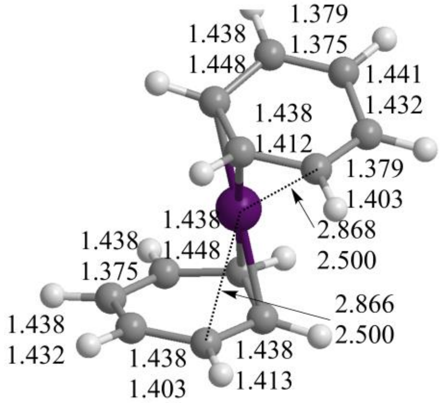<br>Mn6-66X(C_1)<br>(20.7,24.0)<br>(20.7,24.0)
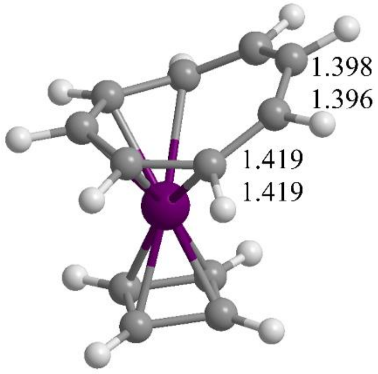<br>Mn7-48Q(C_s)<br>(69.3,60.1)<br>(0.0,0.0)	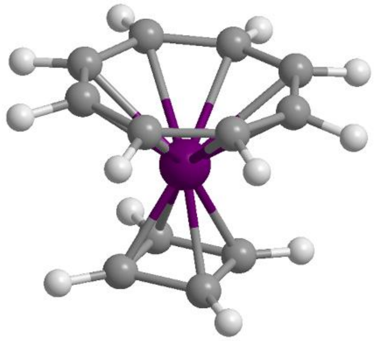<br>Mn8-48D (C_{4v})<br>(86.6,77.0)<br>(17.4, 16.9)	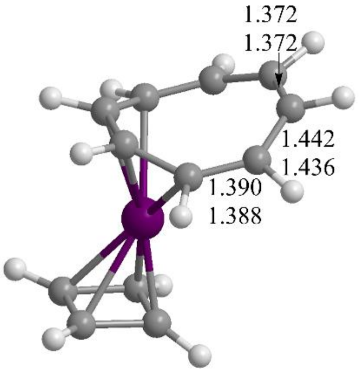<br>Mn9-48X(C_s)<br>(91.7,85.2)<br>(22.4,25.1)

Table S5. Optimized $(C_nH_n)Fe(C_mH_m)$ (n=6,5,4;m=6,7,8) structures

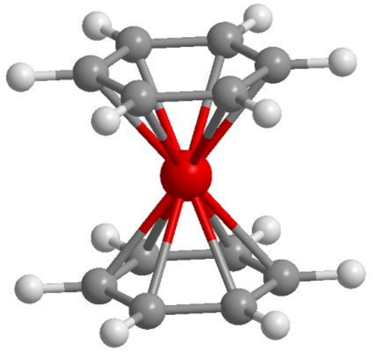<br>Fe1-66T (D_{6h})<br>(0.0,0.0)<br>(0.0,0.0)	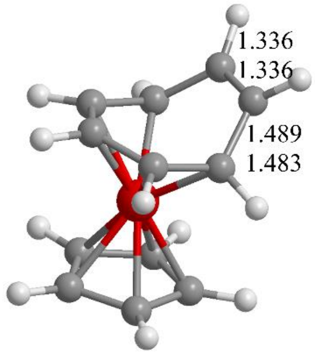<br>Fe2-57S(C_s)<br>(12.2,5.7)<br>(0.0,0.0)	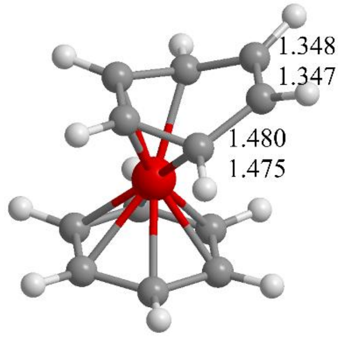<br>Fe3-66S(C_s)<br>(14.9,6.9)<br>(14.9,6.9)
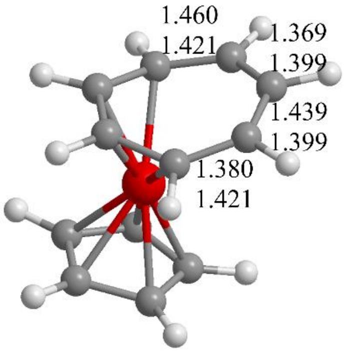<br>Fe4-57T(C_1)<br>(20.0,17.6)<br>(7.8,11.9)	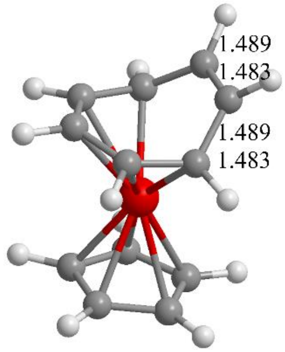<br>Fe5-57P(C_S)<br>(30.4,31.7)<br>(18.3,26.0)	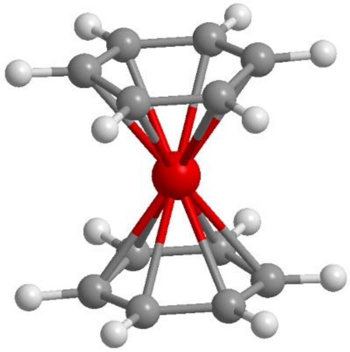<br>Fe6-66P(C_2)<br>(36.9,38.2)<br>(36.9,38.2)
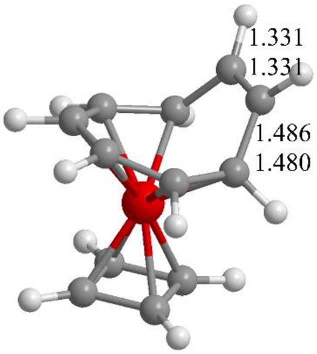<br>Fe7-48S(C_s)<br>(72.9,58.6)<br>(0.0,0.0)	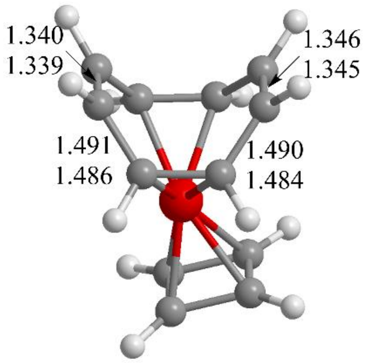<br>Fe8-48T(C_s)<br>(88.7,80.0)<br>(15.8,21.6)	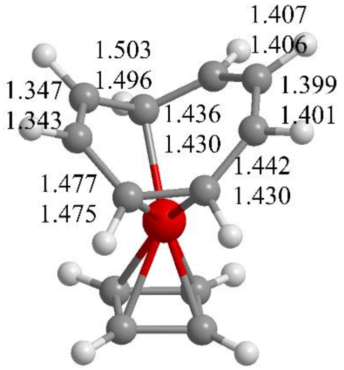<br>Fe9-48P(C_1)<br>(105.3,96.4)<br>(32.4,37.8)

Table S6. Optimized (C_nH_n)Co(C_mH_m) (n=6,5,4;m=6,7,8) structures

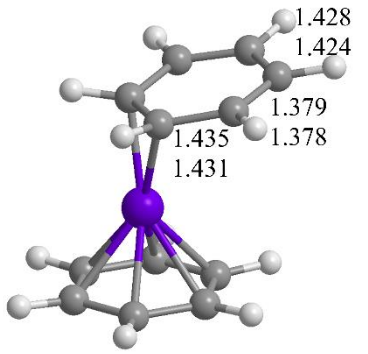<br>Co1-66D(C_s)<br>(0.0,0.0)<br>(0.0,0.0)	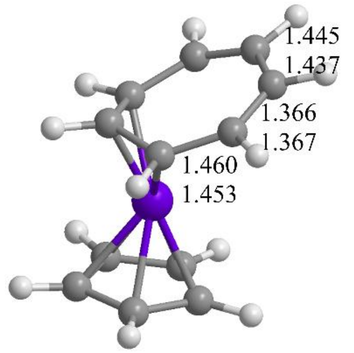<br>Co2-57D(C_s)<br>(13.4,11.7)<br>(0.0,0.0)	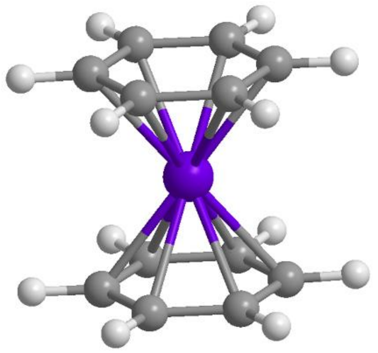<br>Co3-66Q(C_2,C_{2h})<br>(23.8,26.8)<br>(23.8,26.8)
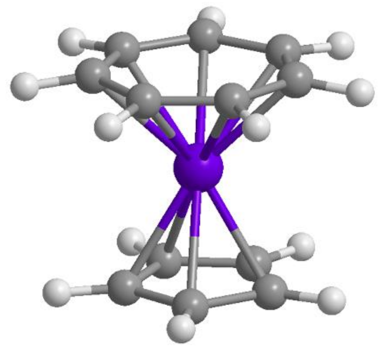<br>Co4-57Q(C_s)<br>(27.3,27.8)<br>(13.9,16.1)	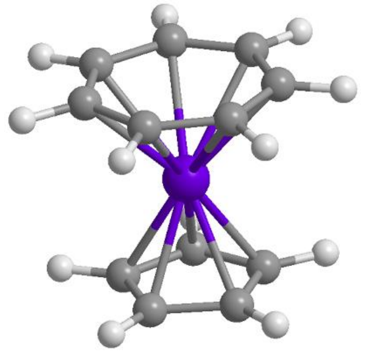<br>Co5-57X(C_s)<br>(68.4,61.4)<br>(55.1,49.7)	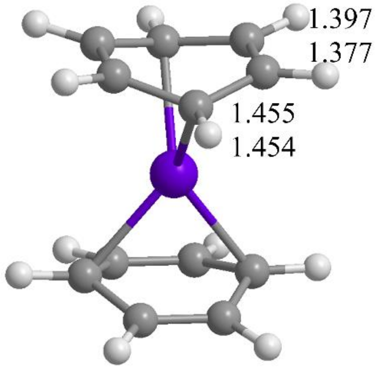<br>Co6-66X(D_{2d})<br>(71.5,83.9)<br>(71.5,83.9)
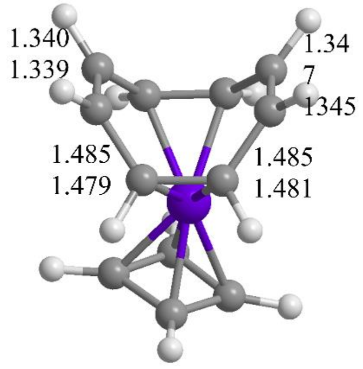<br>Co7-48D(C_s)<br>(86.6,77.4)<br>(0.0,0.0)	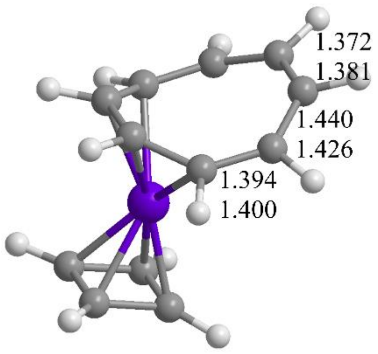<br>Co8-48Q(C_s)<br>(101.5,97.8)<br>(14.9,20.4)	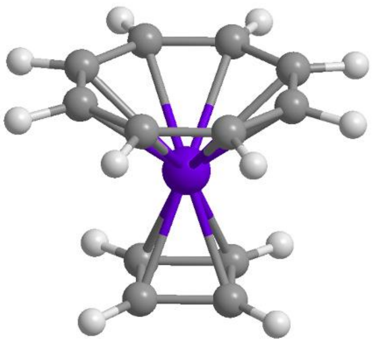<br>Co9-48X(C_{4v})<br>(129.8,122.2)<br>(43.2,44.8)

Table S7. Optimized $(C_nH_n)Ni(C_mH_m)$ (n=6,5,4;m=6,7,8) structures

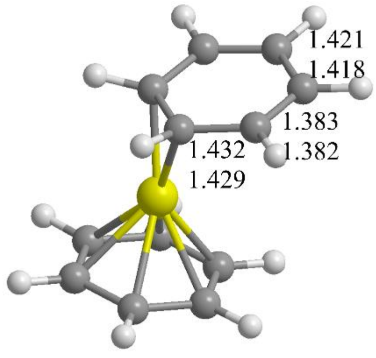<br>Ni1-66S(*Cs*)<br>(0.0,0.0)<br>(0.0,0.0)	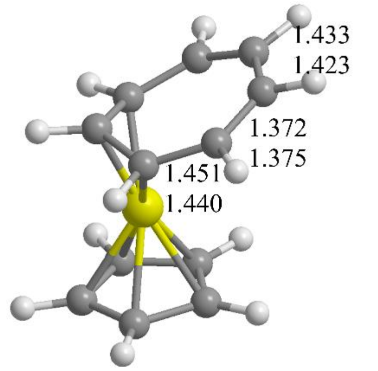<br>Ni2-57S(*Cs*)<br>(18.1,14.4)<br>(0.0,0.0)	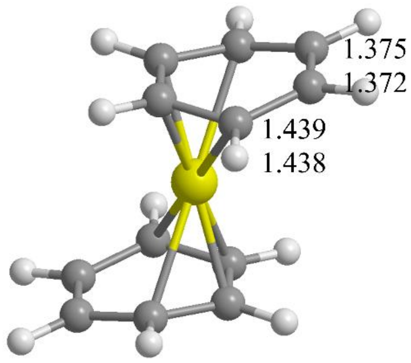<br>Ni3-66T(*C2h*)<br>(33.6,37.9)<br>(33.6,37.9)
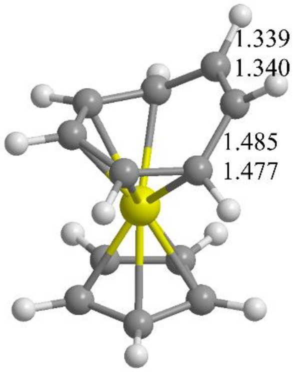<br>Ni4-57T(*Cs*)<br>(38.7,41.4)<br>(20.6,26.9)	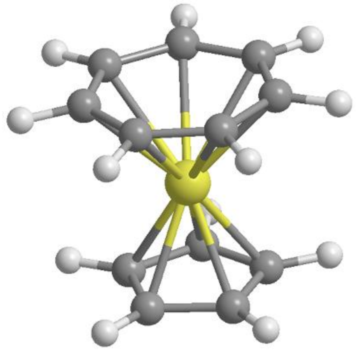<br>Ni5-57P(*Cs*)<br>(60.3,62.5)<br>(42.2,48.0)	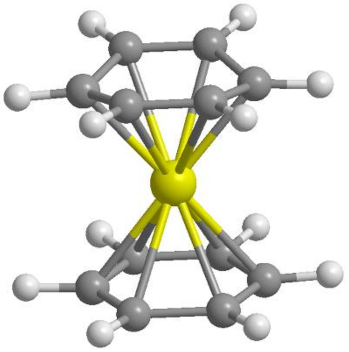<br>Ni6-66P(*C2v*)<br>(84.2,88.1)<br>(84.2,88.1)
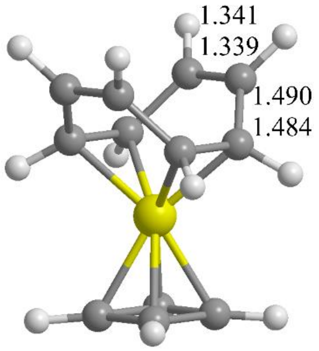<br>Ni7-48S(*C2v*)<br>(86.4,80.6)<br>(0.0,0.0)	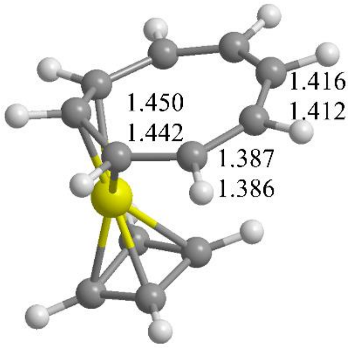<br>Ni8-48T(*Cs*)<br>(101.4,95.6)<br>(15.0,15.0)	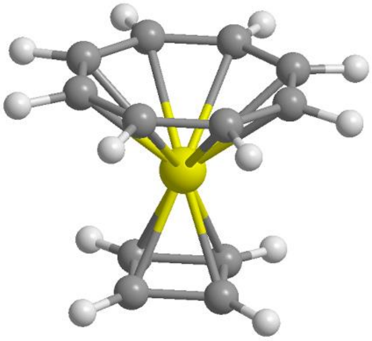<br>Ni9-48P(*C4v*)<br>(136.7,127.4)<br>(50.3,46.9)

Table S8. Harmonic vibrational frequencies (in cm^{-1}) and infrared intensities (in parentheses, in km/mol) for the $C_5H_5TiC_7H_7$ structure **Ti1-57S**

B3PW91			M06-L		
4(0)	797(3)	1349(0)	19(0)	772(3)	1344(0)
101(0)	797(0)	1419(0)	91(0)	783(86)	1423(0)
102(0)	850(0)	1419(0)	94(0)	844(0)	1423(0)
250(0)	850(0)	1427(0)	255(0)	844(0)	1424(3)
290(0)	866(26)	1427(0)	301(0)	861(0)	1425(3)
290(0)	881(0)	1463(0)	302(0)	862(0)	1464(0)
329(0)	882(0)	1463(0)	328(0)	865(32)	1465(0)
329(0)	888(0)	1502(0)	329(0)	868(0)	1494(0)
420(5)	888(0)	1502(0)	420(4)	869(0)	1495(0)
421(6)	914(0)	1529(0)	421(4)	911(0)	1541(0)
454(1)	914(0)	1529(0)	452(0)	911(0)	1541(0)
492(0)	967(22)	3200(0)	491(0)	966(23)	3176(0)
492(0)	968(21)	3200(0)	491(0)	967(22)	3176(0)
538(0)	1026(17)	3208(0)	526(0)	1028(14)	3184(0)
539(0)	1027(18)	3208(0)	527(0)	1029(15)	3184(0)
612(0)	1073(0)	3220(10)	614(0)	1074(0)	3196(45)
612(0)	1074(0)	3221(9)	615(0)	1074(0)	3197(44)
755(10)	1145(8)	3228(0)	731(11)	1147(13)	3205(1)
755(10)	1175(0)	3250(0)	732(11)	1173(0)	3222(0)
758(154)	1175(0)	3250(0)	745(119)	1173(0)	3222(0)
791(93)	1242(0)	3264(1)	766(0)	1234(0)	3239(16)
794(3)	1242(0)	3264(1)	766(0)	1235(0)	3239(16)
795(3)	1266(0)	3276(0)	772(4)	1258(0)	3252(0)

Table S9. Harmonic vibrational frequencies (in cm^{-1}) and infrared intensities (in parentheses, in km/mol) for the $C_6H_6TiC_6H_6$ structure **Ti2-66S**

B3PW91			M06-L		
39(0)	769(2)	1315(0)	28(0)	740(0)	1312(0)
72(0)	769(2)	1419(0)	53(0)	740(0)	1431(0)
72(0)	816(0)	1430(0)	53(0)	776(0)	1432(1)
256(0)	816(0)	1432(0)	247(0)	776(0)	1432(1)
301(0)	836(0)	1432(0)	293(0)	803(0)	1433(0)
301(0)	836(0)	1434(0)	293(0)	803(0)	1433(0)
387(0)	879(0)	1434(0)	371(123)	851(0)	1446(0)
387(0)	881(0)	1454(0)	380(0)	853(0)	1449(0)
410(153)	970(58)	1454(0)	380(0)	969(78)	1449(0)
443(0)	970(0)	1528(0)	439(0)	971(0)	1531(0)
443(0)	1002(36)	1528(0)	439(0)	1003(0)	1531(0)
457(18)	1002(36)	3226(0)	444(13)	1003(0)	3200(0)
457(18)	1003(0)	3227(0)	444(13)	1003(34)	3201(0)
600(0)	1003(0)	3230(0)	584(0)	1003(34)	3206(0)
604(0)	1018(0)	3230(0)	592(0)	1017(0)	3206(0)
604(0)	1035(0)	3231(0)	592(0)	1034(0)	3206(0)
611(0)	1117(0)	3231(0)	598(0)	1116(0)	3206(0)
611(0)	1117(0)	3242(0)	603(0)	1116(0)	3219(0)
617(0)	1135(0)	3242(0)	603(0)	1128(0)	3219(0)
713(108)	1138(0)	3243(9)	696(91)	1131(0)	3219(60)
729(0)	1149(0)	3243(9)	709(0)	1150(0)	3219(60)
766(0)	1149(0)	3251(0)	739(2)	1150(0)	3228(0)
732(0)	1334(0)	3242(0)	739(2)	1310(0)	3228(4)

Table S10. Harmonic vibrational frequencies (in cm^{-1}) and infrared intensities (in parentheses, in km/mol) for the $C_5H_5TiC_7H_7$ structure **Ti3-57T**

B3PW91			M06-L		
10(0)	804(3)	1277(2)	1(0)	787(3)	1273(2)
33(0)	841(0)	1365(0)	15(0)	829(0)	1362(0)
99(0)	842(0)	1414(0)	85(0)	832(0)	1418(0)
100(0)	856(8)	1418(0)	86(0)	859(0)	1422(0)
230(1)	878(0)	1429(5)	232(2)	861(11)	1431(1)
259(1)	881(0)	1440(0)	255(1)	861(0)	1441(0)
270(0)	896(0)	1466(1)	288(0)	895(0)	1467(0)
285(0)	903(7)	1466(0)	290(0)	897(1)	1467(0)
323(1)	912(1)	1493(4)	319(1)	901(3)	1493(1)
377(6)	918(0)	1548(18)	392(5)	909(1)	1553(19)
382(5)	923(1)	1561(2)	393(5)	912(5)	1563(3)
431(3)	957(7)	3183(3)	433(4)	959(6)	3149(10)
466(0)	960(9)	3185(1)	474(0)	964(8)	3150(15)
483(0)	1026(15)	3189(1)	488(0)	1028(12)	3157(0)
520(11)	1027(15)	3199(6)	514(9)	1028(12)	3169(23)
613(0)	1071(0)	3209(18)	612(0)	1071(0)	3179(65)
614(0)	1072(0)	3211(15)	613(0)	1071(0)	3181(44)
752(132)	1146(3)	3216(2)	745(89)	1148(6)	3185(10)
777(0)	1149(17)	3237(0)	758(0)	1148(16)	3207(0)
778(3)	1160(1)	3238(0)	759(0)	1161(0)	3209(0)
786(115)	1197(0)	3253(4)	772(4)	1195(1)	3227(27)
796(5)	1249(1)	3254(4)	776(95)	1243(1)	3227(27)
796(2)	1267(0)	3266(0)	782(3)	1259(0)	3241(4)

Table S11. Harmonic vibrational frequencies (in cm^{-1}) and infrared intensities (in parentheses, in km/mol) for the $C_6H_6TiC_6H_6$ structure **Ti4-66T**

B3PW91			M06-L		
39(0)	808(39)	1329(0)	48(0)	777(0)	1327(0)
81(0)	817(0)	1397(0)	74(1)	806(0)	1410(0)
85(1)	828(1)	1399(24)	75(1)	811(0)	1413(17)
225(0)	837(0)	1418(0)	225(0)	815(1)	1414(0)
237(0)	880(0)	1443(3)	236(0)	846(0)	1446(0)
271(0)	880(0)	1444(0)	280(0)	862(0)	1447(0)
281(0)	917(0)	1462(0)	288(0)	898(0)	1464(0)
341(38)	919(0)	1467(0)	336(35)	900(0)	1472(2)
403(101)	971(78)	1470(0)	398(0)	976(90)	1472(0)
407(0)	975(0)	1502(32)	401(84)	979(0)	1506(30)
413(20)	994(0)	1573(0)	417(17)	998(0)	1578(0)
415(0)	997(30)	3206(0)	418(0)	1002(26)	3174(0)
421(11)	1007(0)	3207(0)	426(9)	1013(0)	3175(9)
598(0)	1017(1)	3207(2)	594(0)	1022(1)	3175(0)
599(0)	1023(0)	3207(0)	600(0)	1027(0)	3177(0)
609(0)	1025(12)	3216(3)	607(0)	1028(9)	3186(9)
609(0)	1087(0)	3217(0)	609(0)	1084(0)	3187(0)
620(0)	1117(0)	3222(0)	615(0)	1119(0)	3191(0)
634(0)	1140(5)	3222(34)	625(0)	1142(5)	3192(122)
717(57)	1148(0)	3232(0)	703(29)	1143(0)	3204(0)
735(0)	1151(0)	3233(21)	719(0)	1145(0)	3204(89)
780(0)	1164(0)	3236(0)	761(0)	1164(0)	3207(13)
796(0)	1328(0)	3236(1)	771(44)	1326(0)	3207(0)

Table S12. Harmonic vibrational frequencies (in cm^{-1}) and infrared intensities (in parentheses, in km/mol) for the $C_6H_6TiC_6H_6$ structure **Ti5-66P**

B3PW91			M06-L		
27(0)	706(0)	1331(0)	37(0)	685(0)	1328(0)
87(0)	785(6)	1331(0)	41(1)	760(8)	1328(0)
87(0)	792(0)	1385(12)	41(1)	772(0)	1398(7)
153(0)	813(1)	1385(12)	120(0)	800(1)	1398(7)
153(0)	813(1)	1432(0)	120(0)	800(1)	1436(0)
198(0)	917(0)	1432(0)	200(0)	891(0)	1436(0)
225(0)	917(0)	1468(0)	230(0)	897(0)	1476(0)
225(0)	920(0)	1476(0)	230(0)	897(0)	1476(0)
295(2)	921(0)	1476(0)	263(0)	899(0)	1479(0)
303(0)	952(131)	1548(63)	295(1)	956(145)	1543(42)
388(0)	964(31)	1590(0)	312(0)	971(26)	1592(0)
392(69)	964(31)	3198(0)	394(63)	971(26)	3170(0)
429(0)	971(0)	3205(2)	424(0)	975(0)	3176(6)
566(0)	998(3)	3205(2)	518(0)	1001(4)	3176(6)
569(2)	998(3)	3205(0)	555(3)	1001(4)	3178(0)
569(2)	1029(4)	3217(0)	555(3)	1031(4)	3189(9)
578(0)	1029(4)	3217(5)	578(0)	1031(4)	3189(0)
580(1)	1125(0)	3221(15)	582(2)	1130(0)	3194(54)
606(0)	1151(0)	3221(15)	604(0)	1145(0)	3194(54)
640(0)	1151(0)	3237(5)	621(0)	1145(0)	3210(30)
675(270)	1163(3)	3237(5)	654(214)	1160(2)	3210(30)
683(0)	1169(0)	3241(2)	663(0)	1169(0)	3215(2)
706(0)	1281(0)	3242(0)	685(0)	1274(0)	3215(0)

Table S13. Harmonic vibrational frequencies (in cm^{-1}) and infrared intensities (in parentheses, in km/mol) for the $C_4H_4TiC_8H_8$ structure **Ti6-48S**

B3PW91			M06-L		
17(0)	773(18)	1334(0)	4(0)	772(49)	1333(0)
107(1)	788(141)	1359(0)	101(1)	782(11)	1365(1)
107(1)	795(13)	1359(0)	101(1)	782(11)	1365(1)
235(0)	795(13)	1391(0)	236(0)	791(109)	1389(0)
238(0)	860(0)	1445(0)	238(0)	844(0)	1447(1)
242(1)	869(0)	1445(0)	247(1)	854(0)	1447(1)
317(0)	928(6)	1531(0)	330(0)	924(0)	1529(0)
317(0)	928(6)	1532(0)	330(0)	924(0)	1530(0)
368(4)	936(0)	1543(1)	382(2)	933(7)	1543(1)
368(4)	939(4)	1543(1)	382(2)	933(7)	1543(1)
388(0)	939(4)	1593(0)	400(0)	934(0)	1606(0)
393(0)	943(28)	3184(0)	408(0)	941(0)	3162(0)
504(0)	943(28)	3188(0)	506(0)	948(27)	3166(0)
504(0)	956(0)	3188(0)	506(0)	948(27)	3166(0)
512(0)	965(0)	3199(0)	513(0)	971(0)	3176(0)
567(0)	972(0)	3199(0)	549(0)	973(0)	3176(0)
599(0)	1164(0)	3211(14)	549(0)	1154(0)	3188(55)
599(0)	1207(0)	3211(14)	566(0)	1208(0)	3188(55)
653(0)	1207(0)	3217(1)	629(0)	1208(0)	3194(3)
740(73)	1246(0)	3240(0)	728(46)	1268(0)	3199(0)
764(0)	1281(17)	3257(1)	759(0)	1286(21)	3219(20)
764(0)	1324(0)	3257(1)	771(0)	1319(0)	3219(20)
768(0)	1334(0)	3280(2)	771(0)	1333(0)	3246(0)

Table S14. Harmonic vibrational frequencies (in cm^{-1}) and infrared intensities (in parentheses, in km/mol) for the $C_5H_5TiC_7H_7$ structure **Ti7-57P**

B3PW91				M06-L		
7(0)	835(0)	1375(0)		11(0)	807(3)	1373(0)
59(1)	837(1)	1401(0)		67(1)	821(1)	1390(5)
74(0)	840(0)	1415(1)		75(0)	826(0)	1402(3)
81(0)	854(7)	1426(4)		87(1)	843(0)	1419(0)
85(0)	864(0)	1447(3)		105(1)	858(11)	1448(1)
190(3)	880(0)	1447(3)		193(2)	862(0)	1449(1)
235(0)	884(0)	1463(1)		240(1)	870(0)	1463(2)
269(2)	887(0)	1472(1)		253(3)	882(2)	1471(1)
281(0)	890(1)	1508(2)		288(1)	887(2)	1515(0)
318(1)	900(2)	1523(0)		340(1)	897(1)	1528(0)
389(18)	907(1)	1542(70)		398(9)	900(1)	1553(77)
414(1)	952(9)	3186(1)		418(2)	959(7)	3157(1)
428(0)	961(7)	3186(1)		429(0)	962(5)	3158(3)
470(3)	1016(21)	3192(1)		477(4)	1006(22)	3164(1)
518(1)	1022(19)	3197(2)		513(1)	1020(18)	3169(5)
609(0)	1067(0)	3207(18)		595(1)	1064(0)	3179(62)
610(0)	1073(0)	3209(16)		599(0)	1072(0)	3182(51)
667(127)	1139(7)	3217(3)		651(85)	1135(20)	3190(12)
744(10)	1160(0)	3234(0)		721(8)	1160(1)	3205(0)
756(13)	1174(0)	3235(0)		727(13)	1181(0)	3207(0)
769(16)	1250(0)	3250(4)		742(7)	1241(1)	3224(28)
773(22)	1256(2)	3250(3)		755(4)	1251(2)	3225(26)
790(69)	1265(0)	3263(0)		777(57)	1256(0)	3238(2)

Table S15. Harmonic vibrational frequencies (in cm^{-1}) and infrared intensities (in parentheses, in km/mol) for the $C_4H_4TiC_8H_8$ structure **Ti8-48T**

B3PW91			M06-L		
12(0)	760(0)	1332(0)	-1(0)	759(0)	1327(0)
91(0)	774(0)	1337(0)	66(0)	766(0)	1336(0)
100(1)	781(22)	1367(0)	87(1)	783(21)	1374(0)
226(0)	810(9)	1395(0)	218(0)	802(7)	1395(0)
227(0)	814(9)	1451(0)	225(0)	808(7)	1452(2)
230(0)	873(0)	1451(0)	229(0)	855(3)	1454(1)
249(6)	875(2)	1538(0)	276(1)	862(0)	1538(0)
273(1)	899(4)	1546(0)	283(4)	909(5)	1543(0)
320(12)	930(15)	1558(0)	328(14)	911(2)	1559(0)
379(4)	932(10)	1561(0)	386(3)	916(0)	1564(0)
385(0)	932(0)	1611(6)	391(0)	916(0)	1623(3)
386(0)	933(3)	3163(0)	395(0)	933(15)	3133(0)
427(12)	935(0)	3166(0)	427(13)	934(0)	3136(1)
497(0)	935(0)	3170(0)	490(0)	937(2)	3139(1)
498(0)	944(41)	3179(0)	494(0)	946(37)	3148(0)
545(41)	971(0)	3180(0)	529(27)	966(0)	3149(0)
565(0)	1050(0)	3190(37)	543(9)	1046(0)	3158(111)
576(11)	1166(0)	3193(38)	555(0)	1154(0)	3160(116)
659(0)	1206(0)	3198(1)	624(0)	1208(0)	3165(6)
663(0)	1213(0)	3231(0)	695(10)	1213(0)	3180(2)
715(33)	1259(8)	3237(3)	725(0)	1265(13)	3185(22)
758(0)	1283(17)	3271(0)	747(28)	1285(17)	3240(9)
759(118)	1328(0)	3279(1)	754(70)	1323(0)	3247(1)

Table S16. Harmonic vibrational frequencies (in cm^{-1}) and infrared intensities (in parentheses, in km/mol) for the $C_4H_4TiC_8H_8$ structure **Ti9-48P**

B3PW91				M06-L		
58(0)	727(24)	1286(1)		60(1)	706(10)	1277(0)
79(0)	759(0)	1290(0)		76(1)	734(18)	1284(0)
86(1)	763(2)	1360(0)		86(1)	764(2)	1367(0)
127(5)	800(5)	1379(0)		122(1)	796(3)	1375(0)
135(0)	808(3)	1421(8)		157(1)	808(2)	1420(3)
142(0)	851(11)	1430(3)		159(0)	840(19)	1427(0)
246(1)	883(0)	1483(0)		242(0)	868(0)	1479(1)
253(0)	893(2)	1520(4)		244(1)	902(3)	1521(3)
301(5)	907(1)	1551(5)		308(1)	904(2)	1543(5)
315(1)	920(1)	1588(10)		324(0)	904(3)	1580(12)
332(11)	932(10)	1689(23)		347(18)	917(5)	1694(27)
357(1)	932(2)	3143(1)		366(2)	918(1)	3113(2)
399(12)	938(6)	3151(3)		401(4)	935(3)	3122(8)
420(1)	938(35)	3154(1)		420(2)	937(4)	3125(3)
450(0)	949(3)	3165(16)		445(0)	943(26)	3135(45)
543(29)	956(0)	3166(9)		527(18)	953(1)	3136(26)
551(3)	1043(0)	3174(25)		537(6)	1038(0)	3144(52)
571(25)	1105(0)	3177(29)		561(17)	1099(0)	3147(73)
630(0)	1162(0)	3189(12)		642(9)	1151(0)	3158(39)
652(36)	1211(2)	3222(1)		648(18)	1206(6)	3165(11)
670(0)	1237(22)	3230(5)		687(1)	1237(36)	3174(28)
698(5)	1261(0)	3259(0)		694(7)	1248(0)	3223(15)
712(48)	1272(22)	3268(0)		695(4)	1271(19)	3229(7)

Table S17. Harmonic vibrational frequencies (in cm^{-1}) and infrared intensities (in parentheses, in km/mol) for the $C_5H_5VC_7H_7$ structure **V1-57D**

B3PW91			M06-L		
8(0)	844(0)	1361(0)	9(0)	821(0)	1356(0)
138(0)	844(0)	1419(0)	127(0)	821(0)	1422(0)
139(0)	847(0)	1419(0)	131(0)	835(0)	1422(0)
254(1)	847(0)	1442(0)	252(1)	835(0)	1440(2)
307(0)	874(16)	1442(0)	313(0)	864(0)	1440(2)
307(0)	887(0)	1461(1)	313(0)	866(0)	1461(0)
320(0)	887(0)	1462(1)	319(0)	875(19)	1461(0)
320(0)	900(0)	1528(0)	320(0)	890(0)	1521(0)
438(16)	900(0)	1528(0)	438(5)	891(0)	1522(0)
438(16)	923(0)	1544(0)	441(14)	910(0)	1560(0)
451(7)	923(0)	1544(0)	442(15)	910(0)	1561(0)
493(0)	980(13)	3189(0)	497(0)	980(13)	3157(0)
493(0)	980(13)	3189(0)	497(0)	981(13)	3157(0)
535(0)	1025(15)	3197(0)	524(0)	1027(12)	3165(0)
535(0)	1026(15)	3197(0)	525(0)	1027(12)	3165(0)
609(0)	1071(0)	3208(21)	607(0)	1070(0)	3176(76)
610(0)	1072(0)	3209(20)	608(0)	1071(0)	3177(73)
794(1)	1144(9)	3216(0)	774(1)	1144(14)	3184(3)
794(1)	1187(0)	3247(0)	775(0)	1184(0)	3217(0)
795(144)	1187(0)	3247(0)	785(1)	1185(0)	3217(0)
796(48)	1252(0)	3262(3)	788(143)	1244(0)	3235(22)
817(14)	1252(0)	3262(3)	798(14)	1245(0)	3236(22)
817(13)	1265(0)	3274(0)	799(13)	1257(0)	3248(2)

Table S18. Harmonic vibrational frequencies (in cm^{-1}) and infrared intensities (in parentheses, in km/mol) for the $C_6H_6VC_6H_6$ structure **V2-66D**

B3PW91			M06-L		
41(0)	816(6)	1319(0)	37(0)	795(5)	1316(0)
126(0)	816(6)	1434(0)	115(0)	795(5)	1440(0)
126(0)	851(0)	1440(1)	115(0)	818(0)	1440(0)
262(0)	851(0)	1440(1)	255(0)	818(0)	1442(0)
318(0)	857(0)	1441(0)	318(0)	822(0)	1442(0)
318(0)	857(0)	1442(0)	318(0)	822(0)	1445(0)
395(0)	901(0)	1442(0)	388(0)	873(0)	1456(0)
395(0)	903(0)	1481(0)	388(0)	877(0)	1476(0)
434(165)	980(0)	1481(0)	409(139)	981(55)	1476(0)
444(0)	981(41)	1540(0)	441(0)	981(0)	1543(0)
444(0)	1010(0)	1540(0)	441(0)	1009(0)	1543(0)
478(29)	1010(0)	3216(0)	475(26)	1011(0)	3184(0)
478(29)	1010(0)	3217(0)	475(26)	1011(0)	3185(0)
613(0)	1011(24)	3219(0)	600(0)	1013(21)	3190(0)
617(0)	1011(24)	3219(0)	608(0)	1013(21)	3190(0)
617(0)	1025(0)	3221(0)	613(0)	1025(0)	3190(0)
619(0)	1128(0)	3221(0)	613(0)	1126(0)	3190(0)
619(0)	1128(0)	3232(0)	616(0)	1126(0)	3202(0)
624(0)	1141(0)	3232(0)	616(0)	1135(0)	3202(0)
755(86)	1145(0)	3234(24)	743(65)	1138(0)	3203(102)
761(0)	1151(0)	3234(24)	745(0)	1151(0)	3203(102)
803(0)	1151(0)	3241(2)	782(0)	1151(0)	3210(14)
803(0)	1317(0)	3242(0)	782(0)	1314(0)	3211(0)

Table S19. Harmonic vibrational frequencies (in cm^{-1}) and infrared intensities (in parentheses, in km/mol) for the $C_5H_5VC_7H_7$ structure **V3-57Q**

B3PW91			M06-L		
16(0)	817(8)	1338(6)	-15(0)	802(7)	1337(4)
75(0)	845(0)	1384(26)	66(0)	835(0)	1384(17)
99(0)	846(0)	1411(0)	84(0)	837(0)	1412(0)
166(2)	875(2)	1414(1)	143(6)	860(0)	1415(1)
177(4)	882(4)	1415(2)	153(2)	866(0)	1418(0)
243(3)	887(1)	1462(1)	240(2)	870(5)	1460(0)
274(3)	888(0)	1466(1)	280(3)	876(6)	1466(0)
315(2)	899(18)	1476(25)	324(1)	891(12)	1473(24)
341(5)	922(13)	1491(5)	344(2)	916(12)	1488(7)
343(2)	944(0)	1554(33)	348(4)	927(0)	1549(30)
400(1)	967(4)	1727(28)	399(1)	949(6)	1720(31)
411(5)	976(7)	3148(3)	409(5)	962(7)	3118(18)
429(0)	984(3)	3151(7)	428(0)	983(5)	3119(11)
556(36)	1024(17)	3153(1)	548(28)	1025(14)	3123(0)
567(2)	1026(17)	3167(26)	560(2)	1027(14)	3138(26)
610(0)	1070(0)	3173(37)	602(0)	1071(0)	3142(95)
612(0)	1073(0)	3178(19)	608(0)	1073(0)	3152(67)
731(29)	1128(1)	3210(7)	723(24)	1129(1)	3183(24)
757(1)	1144(5)	3240(0)	743(0)	1144(11)	3213(0)
788(118)	1170(8)	3243(0)	766(7)	1167(7)	3216(1)
790(2)	1212(3)	3256(2)	777(1)	1211(3)	3232(21)
798(2)	1219(6)	3257(2)	778(69)	1216(5)	3233(21)
798(28)	1266(0)	3270(0)	786(27)	1258(0)	3246(3)

Table S20. Harmonic vibrational frequencies (in cm^{-1}) and infrared intensities (in parentheses, in km/mol) for the $C_6H_6VC_6H_6$ structure **V4-66Q**

B3PW91			M06-L		
37(0)	799(0)	1324(0)	46(0)	780(0)	1322(0)
70(0)	821(23)	1374(10)	61(1)	793(13)	1385(7)
104(0)	825(2)	1386(24)	101(0)	807(4)	1396(18)
160(0)	849(4)	1416(3)	141(0)	825(2)	1415(0)
228(2)	909(2)	1423(0)	228(0)	887(1)	1425(0)
237(1)	915(0)	1435(2)	232(2)	890(0)	1437(0)
295(8)	927(0)	1453(0)	283(6)	908(0)	1456(0)
314(58)	934(0)	1484(1)	302(46)	912(0)	1484(2)
341(4)	951(19)	1490(7)	347(5)	952(19)	1493(10)
382(1)	959(106)	1559(60)	383(1)	962(105)	1558(50)
389(7)	974(7)	1606(36)	389(9)	975(6)	1603(21)
440(6)	981(19)	3191(0)	432(1)	982(16)	3165(2)
441(1)	995(2)	3198(0)	437(4)	1000(2)	3170(1)
553(0)	1010(2)	3206(7)	538(0)	1011(2)	3177(4)
573(1)	1028(4)	3207(1)	574(0)	1031(3)	3179(0)
599(1)	1041(3)	3209(0)	592(0)	1043(2)	3180(11)
604(0)	1093(0)	3214(22)	598(0)	1094(0)	3188(59)
611(0)	1122(0)	3220(4)	605(0)	1124(0)	3191(9)
622(1)	1139(1)	3221(15)	622(0)	1134(1)	3195(61)
715(48)	1147(0)	3224(14)	705(36)	1141(0)	3196(50)
741(9)	1153(11)	3227(2)	727(6)	1151(8)	3202(9)
772(0)	1159(1)	3239(7)	753(0)	1159(1)	3211(37)
790(6)	1321(0)	3242(0)	771(10)	1319(0)	3214(4)

Table S21. Harmonic vibrational frequencies (in cm^{-1}) and infrared intensities (in parentheses, in km/mol) for the $C_5H_5VC_7H_7$ structure **V5-57X**

B3PW91			M06-L		
15(0)	843(0)	1373(0)	8(0)	824(0)	1371(0)
61(0)	843(1)	1407(0)	66(0)	830(0)	1408(0)
73(0)	848(0)	1410(0)	81(0)	831(0)	1410(0)
85(0)	850(5)	1438(5)	89(0)	847(5)	1439(2)
137(0)	874(0)	1455(2)	144(1)	855(1)	1456(0)
218(4)	880(4)	1460(1)	219(2)	857(0)	1456(0)
291(0)	882(0)	1460(0)	286(0)	867(0)	1457(0)
304(4)	882(2)	1465(5)	308(2)	875(10)	1484(3)
330(1)	894(0)	1522(0)	351(1)	890(1)	1528(0)
343(2)	908(0)	1528(2)	365(3)	895(0)	1530(1)
403(12)	918(0)	1588(3)	402(8)	910(0)	1595(3)
415(1)	942(8)	3172(3)	411(1)	943(6)	3143(8)
436(13)	985(4)	3172(2)	438(12)	986(4)	3144(3)
515(1)	1022(19)	3183(0)	508(0)	1021(15)	3157(0)
550(0)	1023(19)	3185(0)	553(0)	1022(15)	3159(0)
607(0)	1069(0)	3194(28)	597(0)	1069(0)	3168(73)
611(0)	1071(0)	3200(21)	602(0)	1070(0)	3175(54)
651(120)	1140(8)	3207(9)	640(91)	1136(17)	3183(26)
753(4)	1163(1)	3239(0)	737(4)	1164(1)	3213(0)
762(24)	1172(0)	3241(0)	741(11)	1174(0)	3214(0)
788(11)	1244(0)	3255(3)	765(1)	1241(0)	3231(23)
788(1)	1259(0)	3255(3)	765(1)	1254(0)	3233(23)
791(93)	1265(0)	3268(0)	778(70)	1256(0)	3245(2)

Table S22. Harmonic vibrational frequencies (in cm^{-1}) and infrared intensities (in parentheses, in km/mol) for the $C_6H_6VC_6H_6$ structure **V6-66X**

B3PW91			M06-L		
-4(0)	817(1)	1338(0)	10(0)	803(0)	1335(0)
41(1)	824(0)	1385(0)	56(2)	806(1)	1399(2)
62(0)	839(7)	1392(11)	62(0)	839(3)	1409(4)
81(1)	884(1)	1457(1)	99(3)	846(1)	1446(0)
95(0)	888(0)	1459(5)	100(4)	865(1)	1460(2)
176(6)	931(7)	1463(5)	174(10)	871(3)	1462(1)
212(0)	934(7)	1471(2)	201(0)	895(0)	1464(1)
239(2)	946(0)	1508(1)	283(3)	912(0)	1517(8)
331(6)	968(44)	1516(0)	345(4)	967(73)	1522(0)
390(1)	981(27)	1543(33)	360(0)	983(23)	1529(0)
393(0)	991(2)	1563(10)	397(0)	994(12)	1536(1)
426(1)	998(1)	3180(1)	404(3)	1002(0)	3156(0)
428(1)	1018(10)	3186(5)	476(2)	1006(0)	3164(9)
573(0)	1020(11)	3191(2)	572(0)	1022(9)	3169(1)
590(0)	1025(10)	3203(10)	583(0)	1024(6)	3180(45)
596(5)	1029(6)	3208(25)	595(0)	1036(7)	3186(0)
599(0)	1139(5)	3213(0)	600(0)	1135(0)	3186(56)
603(3)	1145(0)	3217(0)	601(1)	1146(1)	3191(0)
651(1)	1147(0)	3218(0)	633(1)	1149(0)	3193(1)
677(84)	1156(0)	3222(10)	658(80)	1151(1)	3199(28)
704(38)	1161(1)	3230(7)	687(40)	1152(0)	3206(34)
783(44)	1166(0)	3232(6)	752(2)	1153(0)	3206(33)
786(2)	1337(0)	3239(0)	763(13)	1328(0)	3215(6)

Table S23. Harmonic vibrational frequencies (in cm^{-1}) and infrared intensities (in parentheses, in km/mol) for the $C_4H_4VC_8H_8$ structure **V7-48D**

B3PW91			M06-L		
14(0)	776(82)	1343(0)	-18(0)	769(89)	1340(0)
142(0)	790(81)	1359(0)	133(0)	791(52)	1365(0)
142(0)	828(14)	1359(0)	133(0)	821(12)	1365(0)
213(0)	828(14)	1400(0)	212(0)	821(12)	1400(0)
215(0)	884(0)	1457(0)	215(0)	870(0)	1459(1)
250(0)	890(0)	1457(0)	245(0)	874(0)	1459(1)
315(1)	927(0)	1548(0)	323(2)	925(0)	1546(0)
315(1)	938(1)	1549(0)	323(2)	932(0)	1547(0)
370(11)	938(1)	1572(1)	380(8)	936(0)	1572(1)
370(11)	942(0)	1572(1)	380(8)	936(0)	1572(1)
381(0)	945(28)	1589(0)	391(0)	942(2)	1608(0)
385(0)	945(28)	3171(0)	400(0)	942(2)	3141(0)
487(0)	953(0)	3176(0)	488(0)	950(22)	3146(0)
487(0)	953(0)	3176(0)	488(0)	950(22)	3146(0)
510(3)	963(0)	3186(0)	512(2)	970(0)	3155(0)
564(1)	980(0)	3187(0)	565(1)	975(0)	3156(0)
637(1)	1165(0)	3198(25)	598(2)	1155(0)	3166(88)
637(1)	1217(0)	3198(25)	598(2)	1217(0)	3166(88)
650(0)	1218(0)	3204(3)	634(0)	1218(0)	3172(9)
757(0)	1244(0)	3239(0)	746(0)	1263(0)	3195(0)
757(0)	1279(19)	3256(3)	760(3)	1284(24)	3217(26)
769(0)	1336(0)	3256(3)	764(0)	1332(0)	3217(26)
771(13)	1343(0)	3279(1)	764(0)	1340(0)	3242(1)

Table S24. Harmonic vibrational frequencies (in cm^{-1}) and infrared intensities (in parentheses, in km/mol) for the $C_4H_4VC_8H_8$ structure **V8-48Q**

B3PW91			M06-L		
46(3)	764(28)	1294(0)	47(12)	747(18)	1293(2)
71(0)	768(67)	1339(0)	62(0)	761(8)	1342(0)
88(0)	805(6)	1340(6)	99(0)	803(4)	1364(9)
133(6)	815(3)	1379(0)	127(15)	812(4)	1379(0)
153(7)	862(17)	1421(9)	167(0)	850(34)	1425(2)
157(0)	894(0)	1430(2)	175(1)	852(4)	1432(0)
240(3)	911(0)	1481(0)	237(26)	904(0)	1484(0)
252(0)	931(19)	1533(5)	252(0)	918(14)	1544(60)
314(10)	931(25)	1553(15)	318(9)	920(7)	1546(7)
323(8)	932(1)	1582(61)	348(0)	925(0)	1576(234)
323(2)	941(5)	1694(51)	358(2)	927(4)	1682(117)
333(2)	942(2)	3151(1)	361(58)	939(1)	3125(3)
408(8)	946(11)	3162(5)	406(21)	945(25)	3130(8)
450(1)	956(1)	3164(0)	431(100)	946(10)	3131(1)
481(1)	961(4)	3172(10)	459(0)	952(6)	3143(18)
561(0)	964(0)	3176(6)	559(0)	961(1)	3147(20)
586(1)	1102(0)	3182(11)	564(6)	1106(3)	3154(63)
601(1)	1160(0)	3186(14)	596(111)	1118(5)	3154(54)
640(4)	1217(1)	3198(9)	602(1)	1153(0)	3166(39)
655(29)	1223(2)	3229(0)	656(27)	1216(1)	3190(4)
699(7)	1259(0)	3245(4)	703(7)	1257(0)	3204(31)
733(98)	1265(17)	3247(3)	717(43)	1264(6)	3220(18)
756(2)	1288(1)	3269(0)	740(58)	1291(0)	3237(21)

Table S25. Harmonic vibrational frequencies (in cm^{-1}) and infrared intensities (in parentheses, in km/mol) for the $C_4H_4VC_8H_8$ structure **V9-48X**

B3PW91			M06-L		
17(0)	716(13)	1294(0)	-13(0)	705(3)	1288(0)
58(0)	772(1)	1299(2)	55(0)	753(1)	1295(1)
76(0)	779(7)	1360(1)	70(2)	778(2)	1367(1)
89(1)	790(4)	1383(1)	84(0)	783(2)	1378(1)
122(1)	799(9)	1422(7)	123(2)	791(11)	1421(2)
192(3)	853(1)	1438(4)	198(2)	838(3)	1439(1)
265(1)	885(3)	1486(0)	252(1)	874(4)	1484(0)
267(1)	896(4)	1520(14)	267(1)	900(0)	1528(12)
279(0)	905(1)	1568(11)	275(0)	905(0)	1563(13)
323(1)	926(3)	1595(14)	319(0)	908(6)	1592(14)
326(11)	929(3)	1655(8)	368(11)	915(6)	1654(7)
362(0)	936(30)	3141(1)	376(0)	924(4)	3113(2)
400(13)	940(9)	3143(3)	391(9)	935(3)	3114(6)
420(11)	947(3)	3156(5)	429(10)	941(21)	3130(23)
467(4)	950(11)	3161(4)	459(4)	948(10)	3134(7)
539(1)	958(0)	3167(13)	527(1)	954(0)	3142(2)
545(22)	1039(0)	3173(31)	536(13)	1035(0)	3146(85)
585(30)	1120(2)	3184(42)	572(28)	1118(2)	3159(67)
631(0)	1160(0)	3189(5)	653(0)	1149(0)	3164(44)
669(64)	1212(5)	3228(0)	666(50)	1206(5)	3176(1)
677(0)	1238(27)	3235(4)	694(3)	1239(40)	3182(28)
703(6)	1274(21)	3264(0)	695(36)	1267(1)	3230(10)
707(53)	1275(1)	3275(1)	697(5)	1273(19)	3242(3)

Table S26. Harmonic vibrational frequencies (in cm^{-1}) and infrared intensities (in parentheses, in km/mol) for the $C_6H_6CrC_6H_6$ structure **Cr1-66S**

B3PW91			M06-L		
40(0)	872(9)	1323(0)	35(0)	837(0)	1320(0)
164(1)	872(9)	1451(4)	154(1)	837(0)	1449(1)
164(1)	873(0)	1451(4)	154(1)	861(7)	1449(1)
272(0)	873(0)	1453(0)	273(0)	861(7)	1452(0)
334(0)	910(0)	1453(0)	350(0)	891(0)	1452(0)
334(0)	910(0)	1453(0)	350(0)	891(0)	1465(0)
390(0)	921(0)	1457(0)	379(0)	892(0)	1473(0)
390(0)	928(0)	1516(0)	379(0)	905(0)	1511(0)
450(0)	995(0)	1516(0)	452(0)	998(0)	1511(0)
450(0)	997(22)	1557(0)	452(0)	1001(0)	1560(0)
464(191)	1001(0)	1557(0)	463(173)	1001(29)	1560(0)
496(47)	1013(0)	3193(0)	513(49)	1012(0)	3149(0)
496(47)	1019(0)	3195(0)	513(49)	1022(0)	3149(0)
619(0)	1019(0)	3197(0)	621(0)	1022(0)	3156(0)
619(0)	1022(12)	3197(0)	621(0)	1026(7)	3156(0)
626(0)	1022(12)	3200(0)	621(0)	1026(7)	3156(0)
630(0)	1138(0)	3200(0)	623(0)	1136(0)	3156(0)
630(0)	1138(0)	3209(0)	635(0)	1136(0)	3166(0)
632(0)	1147(0)	3209(0)	635(0)	1140(0)	3166(0)
801(0)	1152(0)	3212(60)	791(0)	1144(0)	3167(204)
805(55)	1152(0)	3212(60)	804(28)	1150(0)	3167(204)
849(0)	1153(0)	3217(11)	830(0)	1150(0)	3170(49)
849(0)	1321(0)	3219(0)	830(0)	1317(0)	3175(0)

Table S27. Harmonic vibrational frequencies (in cm^{-1}) and infrared intensities (in parentheses, in km/mol) for the $C_5H_5CrC_7H_7$ structure **Cr2-57S**

B3PW91				M06-L		
6(0)	868(15)	1372(0)		-2(0)	861(13)	1368(0)
167(0)	868(16)	1423(0)		157(0)	862(13)	1425(0)
168(0)	888(7)	1423(0)		159(0)	873(0)	1425(0)
268(2)	891(0)	1456(0)		269(1)	875(0)	1454(1)
315(0)	891(0)	1457(0)		312(0)	890(0)	1455(1)
315(0)	894(0)	1461(1)		313(0)	890(0)	1459(0)
323(0)	895(0)	1461(1)		338(1)	891(9)	1459(0)
323(0)	910(0)	1556(0)		339(1)	895(0)	1550(0)
461(34)	910(0)	1556(0)		473(24)	896(0)	1552(0)
462(34)	947(0)	1558(0)		478(36)	927(0)	1583(0)
472(25)	948(0)	1558(0)		478(36)	928(0)	1583(0)
491(0)	993(6)	3168(0)		499(0)	996(4)	3123(1)
491(0)	994(5)	3168(0)		499(0)	996(4)	3123(0)
536(0)	1025(10)	3177(0)		526(0)	1027(7)	3132(0)
536(0)	1026(11)	3177(0)		527(0)	1027(7)	3133(0)
605(0)	1069(0)	3187(43)		608(0)	1067(0)	3138(145)
606(0)	1069(0)	3188(42)		608(0)	1068(0)	3140(142)
812(48)	1147(9)	3194(3)		802(8)	1147(14)	3146(12)
816(1)	1199(0)	3237(0)		803(1)	1194(0)	3198(0)
817(2)	1199(0)	3237(0)		807(16)	1195(0)	3198(0)
832(0)	1259(0)	3252(8)		813(0)	1250(0)	3216(41)
832(0)	1260(0)	3252(8)		813(0)	1251(0)	3217(41)
837(82)	1265(0)	3264(2)		836(56)	1257(0)	3227(13)

Table S28. Harmonic vibrational frequencies (in cm^{-1}) and infrared intensities (in parentheses, in km/mol) for the $C_5H_5CrC_7H_7$ structure **Cr3-57T**

B3PW91			M06-L		
37(0)	824(4)	1337(0)	24(0)	809(1)	1340(0)
90(1)	828(0)	1395(10)	76(1)	812(4)	1404(3)
130(0)	843(0)	1398(5)	121(0)	834(0)	1407(2)
153(6)	847(0)	1410(1)	123(4)	838(0)	1414(1)
251(1)	887(0)	1414(1)	246(0)	866(0)	1416(0)
302(0)	900(0)	1448(1)	301(0)	874(0)	1456(1)
310(0)	904(11)	1460(1)	311(1)	887(18)	1459(2)
326(0)	904(25)	1466(2)	330(0)	901(5)	1466(1)
367(4)	926(2)	1490(18)	366(3)	908(8)	1492(11)
384(9)	935(2)	1509(0)	386(8)	916(4)	1521(0)
431(2)	955(2)	1689(11)	427(1)	938(4)	1665(6)
461(7)	963(6)	3151(0)	456(8)	943(7)	3126(0)
482(7)	998(4)	3160(5)	496(4)	996(6)	3135(7)
567(5)	1020(16)	3163(21)	555(2)	1020(14)	3139(42)
584(1)	1028(16)	3177(29)	570(1)	1030(14)	3150(16)
603(0)	1071(0)	3181(19)	598(0)	1072(0)	3157(82)
609(0)	1072(0)	3186(20)	605(0)	1072(0)	3160(55)
725(43)	1120(1)	3202(10)	714(36)	1129(1)	3173(28)
771(8)	1143(9)	3245(0)	759(6)	1143(14)	3218(0)
792(59)	1159(6)	3253(0)	774(1)	1167(3)	3224(2)
800(24)	1195(1)	3264(1)	777(41)	1202(1)	3239(15)
804(29)	1213(0)	3265(1)	790(44)	1213(0)	3240(17)
814(4)	1266(0)	3279(0)	792(3)	1258(0)	3253(3)

Table S29. Harmonic vibrational frequencies (in cm^{-1}) and infrared intensities (in parentheses, in km/mol) for the $C_6H_6CrC_6H_6$ structure **Cr4-66T**

B3PW91			M06-L		
16(0)	806(1)	1324(0)	40(0)	777(0)	1320(0)
88(0)	826(5)	1396(1)	82(0)	792(2)	1411(0)
96(0)	839(0)	1400(0)	92(0)	814(0)	1413(0)
122(0)	849(1)	1438(6)	106(0)	816(2)	1435(0)
125(0)	876(2)	1439(0)	214(0)	845(2)	1445(1)
220(0)	884(0)	1458(1)	221(1)	859(0)	1452(0)
226(0)	913(1)	1458(3)	251(1)	885(1)	1465(0)
262(0)	913(1)	1465(0)	273(6)	892(2)	1469(0)
315(3)	977(0)	1494(0)	297(1)	974(38)	1488(1)
338(100)	977(53)	1537(0)	355(56)	978(28)	1540(2)
362(0)	989(2)	1557(0)	375(22)	981(10)	1562(2)
424(1)	994(24)	3212(0)	429(0)	994(15)	3177(0)
429(0)	1005(2)	3213(0)	437(2)	1007(1)	3183(0)
563(0)	1008(7)	3215(0)	537(3)	1007(6)	3187(1)
590(2)	1026(0)	3216(0)	577(6)	1028(1)	3188(0)
595(7)	1027(21)	3220(0)	595(0)	1030(16)	3190(7)
601(0)	1122(0)	3220(0)	595(2)	1126(0)	3192(3)
605(1)	1137(0)	3230(2)	603(1)	1133(0)	3201(30)
610(0)	1144(0)	3230(14)	609(0)	1136(0)	3203(55)
719(122)	1145(0)	3233(7)	703(82)	1138(0)	3204(44)
735(0)	1147(0)	3233(19)	719(12)	1147(0)	3206(41)
786(0)	1153(0)	3240(1)	765(0)	1152(0)	3212(7)
793(2)	1324(0)	3240(0)	773(2)	1319(0)	3214(9)

Table S30. Harmonic vibrational frequencies (in cm^{-1}) and infrared intensities (in parentheses, in km/mol) for the $C_6H_6CrC_6H_6$ structure **Cr5-66P**

B3PW91			M06-L		
30(0)	806(101)	1343(0)	23(0)	778(35)	1336(0)
46(0)	819(0)	1367(28)	54(0)	798(0)	1381(19)
52(0)	824(1)	1383(6)	56(0)	811(0)	1400(0)
89(6)	881(6)	1389(1)	75(0)	866(2)	1403(1)
101(1)	914(0)	1431(1)	122(5)	891(0)	1435(0)
207(3)	934(1)	1468(5)	206(18)	897(5)	1461(1)
219(18)	942(50)	1474(20)	239(11)	916(0)	1472(8)
225(1)	960(45)	1485(2)	247(0)	930(0)	1482(3)
315(20)	965(0)	1563(99)	302(27)	967(78)	1560(1)
328(5)	966(28)	1575(13)	325(0)	976(22)	1562(33)
367(1)	977(21)	1594(16)	352(0)	980(22)	1568(39)
408(11)	995(1)	3191(1)	375(1)	1005(0)	3167(2)
433(0)	1002(3)	3197(4)	459(6)	1009(2)	3172(3)
578(0)	1025(3)	3200(1)	581(0)	1018(8)	3177(5)
588(1)	1031(8)	3201(1)	591(4)	1028(4)	3178(1)
596(1)	1034(7)	3202(1)	592(1)	1038(7)	3179(1)
597(4)	1058(0)	3213(18)	594(0)	1073(0)	3191(50)
604(1)	1145(1)	3215(5)	603(0)	1139(0)	3192(9)
670(0)	1152(7)	3216(15)	651(1)	1144(0)	3193(37)
682(38)	1162(8)	3219(18)	670(37)	1157(0)	3196(57)
707(3)	1166(2)	3227(6)	696(0)	1161(6)	3206(21)
734(7)	1175(1)	3234(8)	730(3)	1167(1)	3211(35)
784(80)	1323(0)	3238(0)	769(96)	1321(0)	3216(7)

Table S31. Harmonic vibrational frequencies (in cm^{-1}) and infrared intensities (in parentheses, in km/mol) for the $C_5H_5CrC_7H_7$ structure **Cr6-57P**

B3PW91			M06-L		
4(0)	819(1)	1290(6)	-15(0)	805(0)	1291(3)
47(0)	842(0)	1337(3)	53(0)	837(0)	1334(2)
90(0)	846(1)	1388(16)	87(0)	839(0)	1388(10)
134(5)	870(1)	1402(0)	142(3)	863(1)	1407(0)
161(1)	880(0)	1410(2)	164(0)	864(0)	1413(1)
208(1)	881(1)	1445(1)	219(4)	869(0)	1444(2)
245(1)	912(1)	1456(1)	268(2)	907(1)	1458(0)
260(3)	931(9)	1473(0)	274(2)	921(14)	1472(0)
275(5)	948(3)	1542(9)	285(2)	928(4)	1524(13)
326(1)	976(1)	1606(0)	329(0)	944(0)	1604(0)
376(38)	977(2)	1637(5)	375(34)	965(1)	1634(3)
407(18)	980(1)	3115(17)	412(6)	984(1)	3087(25)
411(6)	1018(17)	3145(19)	415(10)	1020(10)	3110(34)
493(28)	1030(21)	3149(33)	483(24)	1025(11)	3116(74)
593(4)	1035(13)	3155(0)	578(9)	1031(17)	3126(0)
598(6)	1055(8)	3171(23)	591(1)	1055(6)	3142(52)
611(0)	1069(0)	3192(24)	600(1)	1070(0)	3164(63)
723(28)	1069(0)	3193(11)	716(27)	1071(0)	3166(36)
734(58)	1145(1)	3236(0)	730(39)	1146(5)	3213(1)
750(9)	1172(1)	3241(0)	741(8)	1167(2)	3218(0)
761(0)	1175(0)	3253(4)	745(0)	1169(1)	3231(23)
782(147)	1212(1)	3256(2)	777(104)	1206(2)	3237(19)
798(5)	1265(0)	3268(0)	783(4)	1259(0)	3249(4)

Table S32. Harmonic vibrational frequencies (in cm^{-1}) and infrared intensities (in parentheses, in km/mol) for the $C_4H_4CrC_8H_8$ structure **Cr7-48T**

B3PW91			M06-L		
33(0)	774(1)	1308(0)	23(0)	767(3)	1313(0)
80(2)	780(33)	1351(0)	73(1)	777(68)	1366(0)
127(0)	817(10)	1362(0)	123(0)	808(10)	1368(0)
128(11)	821(15)	1381(0)	128(7)	808(7)	1385(0)
173(0)	869(8)	1427(5)	178(0)	851(5)	1436(0)
182(2)	885(1)	1435(1)	193(2)	866(2)	1440(0)
266(2)	912(6)	1495(0)	263(2)	910(9)	1503(0)
274(0)	936(0)	1523(0)	270(0)	926(0)	1521(1)
332(4)	939(17)	1557(6)	329(2)	928(1)	1554(4)
354(0)	939(2)	1587(50)	374(0)	934(0)	1589(12)
374(3)	945(24)	1675(24)	395(6)	946(2)	1647(11)
391(3)	948(4)	3151(0)	418(5)	947(12)	3129(0)
433(4)	959(3)	3161(3)	440(5)	950(7)	3135(4)
490(0)	964(2)	3164(2)	487(0)	951(27)	3140(6)
491(13)	964(1)	3174(19)	501(4)	967(2)	3151(20)
574(0)	979(1)	3178(12)	552(0)	971(0)	3152(27)
631(0)	1136(0)	3183(13)	599(3)	1152(0)	3160(58)
646(2)	1167(0)	3191(17)	628(1)	1157(0)	3164(57)
654(0)	1212(2)	3204(9)	634(1)	1214(1)	3176(29)
670(15)	1237(2)	3243(0)	673(19)	1257(0)	3200(1)
726(7)	1267(0)	3261(2)	729(7)	1280(2)	3220(19)
762(21)	1274(22)	3261(2)	753(17)	1282(22)	3223(16)
773(70)	1306(1)	3282(1)	760(27)	1306(0)	3246(1)

Table S33. Harmonic vibrational frequencies (in cm^{-1}) and infrared intensities (in parentheses, in km/mol) for the $C_4H_4CrC_8H_8$ structure **Cr8-48S**

B3PW91			M06-L		
16(0)	796(12)	1352(0)	-23(0)	795(4)	1344(0)
164(0)	809(83)	1359(0)	145(0)	806(56)	1364(0)
164(0)	863(15)	1359(0)	150(0)	859(12)	1364(0)
167(0)	863(15)	1409(0)	150(0)	859(12)	1409(0)
183(0)	890(0)	1469(0)	185(0)	864(0)	1469(0)
265(2)	908(0)	1469(0)	253(2)	900(0)	1469(0)
316(4)	909(0)	1558(0)	319(6)	915(0)	1555(0)
316(4)	944(0)	1576(0)	319(6)	934(0)	1571(0)
370(23)	946(5)	1587(5)	370(21)	936(1)	1603(0)
370(23)	946(5)	1607(0)	370(21)	936(1)	1603(0)
371(0)	951(12)	1607(0)	383(0)	950(0)	1614(3)
374(0)	951(12)	3150(0)	384(0)	950(0)	3108(0)
456(0)	961(0)	3156(0)	454(0)	955(11)	3114(0)
456(0)	961(0)	3156(0)	454(0)	955(11)	3114(0)
523(10)	964(0)	3166(0)	522(7)	971(0)	3122(0)
556(0)	991(0)	3166(0)	560(0)	981(0)	3122(0)
650(0)	1166(0)	3177(49)	640(0)	1156(0)	3127(170)
684(4)	1222(0)	3177(49)	655(8)	1221(0)	3127(170)
684(4)	1234(0)	3182(7)	655(8)	1233(0)	3132(23)
745(0)	1248(0)	3229(0)	703(0)	1266(0)	3176(0)
745(0)	1280(22)	3248(9)	749(0)	1284(27)	3200(41)
763(0)	1348(0)	3248(9)	749(0)	1340(0)	3200(41)
786(30)	1352(0)	3270(0)	769(28)	1344(0)	3223(8)

Table S34. Harmonic vibrational frequencies (in cm^{-1}) and infrared intensities (in parentheses, in km/mol) for the $C_4H_4CrC_8H_8$ structure **Cr9-48P**

B3PW91				M06-L		
11(0)	770(94)	1304(3)		16(0)	760(75)	1306(2)
54(1)	778(14)	1348(1)		49(0)	777(5)	1360(1)
88(0)	792(3)	1355(0)		89(0)	789(2)	1363(0)
107(1)	804(11)	1386(1)		103(1)	796(6)	1384(1)
120(0)	860(2)	1425(9)		127(0)	846(3)	1427(3)
192(5)	891(3)	1442(4)		193(4)	882(2)	1445(1)
269(1)	908(0)	1489(0)		269(0)	905(0)	1491(0)
271(4)	929(10)	1521(15)		271(1)	911(6)	1532(15)
282(1)	932(4)	1564(8)		279(1)	921(7)	1559(8)
324(0)	935(26)	1596(15)		322(0)	930(1)	1595(11)
358(7)	938(18)	1643(4)		381(8)	943(6)	1638(6)
360(4)	946(1)	3149(2)		388(3)	945(18)	3122(4)
402(9)	951(1)	3152(2)		399(7)	946(16)	3124(4)
472(4)	953(13)	3164(0)		471(4)	950(12)	3138(8)
481(1)	958(3)	3171(1)		488(1)	956(0)	3143(2)
538(0)	962(0)	3172(10)		536(0)	965(2)	3147(11)
614(0)	1125(2)	3181(24)		589(0)	1129(1)	3154(72)
619(2)	1164(0)	3188(33)		590(1)	1155(0)	3166(77)
627(0)	1214(6)	3194(5)		617(2)	1211(5)	3169(23)
673(63)	1221(0)	3236(0)		670(55)	1226(8)	3198(0)
707(6)	1271(20)	3250(1)		702(8)	1277(19)	3217(15)
710(57)	1280(1)	3257(1)		705(38)	1277(8)	3222(17)
763(0)	1298(0)	3276(1)		744(3)	1294(0)	3244(1)

Table S35. Harmonic vibrational frequencies (in cm^{-1}) and infrared intensities (in parentheses, in km/mol) for the $C_6H_6MnC_6H_6$ structure **Mn1-66Q**

B3PW91			M06-L		
31(0)	804(1)	1329(0)	24(0)	784(1)	1328(0)
78(0)	804(1)	1370(0)	44(0)	784(1)	1385(0)
78(0)	864(0)	1378(0)	44(0)	837(0)	1396(0)
88(0)	864(0)	1453(5)	61(0)	837(0)	1454(1)
88(0)	864(0)	1453(5)	61(0)	840(0)	1454(1)
192(0)	864(0)	1453(0)	169(0)	840(0)	1454(0)
220(0)	916(0)	1453(0)	169(0)	896(0)	1454(0)
220(0)	918(0)	1476(0)	173(60)	898(0)	1482(0)
232(78)	975(85)	1476(0)	182(0)	974(97)	1482(0)
370(0)	979(0)	1553(0)	367(0)	981(0)	1560(0)
370(0)	998(0)	1553(0)	367(0)	1002(0)	1560(0)
407(0)	1005(0)	3212(0)	408(0)	1006(0)	3185(0)
407(0)	1015(0)	3213(0)	408(0)	1016(0)	3186(0)
593(0)	1015(0)	3217(0)	577(0)	1016(0)	3192(0)
596(0)	1016(29)	3217(0)	589(0)	1017(27)	3192(0)
596(0)	1016(29)	3219(0)	593(0)	1017(26)	3193(0)
605(0)	1126(0)	3219(0)	593(0)	1129(0)	3193(0)
605(0)	1126(0)	3231(0)	600(0)	1129(0)	3207(0)
617(0)	1147(0)	3231(0)	600(0)	1142(0)	3207(0)
693(132)	1149(0)	3233(19)	679(111)	1144(0)	3207(81)
713(0)	1158(0)	3233(19)	696(0)	1160(0)	3207(80)
795(0)	1158(0)	3241(0)	777(0)	1160(0)	3217(10)
795(0)	1329(0)	3242(0)	777(0)	1327(0)	3218(0)

Table S36. Harmonic vibrational frequencies (in cm^{-1}) and infrared intensities (in parentheses, in km/mol) for the $C_6H_6MnC_6H_6$ structure **Mn2-66D**

B3PW91			M06-L		
17(0)	859(0)	1328(0)	25(0)	834(0)	1325(0)
112(0)	872(4)	1413(10)	74(0)	846(0)	1428(13)
112(0)	881(7)	1422(0)	132(0)	854(4)	1438(0)
141(1)	892(0)	1448(11)	134(1)	866(7)	1448(3)
148(0)	900(0)	1450(0)	159(0)	877(0)	1450(0)
233(0)	918(0)	1464(4)	182(1)	898(0)	1464(2)
243(0)	934(0)	1466(0)	230(0)	907(1)	1465(0)
276(2)	935(0)	1521(4)	244(0)	911(0)	1510(0)
325(7)	991(0)	1535(0)	262(1)	986(10)	1535(1)
346(72)	993(0)	1550(0)	357(95)	989(0)	1547(2)
381(55)	995(22)	1553(0)	359(0)	995(0)	1570(1)
393(0)	998(9)	3194(0)	393(18)	995(32)	3154(14)
423(0)	1010(0)	3195(7)	444(0)	1011(0)	3154(0)
543(0)	1013(14)	3197(0)	520(7)	1015(9)	3159(1)
588(0)	1032(0)	3198(0)	571(0)	1034(0)	3160(1)
604(0)	1034(19)	3201(1)	592(11)	1035(18)	3164(0)
604(13)	1137(0)	3204(0)	600(0)	1138(0)	3165(10)
606(0)	1143(0)	3209(0)	603(0)	1143(0)	3172(0)
613(0)	1148(0)	3211(52)	618(0)	1144(0)	3173(138)
754(91)	1149(0)	3217(0)	746(58)	1145(1)	3175(11)
767(0)	1153(1)	3219(36)	755(0)	1150(0)	3177(145)
815(0)	1159(0)	3223(8)	809(1)	1154(0)	3183(29)
831(2)	1327(0)	3225(0)	822(0)	1324(0)	3186(14)

Table S37. Harmonic vibrational frequencies (in cm^{-1}) and infrared intensities (in parentheses, in km/mol) for the $C_5H_5MnC_7H_7$ structure **Mn3-57Q**

B3PW91			M06-L		
7(0)	846(0)	1372(0)	20(0)	836(0)	1368(0)
73(0)	846(0)	1403(0)	52(2)	836(0)	1408(0)
74(0)	854(0)	1403(0)	53(2)	839(0)	1409(0)
92(1)	855(0)	1446(4)	68(1)	839(0)	1445(1)
93(1)	858(8)	1446(4)	71(1)	846(0)	1446(1)
174(0)	866(0)	1461(0)	179(0)	847(0)	1464(0)
189(0)	866(0)	1462(0)	189(1)	861(9)	1465(0)
190(0)	886(0)	1517(0)	189(1)	885(0)	1526(0)
278(0)	886(0)	1517(0)	285(0)	885(0)	1526(0)
278(0)	917(0)	1525(0)	286(0)	906(0)	1527(0)
304(37)	917(0)	1525(0)	296(30)	906(0)	1528(0)
441(0)	968(9)	3197(0)	449(0)	971(9)	3170(0)
441(0)	968(9)	3197(0)	449(0)	972(9)	3170(0)
479(0)	1022(23)	3204(0)	461(0)	1024(19)	3179(0)
480(0)	1022(23)	3204(0)	462(0)	1024(20)	3179(0)
617(0)	1067(0)	3216(9)	610(0)	1068(0)	3190(42)
617(0)	1067(0)	3216(9)	611(0)	1069(0)	3190(42)
707(190)	1146(0)	3224(0)	707(147)	1148(0)	3198(3)
746(0)	1181(0)	3231(0)	722(1)	1182(0)	3202(0)
747(0)	1181(0)	3231(0)	723(1)	1182(0)	3202(0)
768(157)	1254(0)	3247(7)	760(129)	1248(0)	3222(35)
783(4)	1255(0)	3248(7)	774(3)	1248(0)	3223(35)
784(4)	1262(0)	3262(1)	775(3)	1256(0)	3238(4)

Table S38. Harmonic vibrational frequencies (in cm^{-1}) and infrared intensities (in parentheses, in km/mol) for the $C_5H_5MnC_7H_7$ structure **Mn4-57X**

B3PW91				M06-L		
-7(0)	816(5)	1316(12)		-18(0)	812(4)	1319(10)
26(0)	848(0)	1351(4)		24(0)	844(0)	1350(3)
50(1)	848(0)	1403(0)		60(0)	844(0)	1407(0)
63(8)	874(0)	1403(10)		82(6)	860(0)	1407(9)
92(2)	877(0)	1404(2)		95(2)	861(0)	1410(0)
167(1)	883(0)	1460(1)		158(0)	891(0)	1460(3)
205(2)	910(4)	1462(0)		221(1)	902(3)	1464(0)
208(0)	945(1)	1463(0)		229(2)	942(1)	1465(0)
231(6)	960(1)	1606(9)		232(2)	948(1)	1610(9)
290(18)	972(1)	1619(2)		289(19)	964(1)	1612(4)
373(46)	974(1)	1661(8)		369(36)	969(0)	1660(6)
393(0)	987(0)	3101(15)		387(0)	976(0)	3087(20)
397(1)	1024(22)	3147(15)		399(0)	1027(18)	3116(33)
496(59)	1024(19)	3152(25)		493(61)	1027(10)	3122(62)
610(2)	1044(23)	3153(1)		604(1)	1033(22)	3123(0)
610(0)	1071(0)	3171(33)		605(1)	1072(0)	3143(65)
614(0)	1071(0)	3191(31)		608(0)	1073(0)	3169(67)
731(102)	1079(11)	3193(9)		727(14)	1081(10)	3171(21)
732(17)	1145(1)	3237(0)		728(83)	1147(0)	3213(0)
761(4)	1197(6)	3238(0)		752(1)	1197(5)	3215(0)
770(0)	1204(0)	3253(2)		755(0)	1204(0)	3232(20)
771(3)	1227(0)	3254(2)		765(7)	1225(0)	3233(19)
784(209)	1265(0)	3266(0)		782(162)	1260(0)	3247(3)

Table S39. Harmonic vibrational frequencies (in cm^{-1}) and infrared intensities (in parentheses, in km/mol) for the $C_5H_5MnC_7H_7$ structure **Mn5-57D**

B3PW91				M06-L		
46(0)	823(6)	1328(0)		26(0)	825(3)	1335(0)
113(2)	828(11)	1387(12)		97(4)	832(1)	1393(7)
146(0)	840(0)	1404(1)		135(3)	851(8)	1404(1)
250(1)	863(3)	1408(0)		149(0)	875(0)	1414(1)
300(0)	888(1)	1420(4)		269(2)	880(0)	1418(0)
300(2)	897(3)	1440(2)		301(0)	886(2)	1458(0)
331(1)	904(8)	1445(2)		336(1)	904(3)	1459(0)
350(0)	923(22)	1467(1)		350(0)	923(4)	1465(2)
408(6)	945(0)	1488(2)		379(3)	932(27)	1510(14)
435(10)	958(9)	1490(0)		412(13)	936(0)	1519(0)
450(2)	962(1)	1705(4)		431(6)	961(3)	1704(15)
505(9)	978(6)	3149(2)		480(19)	972(4)	3096(41)
513(35)	1009(7)	3154(5)		516(16)	1011(2)	3097(17)
582(8)	1010(15)	3160(23)		574(6)	1024(10)	3116(9)
591(1)	1029(15)	3178(22)		594(2)	1026(8)	3120(29)
598(4)	1066(0)	3182(26)		597(0)	1069(0)	3124(96)
601(0)	1075(1)	3182(19)		603(0)	1070(0)	3143(78)
728(43)	1111(1)	3210(10)		730(31)	1125(0)	3149(89)
779(11)	1142(18)	3251(1)		783(5)	1144(14)	3206(1)
798(40)	1161(1)	3254(0)		800(11)	1151(7)	3213(2)
803(2)	1187(3)	3268(1)		808(5)	1187(1)	3227(27)
804(0)	1200(0)	3269(2)		808(27)	1203(0)	3228(27)
818(27)	1263(0)	3282(1)		822(1)	1258(0)	3240(9)

Table S40. Harmonic vibrational frequencies (in cm^{-1}) and infrared intensities (in parentheses, in km/mol) for the $C_6H_6MnC_6H_6$ structure **Mn6-66X**

B3PW91			M06-L		
24(0)	783(1)	1333(0)	28(1)	801(7)	1331(0)
46(0)	799(15)	1348(28)	37(0)	808(0)	1358(68)
47(0)	830(2)	1359(22)	38(0)	815(18)	1363(0)
74(0)	834(107)	1404(0)	51(0)	823(0)	1428(0)
89(1)	917(7)	1419(0)	60(0)	912(160)	1436(2)
143(0)	918(0)	1445(3)	161(2)	926(0)	1464(14)
173(0)	939(44)	1449(0)	195(3)	954(0)	1468(0)
218(1)	939(0)	1487(1)	295(0)	956(2)	1545(306)
219(17)	953(3)	1505(1)	336(10)	957(2)	1563(0)
271(102)	960(114)	1593(271)	399(11)	957(0)	1577(20)
350(0)	973(12)	1618(1)	415(0)	978(0)	1581(0)
365(11)	983(17)	3188(0)	422(1)	984(8)	3164(0)
416(0)	1001(0)	3188(0)	429(0)	991(48)	3165(1)
543(19)	1008(4)	3199(7)	532(0)	993(0)	3177(32)
554(2)	1038(1)	3211(0)	578(2)	1023(0)	3177(0)
575(9)	1039(2)	3211(32)	594(0)	1029(7)	3191(0)
603(0)	1091(0)	3212(19)	596(3)	1126(104)	3191(6)
605(0)	1110(0)	3212(0)	657(1)	1131(0)	3196(12)
611(0)	1137(1)	3222(5)	661(0)	1167(16)	3196(0)
693(51)	1152(1)	3223(3)	701(57)	1168(0)	3211(0)
715(27)	1159(10)	3229(8)	706(0)	1169(0)	3211(49)
755(0)	1172(24)	3239(4)	755(0)	1170(0)	3224(23)
756(6)	1319(0)	3244(0)	773(272)	1328(0)	3224(0)

Table S41. Harmonic vibrational frequencies (in cm^{-1}) and infrared intensities (in parentheses, in km/mol) for the $C_4H_4MnC_8H_8$ structure **Mn7-48Q**

B3PW91			M06-L		
44(0)	772(0)	1324(1)	44(0)	760(49)	1322(1)
66(0)	785(19)	1348(1)	61(0)	788(16)	1357(0)
81(0)	801(3)	1356(0)	90(0)	793(3)	1365(0)
115(0)	816(9)	1387(0)	128(0)	806(5)	1386(0)
130(0)	822(3)	1433(2)	134(1)	807(3)	1437(0)
172(11)	877(2)	1441(0)	182(7)	865(3)	1452(1)
244(0)	890(10)	1451(0)	239(0)	892(10)	1477(0)
298(0)	920(0)	1509(4)	293(0)	907(1)	1509(3)
322(3)	935(0)	1524(1)	323(3)	919(2)	1527(1)
359(1)	940(10)	1564(1)	359(0)	927(0)	1564(0)
362(2)	940(11)	1568(4)	385(2)	935(0)	1567(4)
365(1)	941(1)	3149(1)	387(1)	946(17)	3125(3)
424(2)	944(18)	3152(0)	435(2)	951(12)	3127(2)
487(4)	955(6)	3165(1)	488(3)	959(7)	3140(4)
524(2)	963(6)	3170(26)	515(2)	965(7)	3145(53)
569(5)	974(1)	3182(3)	560(3)	974(1)	3157(2)
615(0)	1169(0)	3190(37)	617(0)	1159(0)	3166(48)
655(2)	1177(0)	3190(18)	624(3)	1178(0)	3169(79)
664(0)	1183(2)	3204(6)	632(0)	1183(2)	3180(21)
694(58)	1203(11)	3251(0)	684(45)	1227(8)	3213(0)
705(3)	1269(26)	3268(0)	698(4)	1275(32)	3234(9)
758(2)	1284(0)	3269(0)	749(19)	1282(0)	3235(10)
765(81)	1302(0)	3288(3)	750(0)	1296(0)	3257(0)

Table S42. Harmonic vibrational frequencies (in cm^{-1}) and infrared intensities (in parentheses, in km/mol) for the $C_4H_4MnC_8H_8$ structure **Mn8-48D**

B3PW91			M06-L		
45(0)	779(13)	1343(4)	62(0)	772(27)	1341(3)
141(0)	789(46)	1343(4)	136(0)	789(14)	1342(0)
141(0)	815(14)	1364(1)	136(0)	809(13)	1369(0)
173(0)	815(14)	1364(1)	164(0)	809(13)	1369(0)
192(0)	847(0)	1404(0)	197(0)	835(0)	1406(0)
200(0)	919(0)	1460(2)	202(0)	907(0)	1464(0)
200(0)	924(0)	1460(2)	202(0)	922(0)	1464(0)
215(0)	930(2)	1543(0)	231(0)	933(2)	1549(0)
330(0)	938(6)	1560(0)	332(0)	933(2)	1558(0)
339(0)	938(6)	1572(30)	356(0)	934(2)	1573(23)
399(8)	949(1)	1572(30)	433(1)	941(5)	1573(23)
399(8)	949(1)	3155(0)	433(1)	941(5)	3122(0)
443(4)	951(0)	3159(8)	445(12)	958(12)	3126(18)
443(4)	952(17)	3159(8)	445(12)	958(12)	3126(18)
492(31)	952(17)	3163(0)	513(27)	968(0)	3132(0)
566(0)	974(0)	3184(0)	572(1)	970(0)	3149(0)
629(0)	1015(0)	3191(31)	640(0)	1160(0)	3155(98)
704(0)	1171(0)	3191(31)	689(1)	1211(0)	3155(98)
704(0)	1208(0)	3196(3)	689(1)	1223(0)	3161(10)
740(1)	1220(0)	3239(0)	717(0)	1258(0)	3185(0)
740(1)	1224(0)	3258(3)	743(2)	1263(0)	3209(29)
755(50)	1280(19)	3258(3)	743(2)	1283(27)	3209(29)
772(0)	1341(0)	3278(0)	748(26)	1341(3)	3230(6)

Table S43. Harmonic vibrational frequencies (in cm^{-1}) and infrared intensities (in parentheses, in km/mol) for the $C_4H_4MnC_8H_8$ structure **Mn9-48X**

B3PW91			M06-L		
31(0)	771(3)	1295(3)	22(0)	756(20)	1300(2)
35(1)	776(3)	1301(0)	51(1)	773(3)	1301(14)
74(0)	784(18)	1387(1)	90(0)	776(2)	1387(0)
74(1)	801(8)	1425(5)	111(0)	796(5)	1430(1)
110(0)	843(7)	1437(5)	114(0)	828(12)	1441(3)
139(8)	876(7)	1463(76)	142(11)	880(4)	1449(46)
171(5)	896(4)	1484(1)	205(1)	892(0)	1489(0)
220(0)	908(0)	1508(6)	228(0)	904(11)	1518(5)
251(0)	918(3)	1559(14)	279(0)	905(0)	1555(15)
290(0)	921(2)	1593(12)	282(0)	915(2)	1593(15)
305(1)	924(2)	1646(53)	303(0)	916(3)	1643(61)
329(1)	945(15)	3151(0)	339(0)	936(9)	3125(4)
397(8)	949(51)	3155(1)	402(8)	947(2)	3127(3)
458(3)	955(0)	3161(0)	454(3)	954(72)	3138(0)
463(7)	959(2)	3170(5)	458(2)	955(0)	3146(14)
520(2)	983(38)	3171(2)	528(1)	990(11)	3147(3)
626(0)	1116(2)	3181(32)	598(29)	1124(1)	3157(76)
635(18)	1151(48)	3185(38)	604(0)	1150(0)	3163(84)
669(98)	1156(0)	3192(4)	650(18)	1169(60)	3169(15)
686(32)	1219(4)	3208(1)	665(67)	1219(3)	3183(3)
703(2)	1279(95)	3236(2)	699(6)	1285(1)	3210(14)
706(110)	1285(1)	3237(6)	707(47)	1296(1)	3212(16)
715(48)	1285(3)	3256(0)	721(71)	1298(102)	3234(4)

Table S44. Harmonic vibrational frequencies (in cm^{-1}) and infrared intensities (in parentheses, in km/mol) for the $C_6H_6FeC_6H_6$ structure **Fe1-66T**

B3PW91				M06-L		
31(0)	844(2)	1334(0)		36(0)	831(1)	1333(0)
94(0)	844(2)	1382(0)		59(0)	831(1)	1395(0)
94(0)	884(0)	1385(0)		60(0)	849(0)	1400(0)
118(0)	884(0)	1463(8)		108(0)	849(0)	1464(3)
118(0)	909(0)	1463(8)		108(0)	889(0)	1464(3)
205(0)	909(0)	1464(0)		183(0)	890(0)	1465(0)
233(1)	939(0)	1464(0)		184(0)	920(0)	1465(0)
233(1)	942(0)	1535(0)		190(0)	921(0)	1534(0)
250(82)	988(0)	1535(0)		195(72)	990(0)	1534(0)
380(0)	991(0)	1563(0)		372(0)	992(44)	1572(0)
380(0)	991(36)	1563(0)		373(0)	992(0)	1573(0)
415(0)	996(0)	3200(0)		414(0)	994(0)	3166(0)
415(0)	1025(0)	3201(0)		415(0)	1027(0)	3166(0)
599(0)	1025(0)	3205(0)		580(0)	1027(0)	3173(0)
602(0)	1027(19)	3205(0)		593(0)	1028(16)	3173(0)
602(0)	1027(19)	3207(0)		596(0)	1029(16)	3174(0)
607(0)	1148(0)	3207(0)		597(0)	1146(0)	3175(0)
607(0)	1148(0)	3219(0)		604(0)	1148(0)	3186(0)
623(0)	1151(0)	3219(0)		605(0)	1148(0)	3187(0)
723(124)	1154(0)	3221(38)		714(92)	1148(0)	3187(131)
738(0)	1159(0)	3221(38)		726(0)	1160(0)	3188(132)
829(0)	1159(0)	3229(4)		817(0)	1160(0)	3195(27)
829(0)	1334(0)	3230(0)		818(0)	1332(0)	3196(0)

Table S45. Harmonic vibrational frequencies (in cm^{-1}) and infrared intensities (in parentheses, in km/mol) for the $C_5H_5FeC_7H_7$ structure **Fe2-57S**

B3PW91			M06-L		
41(0)	842(1)	1329(1)	36(0)	831(8)	1325(0)
134(1)	843(1)	1386(13)	131(2)	838(0)	1385(7)
179(1)	867(17)	1404(0)	174(1)	862(13)	1402(1)
258(1)	897(8)	1415(0)	260(1)	875(1)	1413(0)
298(0)	901(1)	1419(0)	296(0)	887(1)	1420(0)
302(1)	906(1)	1452(1)	302(0)	896(6)	1445(0)
342(1)	912(1)	1456(2)	351(1)	901(1)	1452(2)
364(0)	941(7)	1464(2)	372(0)	925(0)	1461(0)
417(6)	947(18)	1499(0)	432(6)	938(0)	1500(3)
425(9)	951(0)	1508(5)	437(5)	942(23)	1500(0)
451(10)	984(1)	1723(16)	456(13)	973(1)	1718(20)
493(28)	996(2)	3133(21)	502(31)	985(2)	3093(43)
520(19)	1019(11)	3134(10)	535(17)	1017(7)	3093(21)
593(5)	1020(2)	3155(7)	586(3)	1018(3)	3117(14)
598(1)	1028(10)	3161(8)	593(1)	1028(7)	3119(55)
601(0)	1068(0)	3165(44)	599(0)	1065(0)	3126(66)
608(2)	1071(0)	3181(49)	610(3)	1071(0)	3153(115)
739(39)	1115(0)	3197(18)	734(32)	1112(0)	3157(41)
793(9)	1145(10)	3244(0)	788(6)	1142(15)	3209(0)
822(27)	1147(3)	3250(1)	807(9)	1144(5)	3215(8)
834(22)	1183(1)	3262(5)	824(10)	1178(1)	3231(23)
839(3)	1199(0)	3263(3)	824(4)	1191(0)	3231(28)
842(5)	1264(0)	3277(1)	830(5)	1255(0)	3245(9)

Table S46. Harmonic vibrational frequencies (in cm^{-1}) and infrared intensities (in parentheses, in km/mol) for the $C_6H_6FeC_6H_6$ structure **Fe3-66S**

B3PW91				M06-L		
11(0)	863(4)	1332(0)		-3(0)	850(4)	1326(0)
126(2)	882(14)	1359(9)		120(2)	865(9)	1354(5)
172(1)	906(0)	1383(9)		168(1)	885(5)	1380(5)
241(3)	907(2)	1443(0)		244(2)	894(1)	1431(0)
293(2)	915(4)	1446(2)		298(2)	895(0)	1448(1)
312(1)	916(1)	1453(4)		328(1)	897(5)	1455(2)
351(9)	927(2)	1463(2)		372(9)	908(0)	1462(2)
386(3)	953(2)	1471(2)		399(2)	927(1)	1475(0)
407(1)	957(2)	1540(0)		414(3)	950(2)	1537(0)
422(8)	979(20)	1557(0)		428(8)	979(19)	1556(0)
437(36)	1001(0)	1658(3)		447(29)	999(0)	1660(7)
463(1)	1002(8)	3161(24)		460(1)	1002(11)	3126(48)
511(38)	1009(0)	3164(1)		514(30)	1008(0)	3128(2)
581(3)	1018(7)	3184(9)		587(5)	1017(5)	3152(12)
615(1)	1039(6)	3193(19)		614(0)	1041(4)	3158(58)
620(0)	1054(8)	3205(37)		621(1)	1054(9)	3168(61)
629(0)	1113(0)	3207(0)		623(0)	1109(0)	3170(1)
633(2)	1116(2)	3210(20)		633(1)	1109(2)	3176(1)
665(5)	1126(1)	3212(1)		667(5)	1125(1)	3181(14)
722(51)	1152(0)	3217(4)		719(43)	1148(0)	3184(77)
796(28)	1157(0)	3226(11)		791(16)	1150(0)	3189(46)
842(2)	1158(0)	3227(14)		834(2)	1156(0)	3191(52)
848(2)	1301(0)	3237(5)		837(1)	1296(0)	3200(20)

Table S47. Harmonic vibrational frequencies (in cm^{-1}) and infrared intensities (in parentheses, in km/mol) for the $C_5H_5FeC_7H_7$ structure **Fe4-57T**

B3PW91			M06-L		
30(0)	834(1)	1363(0)	12(0)	825(1)	1364(0)
62(0)	835(0)	1391(3)	65(0)	829(14)	1401(1)
88(1)	865(0)	1400(0)	116(0)	866(0)	1405(0)
118(0)	879(1)	1414(4)	157(1)	867(0)	1430(0)
139(0)	885(1)	1426(6)	166(0)	869(0)	1438(1)
245(10)	889(0)	1452(2)	199(9)	874(1)	1456(0)
275(1)	892(10)	1466(0)	279(1)	875(2)	1457(0)
298(1)	902(1)	1472(1)	327(2)	891(4)	1461(0)
327(4)	929(4)	1509(10)	332(2)	908(2)	1512(4)
342(9)	934(3)	1526(2)	338(4)	928(0)	1551(0)
416(2)	959(1)	1631(2)	408(1)	930(0)	1558(1)
431(1)	964(0)	3160(2)	415(2)	946(7)	3136(4)
465(5)	996(3)	3167(1)	505(12)	1008(3)	3137(1)
530(21)	1017(16)	3173(6)	537(0)	1022(11)	3146(12)
573(2)	1026(17)	3178(2)	553(2)	1025(13)	3151(19)
593(1)	1065(0)	3186(33)	591(1)	1065(0)	3160(43)
607(2)	1068(1)	3193(22)	596(1)	1068(0)	3160(84)
719(82)	1143(4)	3202(19)	698(76)	1143(12)	3182(47)
778(0)	1159(3)	3240(0)	778(1)	1169(0)	3213(0)
785(58)	1182(1)	3242(0)	779(13)	1183(0)	3213(0)
797(42)	1240(1)	3256(5)	791(4)	1234(0)	3231(28)
805(9)	1250(1)	3257(4)	796(37)	1241(0)	3231(28)
813(2)	1265(0)	3269(3)	817(7)	1258(0)	3244(12)

Table S48. Harmonic vibrational frequencies (in cm^{-1}) and infrared intensities (in parentheses, in km/mol) for the $C_5H_5FeC_7H_7$ structure **Fe5-57P**

B3PW91			M06-L		
19(0)	795(5)	1334(8)	19(0)	778(77)	1335(4)
62(0)	847(0)	1374(33)	54(0)	841(0)	1377(21)
75(0)	847(0)	1399(0)	68(0)	842(0)	1404(2)
156(2)	870(4)	1402(0)	126(5)	854(0)	1405(3)
158(0)	874(0)	1406(7)	169(0)	863(0)	1409(0)
167(0)	881(0)	1452(17)	173(0)	874(5)	1447(14)
212(1)	897(2)	1463(1)	229(0)	881(3)	1465(0)
239(1)	900(16)	1464(0)	250(3)	898(9)	1466(0)
260(5)	923(7)	1490(11)	268(0)	913(6)	1485(7)
281(2)	946(0)	1544(22)	284(1)	926(2)	1526(24)
323(29)	969(5)	1717(24)	319(23)	944(8)	1696(20)
356(1)	978(7)	3153(7)	384(1)	956(7)	3127(14)
400(0)	993(2)	3153(5)	398(1)	989(4)	3128(17)
513(36)	1023(20)	3156(1)	496(25)	1025(17)	3131(0)
575(0)	1024(21)	3173(23)	569(0)	1026(17)	3150(42)
608(0)	1068(0)	3179(27)	601(0)	1069(0)	3158(68)
614(0)	1070(0)	3184(18)	605(0)	1072(0)	3162(42)
725(16)	1123(0)	3217(6)	714(10)	1122(0)	3190(22)
754(0)	1146(0)	3239(0)	736(1)	1149(1)	3214(0)
761(137)	1165(7)	3240(0)	744(97)	1165(7)	3216(0)
773(17)	1205(3)	3255(3)	753(12)	1203(3)	3232(22)
773(4)	1214(8)	3257(3)	755(3)	1214(6)	3236(21)
782(93)	1264(0)	3269(0)	768(13)	1259(0)	3249(4)

Table S49. Harmonic vibrational frequencies (in cm^{-1}) and infrared intensities (in parentheses, in km/mol) for the $C_6H_6FeC_6H_6$ structure **Fe6-66P**

B3PW91			M06-L		
44(0)	806(0)	1334(0)	42(0)	690(59)	1322(0)
68(0)	812(1)	1358(0)	49(0)	703(1)	1323(0)
74(0)	822(0)	1366(6)	63(0)	739(2)	1360(0)
93(0)	834(2)	1368(0)	113(0)	753(0)	1388(13)
136(0)	894(0)	1398(0)	165(131)	764(1)	1398(0)
167(0)	898(0)	1440(2173)	178(0)	853(366)	1400(1091)
174(2)	908(1812)	1453(3)	180(0)	861(1394)	1448(1)
177(0)	924(0)	1453(0)	196(0)	891(0)	1450(1)
214(0)	928(2)	1459(12)	205(6)	902(27)	1453(2)
303(0)	984(0)	1460(0)	212(0)	911(0)	1464(12)
313(0)	995(12)	1581(0)	313(0)	960(42)	1528(0)
316(0)	998(0)	3200(0)	370(0)	975(5)	3174(53)
368(230)	1001(40)	3200(0)	395(14)	995(25)	3175(22)
571(0)	1001(0)	3202(0)	422(0)	1009(0)	3193(8)
574(260)	1013(0)	3202(3)	568(0)	1012(0)	3193(37)
585(7)	1023(0)	3211(147)	572(1)	1024(9)	3198(0)
595(0)	1024(11)	3214(0)	589(29)	1028(0)	3204(0)
597(0)	1028(0)	3218(0)	600(33)	1035(4)	3212(25)
626(0)	1123(629)	3219(37)	608(0)	1102(271)	3216(36)
656(817)	1153(0)	3237(11)	628(0)	1140(0)	3220(20)
677(0)	1153(0)	3237(0)	653(212)	1142(0)	3222(25)
731(6)	1169(0)	3239(52)	660(27)	1148(1)	3227(1)
732(0)	1334(0)	3242(0)	673(1)	1276(0)	3229(2)

Table S50. Harmonic vibrational frequencies (in cm^{-1}) and infrared intensities (in parentheses, in km/mol) for the $C_4H_4FeC_8H_8$ structure **Fe7-48S**

B3PW91			M06-L		
65(0)	819(21)	1280(21)	73(0)	802(29)	1284(27)
137(0)	825(9)	1338(1)	134(0)	817(6)	1333(0)
177(0)	849(9)	1364(0)	173(0)	844(8)	1369(0)
212(0)	885(14)	1372(1)	206(0)	884(10)	1379(0)
226(2)	906(5)	1398(12)	223(1)	898(5)	1396(7)
293(4)	927(2)	1402(0)	295(3)	917(9)	1399(2)
309(1)	930(13)	1471(2)	330(1)	924(1)	1467(4)
335(7)	942(4)	1507(0)	340(7)	930(6)	1501(1)
382(3)	952(11)	1524(7)	393(4)	951(0)	1528(4)
397(1)	954(11)	1534(2)	409(3)	955(8)	1530(1)
450(19)	960(0)	1749(23)	465(7)	956(1)	1744(30)
460(20)	968(0)	3124(10)	478(12)	959(2)	3084(21)
468(4)	972(2)	3125(13)	478(15)	965(10)	3084(29)
523(5)	972(3)	3146(3)	521(6)	972(1)	3107(48)
535(37)	991(3)	3152(26)	546(34)	986(2)	3111(4)
615(1)	1014(1)	3154(2)	610(0)	1001(2)	3115(41)
638(9)	1097(0)	3167(27)	635(8)	1094(0)	3126(57)
648(1)	1151(5)	3173(53)	650(2)	1147(6)	3138(88)
721(5)	1171(0)	3180(25)	701(6)	1161(0)	3144(96)
736(2)	1189(0)	3238(0)	731(0)	1183(1)	3190(1)
767(11)	1236(0)	3253(8)	757(14)	1228(0)	3207(38)
790(31)	1240(0)	3263(4)	768(9)	1259(0)	3223(28)
803(31)	1272(0)	3280(0)	801(16)	1265(0)	3240(6)

Table S51. Harmonic vibrational frequencies (in cm^{-1}) and infrared intensities (in parentheses, in km/mol) for the $C_4H_4FeC_8H_8$ structure **Fe8-48T**

B3PW91			M06-L		
83(0)	781(0)	1309(4)	68(0)	769(2)	1309(0)
98(1)	788(39)	1314(0)	105(1)	779(35)	1315(17)
124(0)	790(4)	1314(19)	118(0)	785(5)	1319(3)
202(0)	804(49)	1361(1)	207(0)	800(43)	1357(0)
218(0)	839(3)	1367(18)	215(0)	827(3)	1365(9)
227(0)	890(51)	1403(0)	231(0)	889(36)	1408(0)
268(2)	898(4)	1410(0)	273(1)	901(2)	1409(0)
298(5)	908(33)	1453(11)	316(2)	913(16)	1431(9)
332(1)	926(0)	1462(31)	346(0)	920(12)	1442(20)
396(1)	942(0)	1687(6)	407(0)	929(0)	1687(10)
401(2)	946(6)	1714(19)	414(1)	932(9)	1710(26)
409(0)	955(0)	3142(2)	416(0)	938(0)	3112(4)
442(1)	959(7)	3144(7)	445(0)	964(6)	3114(12)
450(24)	973(14)	3152(9)	458(13)	972(0)	3121(19)
460(16)	982(0)	3154(0)	482(7)	980(10)	3123(1)
587(15)	986(3)	3172(33)	583(13)	986(1)	3137(45)
629(0)	1018(0)	3176(42)	629(0)	1012(0)	3140(82)
635(0)	1149(29)	3176(24)	636(0)	1146(25)	3146(90)
650(0)	1163(1)	3183(18)	645(1)	1155(1)	3152(72)
689(4)	1166(0)	3245(0)	674(1)	1159(1)	3209(0)
705(0)	1167(1)	3263(1)	689(2)	1160(0)	3229(16)
709(1)	1172(5)	3267(2)	710(0)	1166(5)	3234(21)
758(59)	1185(0)	3286(0)	754(48)	1198(1)	3254(4)

Table S52. Harmonic vibrational frequencies (in cm^{-1}) and infrared intensities (in parentheses, in km/mol) for the $C_4H_4FeC_8H_8$ structure **Fe9-48P**

B3PW91				M06-L		
42(2)	758(1)	1275(67)		57(0)	737(12)	1276(65)
53(0)	765(45)	1314(1)		60(0)	758(36)	1330(0)
85(0)	798(16)	1349(1)		75(0)	796(11)	1346(1)
109(6)	814(10)	1378(20)		112(3)	819(12)	1377(2)
131(3)	850(19)	1380(1)		130(3)	844(11)	1384(11)
180(1)	865(31)	1411(8)		174(1)	858(39)	1409(4)
225(1)	896(23)	1440(15)		223(1)	903(15)	1443(6)
242(3)	904(25)	1480(62)		240(1)	919(1)	1483(54)
304(1)	917(23)	1519(4)		326(1)	922(23)	1525(2)
330(6)	934(22)	1532(7)		331(3)	927(3)	1534(12)
337(2)	939(1)	1682(16)		343(2)	936(11)	1698(29)
353(3)	956(3)	3128(3)		363(1)	947(12)	3107(5)
375(6)	960(27)	3147(9)		386(5)	952(9)	3119(10)
435(2)	964(0)	3150(4)		441(4)	957(20)	3121(10)
463(3)	967(3)	3155(13)		464(3)	964(2)	3128(40)
555(3)	992(3)	3168(13)		544(0)	985(4)	3143(17)
572(1)	1079(2)	3177(28)		565(1)	1079(1)	3147(76)
625(9)	1148(44)	3191(29)		610(4)	1156(0)	3159(42)
628(2)	1164(0)	3193(9)		614(1)	1165(9)	3170(53)
664(3)	1167(10)	3245(0)		624(7)	1179(43)	3210(0)
700(33)	1201(1)	3261(1)		694(33)	1195(0)	3228(15)
712(44)	1237(2)	3267(0)		707(23)	1238(0)	3235(12)
726(32)	1267(1)	3285(1)		718(36)	1265(2)	3256(2)

Table S53. Harmonic vibrational frequencies (in cm^{-1}) and infrared intensities (in parentheses, in km/mol) for the $C_6H_6CoC_6H_6$ structure **Co1-66D**

B3PW91			M06-L		
-1(0)	856(0)	1336(0)	10(0)	839(0)	1334(0)
44(0)	857(1)	1392(8)	72(0)	848(2)	1403(5)
93(0)	883(8)	1407(0)	101(0)	862(11)	1425(1)
119(0)	905(0)	1448(3)	143(0)	885(0)	1455(0)
168(0)	913(2)	1459(3)	178(0)	894(3)	1455(1)
260(0)	938(35)	1464(4)	266(1)	932(0)	1463(1)
264(1)	952(0)	1473(2)	291(2)	937(18)	1478(0)
265(1)	957(0)	1539(0)	292(1)	950(13)	1541(1)
358(20)	969(0)	1545(2)	357(13)	956(0)	1549(2)
391(0)	990(4)	1568(38)	404(0)	998(3)	1569(18)
394(3)	996(6)	1635(2)	405(2)	999(9)	1633(1)
436(0)	999(0)	3151(1)	435(0)	1001(0)	3119(3)
447(18)	1018(5)	3164(11)	439(8)	1020(2)	3135(30)
565(1)	1022(13)	3183(1)	567(2)	1021(13)	3156(11)
597(4)	1030(11)	3190(6)	600(3)	1033(9)	3163(15)
608(0)	1032(2)	3203(33)	609(0)	1036(1)	3175(0)
614(2)	1137(10)	3207(0)	611(2)	1134(7)	3178(64)
629(0)	1151(0)	3213(0)	619(1)	1145(0)	3181(3)
677(0)	1152(0)	3214(0)	673(0)	1149(0)	3182(7)
700(78)	1156(0)	3215(20)	694(59)	1155(0)	3192(38)
755(17)	1170(2)	3225(11)	751(15)	1166(1)	3193(46)
810(0)	1180(0)	3226(12)	800(1)	1179(0)	3196(48)
824(105)	1334(0)	3234(3)	817(82)	1328(0)	3203(17)

Table S54. Harmonic vibrational frequencies (in cm^{-1}) and infrared intensities (in parentheses, in km/mol) for the $C_5H_5CoC_7H_7$ structure **Co2-57D**

B3PW91			M06-L		
30(0)	836(0)	1363(1)	18(0)	825(0)	1362(0)
57(1)	838(2)	1393(5)	40(1)	829(0)	1400(3)
77(0)	864(13)	1401(4)	76(0)	863(10)	1401(1)
125(0)	883(2)	1423(3)	120(0)	869(1)	1427(1)
166(1)	886(1)	1443(6)	172(1)	873(6)	1448(2)
262(7)	887(2)	1453(2)	272(4)	878(0)	1453(0)
305(1)	894(0)	1462(0)	331(1)	889(2)	1458(0)
325(1)	905(3)	1468(0)	350(2)	899(3)	1476(0)
349(1)	929(3)	1489(2)	351(1)	933(3)	1498(3)
351(1)	940(3)	1589(2)	361(1)	941(2)	1588(3)
435(6)	973(0)	1661(0)	430(5)	958(1)	1654(0)
438(2)	981(0)	3162(0)	442(2)	966(0)	3133(7)
472(0)	991(3)	3163(4)	475(1)	992(3)	3134(3)
573(22)	1019(15)	3174(4)	583(15)	1021(12)	3146(23)
590(3)	1024(20)	3176(2)	587(2)	1022(15)	3149(0)
601(3)	1066(0)	3188(40)	601(0)	1067(0)	3162(55)
606(0)	1067(1)	3194(27)	601(7)	1067(0)	3164(79)
720(87)	1142(4)	3200(16)	712(68)	1142(10)	3178(54)
784(16)	1170(3)	3245(0)	777(3)	1171(3)	3217(0)
798(0)	1182(1)	3245(0)	787(0)	1179(1)	3218(1)
800(71)	1255(1)	3259(4)	798(50)	1251(1)	3233(25)
814(2)	1263(0)	3262(3)	800(2)	1257(0)	3238(24)
821(2)	1264(0)	3273(2)	821(3)	1258(1)	3248(11)

Table S55. Harmonic vibrational frequencies (in cm^{-1}) and infrared intensities (in parentheses, in km/mol) for the $C_6H_6CoC_6H_6$ structure **Co3-66Q**

B3PW91			M06-L		
25(0)	766(3)	1331(0)	25(0)	766(3)	1331(0)
49(0)	784(0)	1331(0)	49(0)	784(0)	1331(0)
97(0)	785(3)	1370(7)	97(0)	785(3)	1370(7)
107(0)	789(4)	1381(21)	107(0)	789(4)	1381(21)
172(0)	796(0)	1391(0)	172(0)	796(0)	1391(0)
181(0)	851(3088)	1426(2137)	181(0)	851(3088)	1426(2137)
183(0)	931(11)	1455(1)	183(0)	931(11)	1455(1)
192(0)	937(0)	1460(0)	192(0)	937(0)	1460(0)
206(174)	939(0)	1462(12)	206(174)	939(0)	1462(12)
207(0)	942(1)	1464(0)	207(0)	942(1)	1464(0)
328(0)	984(0)	1548(0)	328(0)	984(0)	1548(0)
364(1)	986(61)	3202(141)	364(1)	986(61)	3202(141)
408(6)	988(0)	3203(0)	408(6)	988(0)	3203(0)
421(0)	1001(0)	3205(12)	421(0)	1001(0)	3205(12)
573(2)	1002(0)	3206(0)	573(2)	1002(0)	3206(0)
603(84)	1032(0)	3215(0)	603(84)	1032(0)	3215(0)
605(1)	1035(0)	3220(2)	605(1)	1035(0)	3220(2)
609(0)	1036(10)	3231(0)	609(0)	1036(10)	3231(0)
641(0)	1117(456)	3232(24)	641(0)	1117(456)	3232(24)
674(0)	1152(0)	3238(0)	674(0)	1152(0)	3238(0)
694(293)	1154(0)	3239(10)	694(293)	1154(0)	3239(10)
708(0)	1154(1)	3243(46)	708(0)	1154(1)	3243(46)
756(0)	1285(0)	3246(0)	756(0)	1285(0)	3246(0)

Table S56. Harmonic vibrational frequencies (in cm^{-1}) and infrared intensities (in parentheses, in km/mol) for the $C_5H_5CoC_7H_7$ structure **Co4-57Q**

B3PW91			M06-L		
7(0)	783(7)	1278(0)	-29(5)	771(78)	1271(0)
42(0)	820(0)	1376(0)	9(0)	795(1)	1373(0)
104(0)	844(0)	1403(0)	105(0)	836(0)	1405(0)
111(0)	844(0)	1403(0)	114(0)	836(0)	1406(0)
136(0)	860(3)	1434(22)	134(0)	854(0)	1442(4)
147(0)	872(0)	1451(1)	135(0)	855(0)	1450(0)
166(0)	873(0)	1462(0)	183(0)	862(4)	1463(0)
201(6)	894(1)	1462(0)	204(4)	895(1)	1463(0)
240(1)	908(2)	1518(48)	248(0)	913(1)	1515(7)
246(0)	947(1)	1519(8)	253(0)	932(1)	1562(45)
299(0)	949(1)	1592(6)	304(0)	935(1)	1589(2)
317(0)	957(1)	3183(2)	307(0)	945(0)	3150(8)
407(0)	964(5)	3183(2)	407(0)	966(6)	3151(14)
445(0)	975(13)	3189(2)	446(0)	977(18)	3158(1)
483(12)	1022(19)	3203(8)	485(13)	1024(16)	3174(24)
613(0)	1023(19)	3216(13)	606(0)	1024(16)	3188(51)
613(0)	1066(0)	3217(6)	607(0)	1067(0)	3189(32)
640(8)	1067(0)	3224(2)	656(8)	1068(0)	3196(7)
710(116)	1147(1)	3237(0)	699(68)	1148(2)	3210(0)
755(13)	1172(3)	3237(0)	728(27)	1169(3)	3210(0)
760(0)	1215(3)	3253(8)	742(0)	1212(4)	3229(32)
772(10)	1259(5)	3253(7)	751(5)	1255(2)	3230(32)
778(99)	1263(0)	3267(1)	762(7)	1257(0)	3244(6)

Table S57. Harmonic vibrational frequencies (in cm^{-1}) and infrared intensities (in parentheses, in km/mol) for the $C_5H_5CoC_7H_7$ structure **Co5-57X**

B3PW91			M06-L		
6(0)	794(0)	1365(0)	2(0)	778(3)	1366(0)
80(1)	794(0)	1386(0)	54(1)	784(94)	1395(6)
81(1)	846(0)	1386(0)	88(1)	830(0)	1400(0)
129(0)	846(0)	1437(0)	100(0)	831(0)	1440(0)
129(0)	854(0)	1438(0)	112(0)	832(0)	1442(0)
187(0)	854(0)	1461(1)	178(2)	838(1)	1457(1)
192(0)	854(0)	1461(1)	184(0)	856(0)	1467(0)
192(0)	881(0)	1488(0)	202(1)	866(0)	1492(1)
232(0)	881(0)	1488(0)	206(0)	871(0)	1499(0)
232(0)	896(0)	1567(0)	251(0)	896(0)	1586(0)
331(2)	896(0)	1567(0)	323(11)	896(0)	1597(2)
397(0)	947(19)	3188(0)	396(0)	947(13)	3154(1)
397(0)	947(19)	3188(0)	398(0)	951(13)	3155(0)
486(0)	1024(18)	3195(0)	457(0)	1021(14)	3162(0)
487(0)	1024(19)	3195(0)	480(0)	1029(15)	3164(0)
606(0)	1073(0)	3209(21)	599(0)	1067(2)	3177(77)
606(0)	1073(0)	3210(21)	606(0)	1067(0)	3180(77)
675(144)	1146(0)	3219(2)	656(119)	1146(1)	3189(6)
715(5)	1151(0)	3253(0)	697(4)	1152(0)	3220(1)
717(5)	1151(0)	3253(0)	706(3)	1156(0)	3223(0)
787(5)	1251(0)	3267(0)	758(2)	1248(0)	3238(13)
788(3)	1251(0)	3267(0)	767(0)	1248(0)	3244(14)
789(112)	1266(0)	3279(0)	772(0)	1261(0)	3254(3)

Table S58. Harmonic vibrational frequencies (in cm^{-1}) and infrared intensities (in parentheses, in km/mol) for the $C_6H_6CoC_6H_6$ structure **Co6-66X**

B3PW91			M06-L		
37(0)	767(0)	1320(0)	60(0)	741(0)	1318(0)
73(0)	767(0)	1354(29)	69(0)	744(0)	1340(0)
73(0)	781(0)	1354(29)	69(0)	754(0)	1349(0)
152(0)	784(9)	1362(0)	158(0)	754(0)	1358(28)
152(0)	921(0)	1369(0)	163(0)	898(0)	1358(28)
157(0)	921(0)	1414(2)	163(0)	899(0)	1414(0)
206(0)	923(0)	1414(2)	213(0)	899(0)	1414(0)
206(0)	925(45)	1517(1)	213(0)	905(0)	1520(5)
251(0)	925(45)	1517(1)	248(0)	924(41)	1520(5)
352(84)	927(0)	1621(1)	318(62)	924(41)	1618(2)
354(0)	974(0)	1622(0)	337(0)	972(0)	1619(0)
397(0)	974(1)	3196(0)	393(0)	972(0)	3169(0)
416(0)	998(0)	3197(0)	422(0)	1002(0)	3170(0)
516(14)	998(0)	3198(0)	514(12)	1002(0)	3171(3)
516(14)	1039(0)	3198(0)	514(12)	1022(0)	3171(3)
542(0)	1039(0)	3211(0)	534(0)	1039(0)	3186(0)
544(0)	1052(0)	3211(4)	535(1)	1039(0)	3186(11)
603(0)	1061(0)	3219(21)	600(0)	1052(0)	3193(60)
606(0)	1141(0)	3219(21)	604(0)	1136(0)	3193(60)
719(226)	1141(0)	3226(11)	708(168)	1136(0)	3203(44)
727(0)	1162(0)	3226(11)	713(4)	1162(0)	3203(44)
744(3)	1163(2)	3232(4)	713(4)	1163(1)	3209(17)
744(3)	1320(0)	3233(0)	715(0)	1318(0)	3210(0)

Table S59. Harmonic vibrational frequencies (in cm^{-1}) and infrared intensities (in parentheses, in km/mol) for the $C_4H_4CoC_8H_8$ structure **Co7-48D**

B3PW91			M06-L		
55(0)	791(8)	1272(19)	69(0)	785(4)	1274(21)
62(2)	793(4)	1323(0)	86(2)	787(1)	1317(0)
156(0)	799(50)	1358(4)	157(0)	797(21)	1365(4)
195(2)	818(49)	1362(0)	207(0)	800(66)	1369(1)
205(2)	849(3)	1374(19)	207(0)	839(5)	1372(9)
215(0)	910(42)	1379(1)	243(0)	906(34)	1374(1)
266(12)	914(13)	1426(0)	283(10)	915(12)	1423(0)
284(4)	930(0)	1506(16)	289(2)	926(3)	1481(14)
378(14)	935(4)	1517(23)	392(8)	931(0)	1492(17)
384(0)	942(8)	1681(7)	401(0)	935(8)	1686(11)
407(9)	945(0)	1714(9)	410(5)	943(2)	1710(13)
409(4)	954(14)	3143(0)	429(0)	948(2)	3104(1)
416(35)	955(24)	3144(4)	438(31)	955(21)	3105(14)
433(5)	960(1)	3151(0)	448(7)	964(7)	3115(33)
476(6)	979(0)	3154(1)	489(4)	968(0)	3120(24)
589(17)	997(5)	3160(14)	592(7)	999(4)	3124(14)
607(1)	1024(0)	3162(41)	613(3)	1018(0)	3128(78)
628(8)	1155(18)	3176(41)	620(9)	1151(18)	3147(92)
639(0)	1169(0)	3179(36)	642(0)	1161(0)	3152(89)
694(6)	1170(6)	3234(3)	689(2)	1162(4)	3190(2)
712(7)	1173(0)	3248(7)	707(19)	1169(3)	3206(32)
730(45)	1180(2)	3262(1)	715(13)	1175(0)	3224(16)
770(8)	1187(0)	3282(0)	754(10)	1198(0)	3244(4)

Table S60. Harmonic vibrational frequencies (in cm^{-1}) and infrared intensities (in parentheses, in km/mol) for the $C_4H_4CoC_8H_8$ structure **Co8-48Q**

B3PW91			M06-L		
59(1)	771(3)	1297(1)	35(1)	737(6)	1306(1)
64(0)	779(2)	1317(128)	61(0)	780(0)	1313(63)
90(0)	785(6)	1386(1)	87(0)	786(4)	1392(0)
113(0)	819(9)	1425(12)	106(1)	807(8)	1426(9)
134(0)	852(14)	1426(5)	130(0)	836(8)	1441(1)
177(8)	871(20)	1440(2)	183(4)	868(5)	1451(0)
205(1)	893(0)	1487(1)	229(1)	886(1)	1500(0)
256(2)	900(5)	1505(5)	270(1)	898(5)	1519(3)
273(0)	907(0)	1550(11)	283(0)	905(8)	1531(7)
284(0)	925(1)	1580(3)	290(1)	916(0)	1557(1)
313(1)	933(1)	1631(17)	318(0)	918(5)	1589(3)
387(1)	933(63)	3150(0)	384(1)	941(16)	3128(1)
392(7)	953(6)	3153(1)	419(8)	942(1)	3131(2)
449(4)	956(0)	3161(0)	456(0)	947(8)	3139(1)
458(2)	959(2)	3170(6)	456(7)	958(0)	3147(1)
503(0)	985(17)	3171(2)	502(2)	985(12)	3147(3)
609(3)	1118(1)	3180(35)	591(26)	1146(1)	3157(101)
610(10)	1156(41)	3185(40)	596(3)	1157(0)	3163(85)
667(72)	1166(0)	3192(8)	668(64)	1165(9)	3168(29)
702(7)	1212(5)	3238(0)	677(1)	1211(3)	3194(0)
714(1)	1280(2)	3256(1)	710(5)	1290(1)	3215(18)
716(42)	1289(2)	3263(2)	720(25)	1301(2)	3223(25)
738(54)	1294(4)	3280(0)	725(27)	1306(5)	3242(5)

Table S61. Harmonic vibrational frequencies (in cm^{-1}) and infrared intensities (in parentheses, in km/mol) for the $C_4H_4CoC_8H_8$ structure **Co9-48X**

B3PW91				M06-L		
23(0)	747(0)	1329(0)		-22(0)	749(2)	1330(0)
50(0)	763(5)	1330(1)		44(0)	749(3)	1341(0)
50(0)	763(5)	1330(1)		44(0)	749(3)	1341(0)
87(0)	764(7)	1356(7)		112(1)	773(4)	1400(0)
116(1)	837(0)	1399(0)		112(1)	835(0)	1455(0)
116(1)	875(0)	1450(1)		128(0)	846(0)	1455(0)
153(0)	911(0)	1450(1)		144(0)	899(0)	1482(2)
153(0)	912(25)	1517(1)		176(0)	899(0)	1522(0)
203(0)	912(25)	1517(1)		219(0)	920(0)	1522(0)
203(0)	917(0)	1521(0)		219(0)	922(0)	1535(0)
304(0)	917(0)	1557(0)		304(0)	922(0)	1543(0)
305(20)	919(13)	3167(0)		305(0)	924(35)	3147(0)
308(0)	919(13)	3174(0)		324(10)	924(35)	3152(0)
468(0)	944(0)	3174(0)		464(0)	939(0)	3152(0)
468(0)	949(0)	3185(0)		464(0)	952(0)	3163(0)
506(0)	956(0)	3186(0)		474(0)	970(0)	3163(0)
546(0)	1170(0)	3199(28)		474(0)	1159(0)	3177(79)
546(0)	1184(0)	3199(28)		537(0)	1199(0)	3177(79)
558(0)	1213(0)	3206(3)		552(0)	1206(0)	3184(7)
664(140)	1221(0)	3249(0)		653(117)	1252(0)	3206(0)
691(0)	1271(0)	3264(0)		670(0)	1275(2)	3224(13)
693(44)	1319(3)	3264(0)		682(47)	1324(0)	3224(13)
747(0)	1329(0)	3288(0)		749(2)	1330(0)	3251(3)

Table S62. Harmonic vibrational frequencies (in cm^{-1}) and infrared intensities (in parentheses, in km/mol) for the $C_6H_6NiC_6H_6$ structure **Ni1-66S**

B3PW91				M06-L		
0(0)	853(5)	1339(0)		-6(0)	841(7)	1336(0)
64(0)	866(0)	1388(0)		61(0)	853(0)	1404(0)
95(0)	912(4)	1396(2)		91(0)	887(4)	1407(1)
134(0)	913(1)	1455(4)		135(0)	891(1)	1458(1)
155(0)	923(2)	1466(6)		158(0)	900(2)	1464(2)
180(1)	946(21)	1471(5)		202(2)	935(10)	1468(2)
209(0)	960(1)	1475(7)		230(0)	940(1)	1478(2)
244(2)	963(3)	1559(1)		250(1)	959(0)	1556(1)
339(31)	972(0)	1566(0)		336(22)	961(13)	1561(0)
373(3)	991(4)	1576(14)		382(3)	996(0)	1573(8)
389(0)	993(0)	1636(1)		396(0)	999(3)	1630(1)
436(16)	1000(2)	3158(2)		428(9)	1002(4)	3132(3)
456(0)	1027(8)	3170(12)		459(0)	1028(7)	3144(25)
583(0)	1027(6)	3182(3)		584(0)	1029(6)	3157(15)
599(5)	1033(9)	3190(10)		599(3)	1034(8)	3164(24)
604(2)	1036(3)	3202(34)		603(3)	1037(2)	3173(0)
607(0)	1144(4)	3204(0)		610(0)	1139(2)	3177(70)
623(0)	1155(0)	3210(0)		614(0)	1149(0)	3181(0)
690(0)	1157(0)	3212(0)		684(0)	1155(0)	3181(0)
705(101)	1161(0)	3214(19)		697(74)	1158(0)	3192(40)
737(30)	1170(1)	3223(14)		729(20)	1166(0)	3193(61)
826(0)	1181(1)	3224(14)		816(0)	1180(0)	3193(55)
836(66)	1339(0)	3232(5)		824(58)	1334(0)	3202(18)

Table S63. Harmonic vibrational frequencies (in cm^{-1}) and infrared intensities (in parentheses, in km/mol) for the $C_5H_5NiC_7H_7$ structure **Ni2-57S**

B3PW91			M06-L		
11(0)	831(6)	1368(0)	-5(0)	824(6)	1367(0)
76(0)	838(0)	1392(1)	73(0)	831(0)	1397(0)
102(0)	867(20)	1397(6)	100(0)	856(5)	1398(3)
155(0)	879(8)	1431(5)	148(0)	863(0)	1434(1)
181(3)	882(0)	1449(2)	182(3)	868(6)	1449(0)
267(5)	885(2)	1453(6)	272(5)	880(19)	1459(2)
278(3)	887(0)	1468(0)	304(1)	893(2)	1464(0)
306(1)	925(5)	1490(0)	329(0)	916(3)	1493(0)
339(1)	937(1)	1502(0)	344(2)	929(0)	1513(0)
343(1)	938(1)	1574(1)	348(1)	941(3)	1570(1)
428(1)	972(0)	1656(0)	428(4)	958(1)	1641(0)
432(5)	983(0)	3156(0)	429(1)	969(0)	3126(13)
479(0)	1007(2)	3156(7)	468(0)	1007(2)	3127(7)
586(3)	1012(16)	3169(9)	585(2)	1015(14)	3143(30)
587(2)	1028(18)	3172(1)	591(1)	1028(14)	3146(1)
607(0)	1064(0)	3186(36)	605(0)	1065(0)	3162(69)
609(18)	1066(1)	3192(21)	618(13)	1065(0)	3164(45)
716(80)	1143(3)	3199(20)	706(66)	1144(7)	3178(54)
773(12)	1178(0)	3244(0)	762(3)	1172(0)	3218(0)
789(71)	1183(2)	3247(1)	785(54)	1187(2)	3220(2)
806(0)	1257(1)	3260(4)	789(1)	1253(1)	3235(26)
814(2)	1263(0)	3263(4)	805(2)	1256(0)	3240(25)
826(3)	1264(0)	3274(3)	821(0)	1256(0)	3251(13)

Table S64. Harmonic vibrational frequencies (in cm^{-1}) and infrared intensities (in parentheses, in km/mol) for the $C_6H_6NiC_6H_6$ structure **Ni3-66T**

B3PW91			M06-L		
-27(0)	798(13)	1330(0)	46(0)	778(3)	1324(0)
43(0)	806(0)	1380(37)	59(0)	786(0)	1388(28)
66(0)	855(0)	1386(0)	66(0)	829(0)	1396(0)
93(0)	858(9)	1411(0)	93(0)	835(4)	1415(0)
114(1)	883(0)	1435(0)	130(0)	869(0)	1430(0)
118(0)	919(7)	1436(6)	151(1)	896(5)	1431(2)
145(38)	934(589)	1470(0)	166(8)	914(0)	1465(0)
223(70)	935(0)	1478(75)	208(42)	914(0)	1478(46)
244(0)	935(0)	1482(0)	241(0)	941(353)	1482(0)
396(0)	970(0)	1572(587)	396(0)	965(0)	1573(264)
421(14)	978(39)	1620(0)	404(7)	971(37)	1617(0)
454(2)	986(0)	3195(0)	448(2)	986(0)	3170(0)
456(0)	993(0)	3197(0)	451(0)	996(0)	3171(0)
571(0)	998(1)	3203(0)	568(0)	1002(0)	3174(0)
574(1)	1045(0)	3203(1)	569(1)	1046(0)	3175(0)
577(0)	1045(8)	3207(50)	574(0)	1047(6)	3179(31)
603(1)	1108(0)	3208(0)	600(1)	1108(0)	3180(0)
622(0)	1134(0)	3219(0)	620(0)	1131(0)	3193(0)
646(0)	1143(43)	3220(37)	638(0)	1136(14)	3193(111)
681(38)	1147(0)	3221(0)	677(35)	1138(0)	3193(0)
705(0)	1155(37)	3222(31)	695(0)	1151(21)	3194(103)
768(0)	1163(0)	3230(1)	750(0)	1159(0)	3204(38)
790(0)	1330(0)	3230(0)	772(0)	1324(0)	3204(0)

Table S65. Harmonic vibrational frequencies (in cm^{-1}) and infrared intensities (in parentheses, in km/mol) for the $C_5H_5NiC_7H_7$ structure **Ni4-57T**

B3PW91			M06-L		
14(0)	801(0)	1341(7)	15(0)	783(3)	1342(6)
91(0)	844(0)	1383(38)	87(0)	835(0)	1383(31)
117(0)	845(0)	1397(0)	96(0)	837(0)	1402(0)
128(0)	872(1)	1398(0)	116(0)	858(0)	1402(0)
148(2)	879(0)	1422(4)	130(2)	858(0)	1425(1)
197(1)	882(0)	1452(18)	194(1)	877(1)	1448(18)
219(1)	892(3)	1461(1)	221(1)	878(3)	1459(0)
227(1)	912(12)	1466(1)	222(1)	907(9)	1469(0)
241(4)	928(9)	1502(0)	234(3)	922(8)	1500(0)
305(0)	947(1)	1568(15)	302(0)	929(2)	1559(19)
323(11)	982(0)	1722(14)	303(6)	961(0)	1710(15)
382(0)	983(1)	3151(9)	385(0)	963(2)	3116(20)
393(1)	990(5)	3152(7)	396(0)	988(6)	3118(18)
490(22)	1022(19)	3156(0)	472(21)	1021(16)	3129(0)
575(0)	1026(18)	3173(22)	569(0)	1029(15)	3144(22)
604(0)	1067(0)	3178(33)	595(0)	1068(0)	3148(87)
606(0)	1067(0)	3184(22)	601(0)	1068(0)	3158(67)
726(21)	1130(3)	3220(6)	716(12)	1130(3)	3195(21)
760(1)	1148(1)	3240(0)	742(68)	1148(2)	3213(0)
760(109)	1168(5)	3244(1)	743(0)	1168(4)	3217(3)
776(9)	1213(2)	3258(4)	753(16)	1212(3)	3233(25)
783(83)	1218(7)	3259(4)	766(4)	1215(6)	3237(25)
785(5)	1266(0)	3272(1)	772(69)	1260(0)	3249(8)

Table S66. Harmonic vibrational frequencies (in cm^{-1}) and infrared intensities (in parentheses, in km/mol) for the $C_5H_5NiC_7H_7$ structure **Ni5-57P**

B3PW91			M06-L		
3(0)	802(0)	1370(0)	2(0)	776(3)	1366(0)
104(0)	802(0)	1389(0)	101(0)	782(77)	1392(0)
104(0)	846(0)	1389(0)	102(0)	826(0)	1393(0)
116(0)	846(0)	1443(1)	126(0)	826(0)	1443(0)
117(0)	853(0)	1443(1)	127(0)	838(0)	1443(0)
179(0)	853(0)	1463(2)	193(0)	838(0)	1461(1)
180(0)	857(0)	1463(2)	203(0)	858(0)	1461(0)
192(1)	890(0)	1493(0)	204(0)	866(0)	1495(0)
249(0)	890(0)	1493(0)	261(0)	866(0)	1496(0)
249(0)	894(0)	1598(0)	261(0)	896(0)	1602(0)
341(4)	894(0)	1598(0)	328(1)	896(0)	1603(0)
399(0)	951(15)	3185(0)	397(0)	951(15)	3153(0)
399(0)	951(15)	3185(0)	398(0)	951(15)	3154(0)
488(0)	1026(16)	3191(0)	475(0)	1026(13)	3162(0)
489(0)	1026(17)	3192(0)	475(0)	1026(13)	3162(0)
604(0)	1067(0)	3206(26)	596(0)	1068(0)	3177(80)
605(0)	1067(0)	3206(26)	598(0)	1068(0)	3177(80)
677(133)	1148(0)	3216(2)	667(97)	1148(1)	3187(6)
726(4)	1152(0)	3253(0)	700(5)	1154(0)	3226(0)
728(4)	1152(0)	3253(0)	701(5)	1154(0)	3226(0)
794(113)	1256(0)	3267(1)	764(0)	1249(0)	3244(13)
797(4)	1256(0)	3267(1)	766(0)	1249(0)	3244(13)
797(4)	1268(0)	3280(0)	775(3)	1260(0)	3257(3)

Table S67. Harmonic vibrational frequencies (in cm^{-1}) and infrared intensities (in parentheses, in km/mol) for the $C_6H_6NiC_6H_6$ structure **Ni6-66P**

B3PW91			M06-L		
27(0)	666(152)	1320(0)	39(0)	601(20)	1308(0)
87(0)	689(44)	1324(0)	63(10)	685(105)	1321(0)
109(3)	691(0)	1327(0)	95(0)	688(0)	1323(0)
109(0)	742(28)	1372(43)	95(0)	710(0)	1384(39)
132(0)	772(1)	1386(14)	192(0)	720(7)	1389(0)
146(0)	912(0)	1417(1)	193(0)	881(0)	1438(0)
152(0)	918(48)	1454(7)	193(0)	900(0)	1440(15)
189(0)	921(47)	1484(38)	207(0)	903(0)	1453(0)
230(0)	925(0)	1507(0)	209(0)	904(1)	1487(37)
250(2)	948(0)	1518(2)	212(0)	904(0)	1489(5)
328(2)	952(0)	1608(8)	279(1)	927(44)	1541(1)
379(0)	959(3)	3197(0)	305(1)	927(40)	3175(0)
390(41)	969(0)	3200(8)	400(0)	940(97)	3176(0)
498(0)	975(0)	3201(0)	405(19)	964(0)	3176(27)
520(14)	999(0)	3202(0)	509(1)	1001(0)	3176(7)
537(3)	1000(0)	3217(1)	526(0)	1004(0)	3200(1)
550(0)	1038(0)	3221(18)	541(5)	1032(0)	3201(0)
592(0)	1041(1)	3227(0)	554(0)	1032(3)	3208(19)
598(0)	1140(0)	3239(4)	554(39)	1122(0)	3208(51)
621(0)	1145(1)	3241(12)	571(40)	1143(2)	3224(11)
653(0)	1152(1)	3244(1)	580(4)	1145(0)	3224(33)
665(6)	1167(2)	3246(2)	588(5)	1156(2)	3229(5)
666(2)	1286(0)	3254(1)	591(0)	1273(0)	3230(0)

Table S68. Harmonic vibrational frequencies (in cm^{-1}) and infrared intensities (in parentheses, in km/mol) for the $C_4H_4NiC_8H_8$ structure **Ni7-48S**

B3PW91			M06-L		
60(0)	794(0)	1302(8)	31(0)	768(0)	1310(6)
107(2)	801(12)	1316(0)	106(2)	796(8)	1311(0)
153(1)	808(9)	1348(17)	148(1)	803(11)	1348(18)
215(0)	809(57)	1367(16)	207(0)	807(49)	1366(9)
221(0)	851(0)	1374(0)	223(0)	840(0)	1371(1)
282(2)	882(12)	1416(0)	282(2)	895(6)	1415(0)
285(2)	909(36)	1434(0)	284(2)	908(39)	1435(0)
296(5)	923(0)	1478(10)	295(4)	910(0)	1457(10)
301(0)	928(10)	1491(17)	315(0)	924(0)	1471(13)
412(2)	938(0)	1708(11)	417(0)	925(14)	1708(16)
417(0)	954(0)	1713(13)	418(0)	937(1)	1714(19)
418(1)	958(9)	3150(0)	418(1)	944(11)	3116(0)
429(1)	962(4)	3152(2)	429(1)	969(0)	3117(9)
461(16)	983(0)	3153(2)	460(14)	969(2)	3121(0)
483(12)	988(16)	3157(0)	475(7)	990(13)	3125(0)
594(16)	1000(1)	3168(33)	588(14)	1001(1)	3133(44)
614(0)	1020(0)	3169(46)	607(0)	1015(0)	3134(127)
640(0)	1150(18)	3178(38)	629(1)	1146(22)	3149(81)
648(1)	1164(2)	3181(30)	641(0)	1159(2)	3152(93)
711(7)	1168(0)	3249(0)	703(1)	1160(1)	3210(0)
720(1)	1169(1)	3265(2)	716(0)	1160(0)	3230(18)
749(1)	1174(0)	3272(3)	724(6)	1165(0)	3237(22)
755(95)	1181(1)	3289(0)	743(74)	1191(0)	3256(3)

Table S69. Harmonic vibrational frequencies (in cm^{-1}) and infrared intensities (in parentheses, in km/mol) for the $C_4H_4NiC_8H_8$ structure **Ni8-48T**

B3PW91			M06-L		
47(0)	760(3)	1326(6)	50(0)	753(2)	1324(5)
61(0)	767(0)	1329(0)	64(0)	769(1)	1342(0)
85(0)	811(5)	1350(3)	80(0)	789(5)	1353(2)
112(0)	826(0)	1361(1)	117(0)	813(0)	1381(4)
144(0)	827(3)	1398(0)	142(0)	855(7)	1398(0)
181(0)	883(5)	1447(4)	184(0)	868(1)	1451(0)
275(1)	884(2)	1457(1)	281(0)	893(1)	1460(0)
306(1)	904(0)	1501(12)	313(1)	907(0)	1507(9)
319(2)	915(2)	1517(14)	326(2)	913(2)	1520(9)
325(0)	918(26)	1553(0)	330(0)	926(12)	1557(1)
340(1)	938(18)	1585(32)	340(1)	930(23)	1591(27)
375(7)	943(11)	3146(2)	406(6)	934(0)	3121(2)
436(10)	949(1)	3151(0)	451(4)	939(12)	3125(1)
462(2)	953(9)	3152(0)	463(1)	950(8)	3125(1)
515(17)	961(1)	3163(1)	513(1)	951(0)	3135(3)
566(14)	975(20)	3164(0)	524(6)	972(15)	3140(4)
571(1)	1107(1)	3176(39)	577(5)	1125(1)	3150(80)
584(61)	1171(0)	3180(61)	588(33)	1161(0)	3155(121)
669(66)	1181(3)	3187(24)	642(66)	1184(3)	3163(46)
687(41)	1190(0)	3249(2)	676(36)	1192(0)	3205(2)
734(0)	1261(8)	3263(2)	721(0)	1264(12)	3222(22)
736(6)	1274(15)	3267(0)	732(0)	1295(7)	3228(7)
740(1)	1303(0)	3286(0)	736(5)	1301(4)	3248(5)

Table S70. Harmonic vibrational frequencies (in cm^{-1}) and infrared intensities (in parentheses, in km/mol) for the $C_4H_4NiC_8H_8$ structure **Ni9-48P**

B3PW91			M06-L		
13(0)	741(0)	1330(0)	-24(0)	747(0)	1334(0)
33(0)	768(22)	1332(1)	60(0)	755(3)	1339(1)
33(0)	768(3)	1332(1)	60(0)	755(3)	1339(1)
39(0)	768(3)	1353(247)	83(0)	775(2)	1410(0)
90(0)	861(0)	1409(0)	83(0)	857(0)	1465(0)
93(0)	890(0)	1460(3)	91(0)	863(0)	1465(0)
93(0)	913(29)	1460(3)	95(0)	913(0)	1526(0)
160(0)	913(29)	1519(1)	164(1)	913(0)	1526(0)
211(0)	919(0)	1519(1)	246(0)	923(16)	1538(7)
211(0)	924(8)	1535(0)	246(0)	923(16)	1546(0)
303(0)	924(8)	1565(0)	305(0)	927(15)	1553(0)
306(0)	930(0)	3164(0)	305(0)	927(15)	3141(0)
330(28)	930(0)	3171(0)	358(14)	929(0)	3146(0)
449(0)	941(0)	3171(0)	450(0)	933(0)	3146(0)
449(0)	945(0)	3182(0)	450(0)	949(0)	3157(0)
452(0)	949(0)	3183(0)	477(0)	963(0)	3157(0)
504(0)	1168(0)	3196(30)	477(0)	1157(0)	3171(86)
539(0)	1191(0)	3196(30)	530(0)	1205(0)	3171(86)
539(0)	1193(0)	3203(5)	541(0)	1210(0)	3178(9)
664(115)	1220(0)	3245(0)	651(114)	1233(0)	3201(0)
688(25)	1268(4)	3260(1)	667(0)	1271(2)	3220(19)
690(0)	1307(639)	3260(1)	675(46)	1332(0)	3220(19)
741(0)	1330(0)	3284(2)	747(0)	1332(0)	3247(8)

Complete Gaussian 09 reference (Reference 23)

M. J. Frisch; G. W. Trucks; H. B. Schlegel; G. E. Scuseria; M. A. Robb; J. R. Cheeseman; G. Scalmani; V. Barone; B. Mennucci; G. A. Petersson; H. Nakatsuji; M. Caricato; X. Li; H. P. Hratchian; A. F. Izmaylov; J. Bloino; G. Zheng; J. L. Sonnenberg; M. Hada; M. Ehara; K. Toyota; R. Fukuda; J. Hasegawa; M. Ishida; T. Nakajima; Y. Honda; O. Kitao; H. Nakai; T. Vreven; J. A. Montgomery; Jr.; J. E. Peralta; F. Ogliaro; M. Bearpark; J. J. Heyd; E. Brothers; K. N. Kudin; V. N. Staroverov; R. Kobayashi; J.Normand; K. Raghavachari; A. Rendell; J. C. Burant; S. S. Iyengar; J. Tomasi; M.Fessi; N. Rega; J. M. Millam; M. Klene; J. E. Knox; J. B. Cross; V. Bakken; C. Adamo; J. Jaramillo; R. Gomperts; R. E. Stratmann; O. Yazyev; A. J. Austin; R.Cammi; C. Pomelli; J. W. Ochterski; R. L. Martin; K. Morokuma; V. G. Zakrzewski; G. A. Voth; P. Salvador; J. J. Dannenberg; S. Dapprich; A. D. Daniels; O. Farkas; J. B. Foresman; J. V. Ortiz; J. Cioslowski; and D. J. Fox, J. A. Gaussian 09, Revision A.02, Gaussian, Inc., Wallingford CT, 2009.
